# Genetic susceptibility markers for a breast-colorectal cancer phenotype: Exploratory results from genome-wide association studies

**DOI:** 10.1371/journal.pone.0196245

**Published:** 2018-04-26

**Authors:** Mala Pande, Aron Joon, Abenaa M. Brewster, Wei V. Chen, John L. Hopper, Cathy Eng, Sanjay Shete, Graham Casey, Fredrick Schumacher, Yi Lin, Tabitha A. Harrison, Emily White, Habibul Ahsan, Irene L. Andrulis, Alice S. Whittemore, Esther M. John, Aung Ko Win, Enes Makalic, Daniel F. Schmidt, Miroslaw K. Kapuscinski, Heather M. Ochs-Balcom, Steven Gallinger, Mark A. Jenkins, Polly A. Newcomb, Noralane M. Lindor, Ulrike Peters, Christopher I. Amos, Patrick M. Lynch

**Affiliations:** 1 Department of Gastroenterology, Hepatology and Nutrition, The University of Texas, MD, Anderson Cancer Center, Houston, United States of America; 2 Department of Biostatistics, The University of Texas, MD, Anderson Cancer Center, Houston, TX, United States of America; 3 Department of Clinical Cancer Prevention, The University of Texas, MD, Anderson Cancer Center, Houston, TX, United States of America; 4 Department of Genetics, The University of Texas, MD, Anderson Cancer Center, Houston, TX, United States of America; 5 Epidemiology and Institute of Health and Environment, The University of Melbourne School of Population and Global Health, Parkville, VIC, Australia; 6 Department of GI Medical Oncology, The University of Texas, MD, Anderson Cancer Center, Houston, TX, United States of America; 7 Department of Epidemiology, The University of Texas, MD, Anderson Cancer Center, Houston, TX, United States of America; 8 Center for Public Health Genomics, University of Virginia, Charlottesville, VA, United States of America; 9 Department of Epidemiology and Biostatistics, Case Western Reserve University, Cleveland, OH, United States of America; 10 Division of Public Health Sciences, Fred Hutchinson Cancer Research Center, Seattle, WA, United States of America; 11 Department of Public Health Sciences, University of Chicago, Chicago, IL, United States of America; 12 Lunenfeld-Tanenbaum Research Institute, Mount Sinai Health System, Department of Molecular Genetics, University of Toronto, Toronto, ON, Canada; 13 Department of Health Research and Policy, Stanford University School of Medicine, Stanford, CA, United States of America; 14 Department of Epidemiology, Cancer Prevention Institute of California, Fremont, CA, United States of America; 15 Department of Epidemiology and Environmental Health, School of Public Health and Health Professions, University at Buffalo, Buffalo, New York, United States of America; 16 Department of Health Sciences Research, Mayo Clinic, Scottsdale, AZ, United States of America; 17 Department of Community and Family Medicine, Dartmouth College, Lebanon, NH, United States of America; Ohio State University Wexner Medical Center, UNITED STATES

## Abstract

**Background:**

Clustering of breast and colorectal cancer has been observed within some families and cannot be explained by chance or known high-risk mutations in major susceptibility genes. Potential shared genetic susceptibility between breast and colorectal cancer, not explained by high-penetrance genes, has been postulated. We hypothesized that yet undiscovered genetic variants predispose to a breast-colorectal cancer phenotype.

**Methods:**

To identify variants associated with a breast-colorectal cancer phenotype, we analyzed genome-wide association study (GWAS) data from cases and controls that met the following criteria: cases (n = 985) were women with breast cancer who had one or more first- or second-degree relatives with colorectal cancer, men/women with colorectal cancer who had one or more first- or second-degree relatives with breast cancer, and women diagnosed with both breast and colorectal cancer. Controls (n = 1769), were unrelated, breast and colorectal cancer-free, and age- and sex- frequency-matched to cases. After imputation, 6,220,060 variants were analyzed using the discovery set and variants associated with the breast-colorectal cancer phenotype at *P*<5.0E-04 (n = 549, at 60 loci) were analyzed for replication (n = 293 cases and 2,103 controls).

**Results:**

Multiple correlated SNPs in intron 1 of the *ROBO1* gene were suggestively associated with the breast-colorectal cancer phenotype in the discovery and replication data (most significant; rs7430339, *P*_discovery_ = 1.2E-04; rs7429100, *P*_replication_ = 2.8E-03). In meta-analysis of the discovery and replication data, the most significant association remained at rs7429100 (*P* = 1.84E-06).

**Conclusion:**

The results of this exploratory analysis did not find clear evidence for a susceptibility locus with a pleiotropic effect on hereditary breast and colorectal cancer risk, although the suggestive association of genetic variation in the region of *ROBO1*, a potential tumor suppressor gene, merits further investigation.

## Introduction

Population-based studies have revealed a strong clustering of breast and colorectal cancer within some families [1, 2]. This clustering has given rise to speculation that there are “breast-colon” cancer susceptibility genes for which there are variants that predispose to co-occurrence of breast and colorectal cancer [3–5]. While co-occurrence of these two common cancers in some families could be due to chance, there is evidence that members of families with segregating germline mutations in *BRCA1*, *BRCA2* and *CHEK2* genes (which are associated with breast cancer) are also at moderately increased risk of colorectal cancer [6–10]. Similarly, families with germline mutations in the *MLH1 or MSH2* genes responsible for Lynch syndrome, or with mutations in the *LKB1/STK11* gene responsible for Peutz-Jeghers syndrome are known to cosegregate the two cancers [11, 12]. However, these relatively rare syndromes cannot explain all the observed familial clustering of breast and colorectal cancer [2].

Daley et al., using a sib-pair analysis of a genome-wide linkage scan of 33 families with a breast-colorectal cancer phenotype, detected multiple linkage peaks, one of them in the region of *BRCA2*. They also detected other novel linked regions, including *D17S1308* on chromosome 17p, in close proximity to the candidate gene hypermethylated in cancer 1 gene, *HIC1* [13]. Two recent population based studies have also found suggestive evidence for a positive genetic correlation between colorectal and breast cancer. Lindstrom and colleagues quantified genetic correlation between different cancer types and found shared heritability of 0.22 for breast and colorectal cancer (*P* = 0.01) [14]. Similarly, Yu and colleagues used the vast Swedish Family-Cancer Database, to identify a modestly increased risk of breast cancer among families of probands affected with colorectal cancer, after excluding cases with a known hereditary predisposition [15]. Further evidence to support the hypothesis that there are additional susceptibility genes is the observation that many families/persons fit our definition of a breast-colorectal cancer phenotype (clustering of breast and colorectal cancer in at least two first- or second-degree relatives in a family, or persons affected with synchronous or metachronous breast and colorectal cancers), but do not have mutations in the known breast or colorectal cancer genes. These families/persons, however, often exhibit features of an inherited predisposition, such as cancer diagnosis at a young age, a Mendelian inheritance pattern, and presence of multiple cancers such as both breast and colorectal cancers in the same person [3]. Such families/persons are suggestive of yet undiscovered susceptibility genes for breast and colorectal cancers and continue to be a challenge for clinical geneticists and their patients because of the difficulty in estimating cancer risk for relatives and defining strategies for future surveillance.

The identification of genetic markers associated with the risk of a breast-colorectal cancer phenotype has clinical implications for prevention and screening/surveillance guidelines. Further, it could aid in identifying families/persons who are at high-risk of an inherited predisposition for breast and colorectal cancer, but do not have any of the known high-penetrance mutations. Therefore the aim of this study was to identify novel susceptibility markers for this understudied phenotype using a genome-wide association study (GWAS) that included discovery and replication phases. We hypothesized that this phenotype is clinically distinct from known hereditary breast and colorectal cancer predisposition syndromes and that unique susceptibility genes influence genetic predisposition to a breast-colorectal cancer phenotype, putting some families and persons at increased risk of both breast and colorectal cancer.

## Materials and methods

### Discovery Phase

#### Data sources

The primary sources were the Colon Cancer Family Registry (CCFR) and the Breast Cancer Family Registry (BCFR) both established to support studies on the etiology, prevention, and clinical management of colorectal and breast cancer, respectively. The CCFR is an international consortium of six sites in North America and Australia for which recruitment of colorectal cancer case families and controls occurred between 1998 and 2012 [16]. The BCFR is a collaboration of six sites in North America and Australia for which recruitment commenced in 1996 [17]. Both CFRs used standardized protocols to collect blood and tissue samples, and questionnaires to collect information about family history, personal and environmental risk factors. By design, both registries are enriched for families with multiple cancer-affected family members.

#### GWAS data

Three separate GWASs were undertaken by the CCFR and BCFR from which data for our study were sourced. All subjects were non-Hispanic white. For the CCFR GWASs, 1189 population-based colorectal cancer cases and 986 unrelated population-based controls were genotyped in Phase 1 in 2009, and 825 cases and 825 same-generation family controls were genotyped in Phase 2 in 2010. The CCFR Phase 1 samples were genotyped on an Illumina Human1M v1 and/or Illumina Human 1M-Duo v3.0 single nucleotide polymorphism (SNP) array, and the Illumina HumanOmni1-Quad v1.0 array for Phase 2 (~50% overlap with the 1M array used in Phase 1) [18, 19]. The BCFR GWAS consisted of population-based case women diagnosed with invasive breast cancer before age 51 years, and women controls between the ages of 20 and 51 years with no history of breast cancer [20]. Genotyping was performed using Illumina 610 Quad and Illumina Cyto12 SNP BeadChip arrays. All GWAS studies excluded cases with a known or likely hereditary predisposition syndrome. The CCFR GWASs excluded colorectal cancer cases with familial adenomatous polyposis; with microsatellite unstable tumors; with tumors for which immunohistochemistry revealed loss of DNA mismatch repair protein; with an *MYH* mutation; or with a known deleterious mismatch repair gene mutation. The BCFR GWAS excluded breast cancer cases with known *BRCA1 or BRCA2* gene mutations.

The data were accessed by submitting a proposal and obtaining approval from the CCFR/BCFR Data Access Committees (CCFR: http://www.coloncfr.org/collaboration; BCFR: http://www.bcfamilyregistry.org/for-researchers/initiate-collaborations). The CFR data can be similarly accessed by researchers with appropriate approvals.

#### Participating studies

All cases and controls for the Discovery Phase were selected from the CCFR or BCFR GWASs and included data from five CCFR and three BCFR sites (Table 1). Cases were defined as: 1) women diagnosed with breast cancer who had one or more first- or second-degree relatives with colorectal cancer; or 2) persons diagnosed with colorectal cancer who had one or more first- or second-degree relatives with breast cancer; or 3) persons diagnosed with both breast and colorectal cancer, irrespective of which cancer was diagnosed first and the time between the two cancer diagnoses. The BCFR GWAS included only women younger than 51 years. Controls were sex- and age-matched (within 5 years) to the cases, unrelated to cases, and not known to have a personal or family history of breast or colorectal cancer. Based on these criteria 1,078 cases and 2,001 controls were used in the Discovery Phase.

**Table 1:** Descriptive characteristics of participating genome-wide association studies in the Discovery and Replication analyses.

**A. Discovery sample**												
	**Total**		**Ontario**		**CSHS/USC**		**Australasian**		**Mayo**		**Seattle**	
	Case	Control	Case	Control	Case	Control	Case	Control	Case	Control	Case	Control
**Total sample, n (%)**	985 (35.8)	1769 (64.2)										
**Female, n (%)**	718 (72.9)	1295 (73.2)										
**Age, mean (SD)**	49.4 (10.8)	51.6 (13.2)										
**Colon Cancer Family Registry**												
**Count, n**	602	991	164	497	71	0	101	188	129	0	137	306
**Female, n (%)**	335 (55.7)	517 (52.2)	96 (58.5)	229 (46.1)	45 (63.4)	0	45 (44.5)	97 (51.6)	71 (55.0)	0	78 (56.9)	191 (62.4)
**Age, mean (SD)**	53.2 (11.1)	59.9 (10.9)	56 (10.8)	61.8 (10.1)	54 (11.2)	0	46 (7.4)	48 (9.1)	50 (9.5)	0	57 (11.8)	64 (7.5)
**Breast Cancer Family Registry**												
**Count, n**	383	778	202	243	43	148	202	387	-	-	-	-
**Female, n (%)**	383 (100)	778 (100)	202 (100)	243 (100)	43 (100)	148 (100)	202 (100)	387 (100)	-	-	-	-
**Age, mean (SD)**	43.2 (6.5)	41.0 (6.6)	42 (6.1)	39 (6.8)	43 (8)	40 (6.9)	45 (6.2)	42 (6)	-	-	-	-
**A. Replication sample**												
	**Total**		**WHI1**		**WHI2**		**VITAL**					
	Case	Control	Case	Control	Case	Control	Case	Control				
Total*, n	293	2103	91	1174	187	805	15	124				
**Age, mean (SD)**	66.4	67.7	67.1 (7.1)	69.3 (6.5)	65.8 (6.3)	65.5 (6.2)	70 (4.8)	67.2 (6.0)				

*All subjects were female; CSHS: Cedars-Sinai Health System, formerly located at USC: the University of Southern California; WHI: Women’s Health Initiative, VITAL: VITamins And Lifestyle cohort

The study was approved by the MD Anderson Institutional Review Board (IRB) and by the respective IRBs of the data providing CFR sites where written informed consent was obtained from all participants prior to collecting the data.

#### Quality control

Standard quality control (QC) measures were implemented on the CCFR and BCFR GWAS datasets [20]. For our study the CCFR dataset consisted of 1,528,306 SNPs (612 cases and 999 controls), and the BCFR dataset consisted of 1,265,521 SNPs (391 cases, 788 controls). For each independent dataset, we applied filters to exclude SNPs with >0.05 missing genotype, minor allele frequency <0.01 and deviation from Hardy Weinberg equilibrium (*P*<1.0E-05) using PLINK v1.07 [21]. For the BCFR data, 1,214,531 SNPs were retained after the initial QC (1790 SNPs failed the Hardy-Weinberg Equilibrium test, and 49,200 SNPs failed the minor allele frequency filter). For the CCFR data, 465,717 common SNPs from two SNP array platforms were retained after the initial QC (998,821 SNPs failed the missing genotype filter, and 579,054 SNPs failed the minor allele frequency filter). The number of CCFR SNPs decreased substantially after removing the SNPs with >0.05 missing genotypes because the CCFR samples were genotyped on two different SNP platforms with ~50% overlap between the platforms.

After filtering, 465,717 CCFR SNPs and 1,214,531 BCFR SNPs remained for a sample of 1,003 cases and 1,787 controls. The datasets from both CFRs were merged and the combined BCFR and CCFR data had 433,277 common SNPs. Additional QC filters were applied for relatedness (a first- or second-degree relative inferred by pairwise allele sharing estimates of identity by descent), mismatch between called and phenotypic sex. There were no sex-discrepant individuals but two cases and four controls were removed due to relatedness (PI HAT > 0.15 after the PLINK IBS test). For this filtered dataset, principal components analysis was performed to remove population outliers using PLINK v1.07 (detailed documentation of the PLINK commands can be found at http://zzz.bwh.harvard.edu/plink/strat.shtml#cluster) [21]; 16 cases and 12 controls were removed because they were 4 or more standard deviations from the centroid (|Z| > 4), and 2 additional controls were removed as outliers after running the smartpca script in Eigenstrat [22]. Finally we had 985 cases and 1787 controls. After removal of the outliers, the PC eigenvectors were recalculated, and the first 6 principal components were included in the association analyses.

#### SNP imputation

The imputation of un-typed SNPs was performed on the merged dataset from 1000 Genomes CEPH data reference panels (Phase 1 Version 3, NCBI build 37 [hg19]) using MaCH 1.0.16 [23] and MiniMac (2012-05-29 release) from 1000 genomes Phase I V3 20101123 release EUR reference panel of 1092 samples [24]. SNPs with quality scores of <0.8 were removed. After imputation, filtering on minor allele frequency>0.01, and imputation quality r^2^>0.8, there were 6,220,060 total imputed SNPs.

#### Discovery analysis

Genome-wide association analyses to identify genetic loci associated with a breast-colorectal cancer phenotype were computed by logistic regression, in an additive genetic model (per allele additive trend test), adjusting for study (CCFR or BCFR), age, sex and six PCs. The analyses for the imputed data were performed using ProbABEL (version 0.4.1; release data August 29, 2013 [25]). We generated quantile-quantile plots and calculated genomic inflation factors to estimate the inflation in test statistics arising from any systematic causes of bias.

### Replication Phase

The top 549 SNPs associated with the breast-colorectal cancer phenotype at *P*<5.0E-04 across 18 chromosomes in the Discovery Phase were selected for replication using the colorectal cancer GWAS studies that are part of the Genetics and Epidemiology of Colorectal Cancer Consortium (GECCO). Details regarding studies participating in GECCO, GWAS genotyping, imputation, and QC have been described elsewhere [19]. Using the same case and control definitions as in the Discovery Phase, 293 cases and 2,103 controls from three GWAS datasets (Women’s Health Initiative [WHI] 1, WHI 2, and VITamins And Lifestyle cohort [VITAL]) were eligible for inclusion in the Replication Phase (Table 1). All cases had a diagnosis of colorectal cancer with a family history of breast cancer in a first-or second-degree relative. Moreover, all cases and controls were non-Hispanic white women because both WHI cohorts consisted of women only, and family history of breast cancer for men in the VITAL cohort was not reported.

Replication analysis was performed using a marginal logistic regression model for each study, followed by a meta-analysis of the study level results. Age, center and three PCs were included in the model.

### Meta-analysis of the discovery and replication phases

Association *P*-values of the discovery data (CCFR and BCFR) and replication data (GECCO) were meta-analyzed using the Genome-wide Association Meta-Analysis Software, GWAMA (v. 2.1), which uses an inverse variance method [26].

### Overlap with variants detected by breast and colorectal GWASs

Published GWAS SNPs associated with breast cancer (n = 99) and with colorectal cancer (n = 39) for Europeans/ non-Hispanic whites were extracted from HaploReg version 3 [accessed on 3/20/18] [27]. We examined results of association analysis for these GWAS SNPs in our Discovery Phase dataset.

### Overlap with functional elements in the genome

Encyclopedia of DNA Elements data were queried to assess overlap with potentially functional genomic characteristics such as DNase I hypersensitivity sites, transcription factor regulatory regions and enhancer elements. Similarly, RegulomeDB [28] and HaploReg version 4 [27] were queried to assess overlap of the SNPs with regulatory genomic regions.

## Results

Data for 985 cases and 1769 controls from 5 CCFR sites and 3 BCFR sites were analyzed in the Discovery Phase (Table 1). The CCFR dataset consisted of 589 cases of colorectal cancer with a family history of breast cancer and 13 cases with breast and colorectal cancer (n = 602 cases). Similarly, the BCFR dataset included 378 breast cancer cases with a family history of colorectal cancer and 5 women with breast and colorectal cancer (n = 383 cases). All cases and controls were non-Hispanic whites. Most participants were women (72.9% of cases and 73.2% of controls). The controls were older than the cases in the overall sample (mean ages 51.5 versus 49.4 years) although the BCFR cases and controls were younger than the CCFR cases and controls (Table 1). The genomic inflation factor was λ = 1.02 (see quantile-quantile plot, S1 Fig).

The most significant association signal from the genome-wide association analysis was for rs12548629 on chromosome 8q22.3 (*P* = 2.5E-07), an intronic SNP in *BAALC* (Table 2, and S2 Fig). We found no genome-wide significant variants (at *P*<5.0E-08). Using a lower threshold (*P*<5.0E-04), we identified 60 suggestive regions/loci across the genome, on multiple chromosomes (chromosomes 1–12 and 16–20). The most significant SNP associations at each locus are listed in Table 2. A majority of the associated variants were in non-coding or intergenic regions. SNPs likely to affect binding or gene expression are indicated by a low score from RegulomeDB [28]. Two potentially functional SNPs, rs11666622 (3’UTR) and rs1468348 (RegulomeDB score 1f) were annotated because they were in linkage disequilibrium (r2>0.9) with the SNP with the smallest P-value in the region. We investigated these loci further using the replication dataset for potential association with a breast-colorectal cancer phenotype. Results of association testing for SNPs with a *P*<5.0E-04 are provided in S1 Table.

**Table 2:** Most significant SNP associations in 60 chromosomal regions, by chromosome, from the Discovery analysis.

Chromosomal region	SNP	Position (build 36/hg18)	Coded Allele	Coded Allele Freq.	OR (95% CI)	*P*	Gene name (GENCODE)	Location	RegulomeDB*
1p13.2	rs116268993	115272760	T	0.99	4.55 (2.07–10.0)	1.36E-05	CSDE1	intronic	7
1p34.3	34655226:T_TG	34655226	R	0.95	2.05 (1.48–2.84))	7.35E-06			
1p36.13	rs72655635	18794898	A	0.83	1.40 (1.20–1.64)	1.24E-05	13kb 5' of KLHDC7A		5
1p36.21	rs6677152	14414502	T	0.66	1.33 (1.16–1.53)	3.62E-05	C1orf196		6
1q31.3	rs80197301	194961694	G	0.98	2.71 (1.63–4.50)	3.43E-05	134kb 5' of AL357932.1		7
1q32.2	rs12403733	209784188	G	0.97	0.49 (0.35–0.69)	3.65E-05	CAMK1G	intronic	4
2q37.1	rs74645168	233943347	G	0.97	2.61 (1.63–4.20)	2.12E-05	INPP5D		5
3p12.3	rs7430339	79766511	G	0.58	0.78 (0.71–0.89)	1.20E-04	ROBO1	intronic	7
3p21.31	rs9836993	46665120	A	0.93	0.62 (0.37–0.78)	5.29E-05	TDGF1	intronic	6
3p25.2	rs62246114	11948649	C	0.66	0.78 (0.66–0.88)	5.46E-05	46kb 3' of Metazoa_SRP		7
3q22.1	rs114398209	133638227	G	0.94	1.78 (1.36–2.33)	1.46E-05	8.8kb 3' of C3orf36		6
3q25.1	rs9834244	151422581	G	0.91	0.59 (0.37–0.73)	8.40E-07	29kb 5' of AADACL2		7
4p16.2	rs34775372	4796443	C	0.92	1.69 (1.33–2.13)	6.39E-06	65kb 5' of MSX1		5
4p15.32	rs1532347	15003896	C	0.94	1.75 (1.33–2.32)	4.81E-05	226bp 5' of AC006296.3		4
4q23	rs139005704	100974971	C	0.99	4.03 (1.87–8.68)	3.06E-05	RP11-15B17.1		6
4q31.1	rs4863620	139876605	A	0.63	1.29 (1.14–1.45)	2.06E-05	RP11-371F15.3		6
4q32.2	rs11736440	163336693	G	0.91	0.66 (0.55–0.8)	1.41E-05	252kb 5' of FSTL5		6
5p13.3	rs253937	31655104	A	0.89	1.51 (1.24–1.83)	2.19E-05	PDZD2		6
5q31.3	rs169087	140283860	C	0.97	0.48 (0.34–0.67)	1.35E-05	PCDHA1	intronic	6
6p23	rs71564305	14108031	G	0.96	0.43 (0.05–0.63)	3.21E-06	9.8kb 5' of CD83		5
6q12	rs112319963	66907171	T	0.99	0.25 (0.13–0.45)	1.53E-06	162kb 5' of AC002485.1		7
6q15	rs72915109	92335740	A	0.91	1.51 (1.31–1.85)	7.20E-05	3.9kb 3' of RP3-433F14.3		7
6q22.31	rs235701	124247963	C	0.81	1.33 (1.18–1.55)	2.79E-04	NKAIN2	intronic	7
6q27	rs4075454	166722486	T	0.51	0.77 (0.66–0.86)	4.64E-06	549bp 5' of PRR18		4
7p12.1	rs4433098	52284370	A	0.58	0.76 (0.64–0.87)	2.53E-05	24kb 5' of RP11-153N17.1		6
7p12.1	rs11981322	52485878	T	0.57	0.78 (0.69–0.88)	4.20E-05	94kb 5' of snoU13		6
7p14.1	rs7794030	38752094	A	0.81	1.43 (1.29–1.65)	1.07E-06	10kb 3' of VPS41		7
7p14.1**	rs1468348*	38767914	T	0.80	1.39 (1.25–1.6)	4.72E-06	VPS41	intronic	1f
7q33	rs59191429	137663901	G	0.93	0.62 (0.38–0.79)	5.37E-05	CREB3L2	intronic	5
8p12	rs6996680	32844577	A	0.63	0.77 (0.65–0.87)	2.25E-05	9.1kb 5' of RP11-11N9.4		6
8p22	rs34793944	17024449	A	0.67	1.27 (1.14–1.44)	2.80E-04	ZDHHC2	intronic	6
8q21.11	rs7461712	75405213	C	0.62	1.28 (1.16–1.44)	3.77E-05	4.1kb 3' of GDAP1		5
8q22.3	rs12548629^#^	104201401	C	0.73	0.71 (0.58–0.81)	2.54E-07	BAALC	intronic	6
9p13.3	35969579:G_GT	35969579	R	0.77	0.74 (0.64–0.85)	3.49E-05			
9p22.2	rs1618634	17962581	G	0.99	2.69 (2.22–4.32)	2.84E-05	165kb 3' of SH3GL2		7
9q21.32	rs13293114	85832384	A	0.64	0.76 (0.64–0.85)	4.35E-06	1.9kb 5' of RP11-439K3.1		6
9q31.3	rs60702108	113047837	T	0.84	0.70 (0.54–0.83)	1.75E-05	18kb 3' of TXNDC8		6
9q34.13	rs59210554	135012819	A	0.77	1.27 (1.13–1.45)	5.72E-04	25kb 5' of NTNG2		5
10p12.2	rs11013837	24284651	T	0.77	1.37 (1.19–1.58)	6.40E-06	KIAA1217	intronic	6
10p13	rs192386529	13664943	A	0.97	0.45 (0.31–0.66)	2.80E-05	PRPF18	intronic	6
10q26.3	rs4751122	131583538	C	0.59	0.78 (0.7–0.88)	2.88E-05	RP11-109A6.4		5
11p15.4	rs1023996	7400790	G	0.58	1.32 (1.18–1.48)	2.27E-06	SYT9	intronic	6
11p15.4	rs55740932	8453025	C	0.94	1.79 (1.36–2.35)	1.72E-05	STK33	intronic	5
11q22.3	rs965505	103739232	C	0.81	1.37 (1.18–1.6)	3.55E-05	RP11-563P16.1		5
12q22	rs7302318	68094969	R	0.85	0.70 (0.59–0.83)	3.17E-05	CRADD		3a
12q24.33	rs7975553	131045130	G	0.57	0.77 (0.68–0.86)	5.95E-06	RIMBP2		6
16p13.2	rs73494614	9515438	A	0.85	1.44 (1.23–1.7)	9.58E-06	13kb 3' of RP11-243A14.1		7
17p13.3	rs4359482	2915699	A	0.55	1.29 (1.15–1.45)	1.59E-05	RAP1GAP2	intronic	6
18q12.2	rs17659787	36600043	T	0.71	0.75 (0.65–0.85)	1.31E-05	3.7kb 5' of 7SK		5
18q21.1	rs34007497	46451073	C	0.54	0.78 (0.69–0.87)	1.26E-05	SMAD7	intronic	4
19p13.12	rs3752185	14828737	G	0.52	0.78 (0.66–0.87)	1.41E-05	ZNF333	intronic	6
19p13.12**	rs11666622*	14830568	T	0.52	0.78 (0.66–0.87)	1.65E-05	ZNF333	3'-UTR	6
19q13.41	rs162277	53030198	C	0.79	1.41 (1.26–1.63)	6.95E-06	706bp 5' of ZNF808		3a
19q13.42	rs2217653	54223164	T	0.73	1.31 (1.15–1.5)	4.30E-05	185bp 5' of MIR520D		5
19q13.42	rs112822051	55547043	C	0.97	2.56 (1.67–3.93)	3.24E-06	GP6	intronic	7
20p11.23	rs113118767	19499434	T	0.99	4.02 (1.99–8.13)	8.97E-06	SLC24A3	intronic	5
20q12	rs6071641	37677661	T	0.58	0.76 (0.63–0.85)	6.44E-06	9.3kb 3' of DHX35		6
20q12*	rs742276*	37679849	G	0.56	0.77 (0.66–0.87)	8.74E-06	11kb 3' of DHX35		2b
20q13.33	rs6027867	59434375	G	0.82	0.74 (0.58–0.86)	8.68E-05	45kb 5' of RP11-151E14.1		7
21q22.2	rs59603367	42433821	T	0.95	0.57 (0.44–0.74)	3.52E-05	80kb 3' of LINC00323		7

*RegulomeDB [28] scores: 1f, likely to affect binding and linked to expression of a gene target; 2b, likely to affect binding; 3a, less likely to affect binding; 4–6, minimal binding evidence; 7, no data.

**Denotes a potentially functional SNP in Linkage Disequilibrium (r2>0.9) with the SNP with the smallest P-value in the region. Other SNPs in the same genomic regions are listed in Supplementary Table 1 (S1 Table).

^#^Most significant SNP in the Discovery data

Replication of the most significant SNP associations (n = 549 SNPs) in 60 regions on 18 chromosomes was performed using 293 cases and 2,103 controls, from 3 studies in GECCO. All cases and controls were women and the mean ages were 66.4 and 67.7 years, respectively (Table 1). The replication analysis of the 549 SNPs revealed multiple correlated SNPs in the 3p12, 9p13.3, and 18q12.2 regions that were significantly associated at *P*<0.05 (most significant SNP, rs7429100, P = 2.8E-03) along with three independent SNPs on 5p13.3, 9p22.2 and 20p11.23 (Table 3). The signal on chromosome 8 around the *BAALC* gene did not replicate (*P* = 0.54).

**Table 3:** Association results for the Discovery, Replication and combined Meta-analysis, for SNPs with *P*<0.05 in the Replication data, by chromosome.

					Discovery		Replication		Meta-analysis*		CCFR only	BCFR only			
SNP	CHR	BP	Coded Allele	Allele Freq	OR	*P*	*P*fixed	*P*het	*P*fixed	*P*het	*P*	*P*	Gene	Variant Type	RegulomeDB
rs7429100	3	79737521	A	0.57	0.79 (0.70–0.89)	1.75E-04	0.003	0.071	1.84E-06	0.141	5.78E-03	4.49E-02	ROBO1	intronic	7
rs9631514	3	79740102	T	0.57	0.79 (0.70–0.89)	1.69E-04	0.003	0.069	1.86E-06	0.138	5.50E-03	4.49E-02	ROBO1	intronic	7
rs7635296	3	79730605	T	0.57	0.79 (0.70–0.90)	1.98E-04	0.003	0.081	1.95E-06	0.156	6.66E-03	4.69E-02	ROBO1	intronic	5
rs7613379	3	79730821	C	0.57	0.79 (0.70–0.90)	1.97E-04	0.003	0.081	1.95E-06	0.156	6.65E-03	4.69E-02	ROBO1	intronic	7
rs6762755	3	79729947	G	0.57	0.79 (0.70–0.90)	2.00E-04	0.003	0.081	1.96E-06	0.156	6.70E-03	4.71E-02	ROBO1	intronic	7
rs7635587	3	79730872	T	0.57	0.79 (0.70–0.90)	1.98E-04	0.003	0.081	1.96E-06	0.156	6.65E-03	4.68E-02	ROBO1	intronic	7
rs6548648	3	79728730	G	0.57	0.79 (0.70–0.90)	2.06E-04	0.003	0.082	2.00E-06	0.157	6.88E-03	4.74E-02	ROBO1	intronic	6
rs9862551	3	79746590	T	0.57	0.79 (0.70–0.89)	1.60E-04	0.004	0.068	2.05E-06	0.139	5.05E-03	4.49E-02	ROBO1	intronic	7
rs7431092	3	79746262	C	0.57	0.79 (0.70–0.89)	1.60E-04	0.004	0.069	2.07E-06	0.140	5.05E-03	4.50E-02	ROBO1	intronic	7
rs9812795	3	79747727	G	0.57	0.79 (0.70–0.89)	1.59E-04	0.004	0.069	2.10E-06	0.140	5.02E-03	4.48E-02	ROBO1	intronic	7
rs9873237	3	79748478	C	0.57	0.79 (0.70–0.89)	1.59E-04	0.004	0.069	2.16E-06	0.140	5.00E-03	4.51E-02	ROBO1	intronic	6
rs10212228	3	79744923	C	0.56	0.79 (0.70–0.90)	1.84E-04	0.003	0.071	2.24E-06	0.141	5.65E-03	4.58E-02	ROBO1	intronic	6
rs4856257	3	79720785	A	0.57	0.80 (0.70–0.90)	2.78E-04	0.003	0.086	2.88E-06	0.163	8.56E-03	5.11E-02	ROBO1	intronic	6
rs3923148	3	79727278	T	0.56	0.80 (0.71–0.90)	2.87E-04	0.003	0.094	3.01E-06	0.175	1.29E-02	4.04E-02	ROBO1	intronic	7
rs7430339	3	79766511	G	0.58	0.80 (0.71–0.89)	1.20E-04	0.008	0.066	3.06E-06	0.141	2.11E-03	5.99E-02	ROBO1	intronic	7
rs9878764	3	79719636	T	0.57	0.80 (0.70–0.90)	2.89E-04	0.003	0.085	3.12E-06	0.162	8.80E-03	5.16E-02	ROBO1	intronic	5
rs4856253	3	79719062	T	0.57	0.80 (0.70–0.90)	2.95E-04	0.003	0.085	3.26E-06	0.162	8.97E-03	5.18E-02	ROBO1	intronic	7
rs6770961	3	79716540	C	0.57	0.80 (0.71–0.90)	3.23E-04	0.003	0.084	3.78E-06	0.161	9.59E-03	5.29E-02	ROBO1	intronic	6
rs12107379	3	79738224	A	0.56	0.79 (0.70–0.90)	2.07E-04	0.007	0.066	4.55E-06	0.140	6.07E-03	4.63E-02	ROBO1	intronic	6
rs13060599	3	79738599	T	0.56	0.79 (0.70–0.90)	2.09E-04	0.009	0.052	5.73E-06	0.115	5.80E-03	4.89E-02	ROBO1	intronic	6
rs9857798	3	79715860	G	0.57	0.80 (0.71–0.91)	4.91E-04	0.005	0.128	8.68E-06	0.236	1.29E-02	6.20E-02	ROBO1	intronic	7
rs7428022	3	79709843	T	0.54	0.79 (0.70–0.89)	2.05E-04	0.014	0.109	9.02E-06	0.218	1.24E-02	2.48E-02	ROBO1	intronic	6
rs7431260	3	79708481	T	0.54	0.79 (0.69–0.89)	1.80E-04	0.017	0.113	9.23E-06	0.224	1.05E-02	2.70E-02	ROBO1	intronic	6
rs9825870	3	79715200	A	0.56	0.80 (0.71–0.91)	4.27E-04	0.007	0.088	9.33E-06	0.176	1.59E-02	4.75E-02	ROBO1	intronic	6
rs6775448	3	79705179	G	0.54	0.79 (0.70–0.90)	2.83E-04	0.011	0.103	9.50E-06	0.208	1.45E-02	2.75E-02	ROBO1	intronic	5
rs9825204	3	79714764	G	0.56	0.80 (0.71–0.91)	4.34E-04	0.007	0.087	9.55E-06	0.174	1.61E-02	4.78E-02	ROBO1	intronic	7
rs7640127	3	79707605	A	0.54	0.79 (0.70–0.90)	2.50E-04	0.013	0.102	1.02E-05	0.206	1.49E-02	2.52E-02	ROBO1	intronic	6
rs4856448	3	79707387	A	0.54	0.79 (0.70–0.90)	2.65E-04	0.013	0.105	1.06E-05	0.212	1.50E-02	2.65E-02	ROBO1	intronic	7
rs7628280	3	79707453	G	0.54	0.79 (0.70–0.90)	2.62E-04	0.013	0.104	1.07E-05	0.211	1.48E-02	2.64E-02	ROBO1	intronic	7
rs9824870	3	79714522	C	0.56	0.80 (0.71–0.91)	4.83E-04	0.007	0.084	1.08E-05	0.169	1.74E-02	4.89E-02	ROBO1	intronic	7
rs4856444	3	79707182	A	0.54	0.79 (0.70–0.90)	2.67E-04	0.013	0.104	1.09E-05	0.210	1.50E-02	2.65E-02	ROBO1	intronic	7
rs9880911	3	79707817	G	0.54	0.79 (0.70–0.90)	2.58E-04	0.014	0.107	1.12E-05	0.214	1.46E-02	2.63E-02	ROBO1	intronic	6
rs4856434	3	79706132	C	0.54	0.79 (0.70–0.90)	2.77E-04	0.013	0.103	1.13E-05	0.208	1.54E-02	2.69E-02	ROBO1	intronic	6
rs9870711	3	79706168	C	0.54	0.79 (0.70–0.90)	2.76E-04	0.013	0.100	1.13E-05	0.202	1.53E-02	2.69E-02	ROBO1	intronic	7
rs4856443	3	79707150	G	0.54	0.79 (0.70–0.90)	2.69E-04	0.014	0.105	1.14E-05	0.212	1.51E-02	2.66E-02	ROBO1	intronic	6
rs4856447	3	79707357	T	0.54	0.79 (0.70–0.90)	2.66E-04	0.014	0.106	1.16E-05	0.213	1.50E-02	2.65E-02	ROBO1	intronic	7
rs4856228	3	79705541	G	0.54	0.79 (0.70–0.90)	2.85E-04	0.013	0.102	1.17E-05	0.207	1.57E-02	2.70E-02	ROBO1	intronic	6
rs4856440	3	79707004	G	0.54	0.79 (0.70–0.90)	2.72E-04	0.014	0.105	1.18E-05	0.212	1.52E-02	2.67E-02	ROBO1	intronic	6
rs4856433	3	79706059	A	0.54	0.79 (0.70–0.90)	2.68E-04	0.015	0.102	1.19E-05	0.206	1.52E-02	2.63E-02	ROBO1	intronic	7
rs6419737	3	79703548	G	0.54	0.80 (0.70–0.90)	3.11E-04	0.014	0.103	1.35E-05	0.209	1.67E-02	2.76E-02	ROBO1	intronic	7
rs9869899	3	79712822	T	0.56	0.81 (0.71–0.91)	6.96E-04	0.008	0.084	1.84E-05	0.170	2.22E-02	5.42E-02	ROBO1	intronic	6
rs7430639	3	79785085	A	0.59	0.81 (0.72–0.90)	2.21E-04	0.030	0.004	1.91E-05	0.011	4.13E-03	5.10E-02	ROBO1	intronic	6
rs7431063	3	79785218	A	0.59	0.81 (0.72–0.90)	2.40E-04	0.031	0.004	2.15E-05	0.011	4.37E-03	5.25E-02	ROBO1	intronic	7
rs4856298	3	79783137	A	0.58	0.81 (0.72–0.90)	2.00E-04	0.040	0.007	2.25E-05	0.018	5.28E-03	4.19E-02	ROBO1	intronic	7
rs6771093	3	79709304	A	0.56	0.81 (0.72–0.92)	9.21E-04	0.008	0.078	2.28E-05	0.156	2.68E-02	5.86E-02	ROBO1	intronic	5
rs7426439	3	79789645	T	0.57	0.80 (0.72–0.91)	3.06E-04	0.026	0.003	2.30E-05	0.009	3.72E-03	6.23E-02	ROBO1	intronic	7
rs9309831	3	79789408	T	0.59	0.81 (0.72–0.91)	2.64E-04	0.031	0.004	2.34E-05	0.011	4.67E-03	5.41E-02	ROBO1	intronic	7
rs3924599	3	79789184	G	0.59	0.81 (0.72–0.91)	2.60E-04	0.032	0.004	2.36E-05	0.011	4.62E-03	5.38E-02	ROBO1	intronic	6
rs1995402	3	79790407	G	0.59	0.81 (0.72–0.91)	2.70E-04	0.031	0.004	2.42E-05	0.011	4.75E-03	5.45E-02	ROBO1	intronic	6
rs1995401	3	79790433	C	0.59	0.81 (0.72–0.91)	2.72E-04	0.031	0.004	2.43E-05	0.011	4.77E-03	5.46E-02	ROBO1	intronic	6
rs7649774	3	79791414	T	0.59	0.81 (0.72–0.91)	2.79E-04	0.033	0.004	2.58E-05	0.010	4.85E-03	5.50E-02	ROBO1	intronic	7
rs7426689	3	79785050	C	0.55	0.81 (0.72–0.91)	5.21E-04	0.031	0.004	4.50E-05	0.011	4.17E-03	1.02E-01	ROBO1	intronic	6
rs253937	5	31655104	A	0.89	1.51 (1.24–1.83)	2.19E-05	0.050	0.024	4.80E-06	0.055	1.87E-02	1.46E-03	PDZD2		6
rs1778181	9	17964230	T	0.99	0.37 (0.23–0.60)	3.47E-05	0.024	0.692	3.72E-06	0.843	2.01E-02	7.47E-03	167kb 3' of SH3GL2		7
rs113609979	9	17961146	T	0.99	0.37 (0.23–0.60)	3.55E-05	0.026	0.722	3.94E-06	0.861	9.70E-03	1.80E-02	164kb 3' of SH3GL2		6
rs145162794	9	17959449	A	0.99	0.38 (0.23–0.61)	4.90E-05	0.027	0.717	5.45E-06	0.859	1.00E-02	2.37E-02	162kb 3' of SH3GL2		6
rs2772690	9	17947742	G	0.99	0.38 (0.23–0.62)	8.62E-05	0.024	0.632	8.29E-06	0.793	3.49E-02	1.22E-02	151kb 3' of SH3GL2		5
rs1755276	9	17942032	A	0.99	0.38 (0.23–0.62)	7.99E-05	0.026	0.634	8.63E-06	0.795	3.63E-02	1.13E-02	145kb 3' of SH3GL2		6
rs2383057	9	17946622	T	0.99	0.38 (0.23–0.62)	8.62E-05	0.028	0.584	9.82E-06	0.742	3.49E-02	1.23E-02	149kb 3' of SH3GL2		6
rs2840779	9	17950343	G	0.99	0.38 (0.23–0.62)	8.69E-05	0.029	0.578	1.02E-05	0.734	3.49E-02	1.21E-02	153kb 3' of SH3GL2		6
rs2840782	9	17947425	A	0.99	0.38 (0.23–0.62)	8.63E-05	0.030	0.577	1.03E-05	0.733	3.49E-02	1.23E-02	150kb 3' of SH3GL2		7
rs2772692	9	17949040	G	0.99	0.38 (0.23–0.62)	8.67E-05	0.030	0.578	1.04E-05	0.733	3.49E-02	1.22E-02	152kb 3' of SH3GL2		7
rs2811824	9	17948254	C	0.99	0.38 (0.23–0.62)	8.63E-05	0.030	0.575	1.05E-05	0.730	3.49E-02	1.22E-02	151kb 3' of SH3GL2		5
rs10217546	9	35986887	C	0.54	0.82 (0.73–0.92)	8.89E-04	0.040	0.830	8.24E-02	0.006	2.59E-02	3.21E-02	29kb 5' of OR2S2		6
rs72886417	18	36602286	G	0.71	0.75 (0.66–0.85)	1.31E-05	0.029	0.168	1.13E-06	0.298	8.17E-06	4.31E-02	6kb 5' of 7SK		6
rs17659787	18	36600043	T	0.71	0.75 (0.65–0.85)	1.31E-05	0.028	0.167	1.14E-06	0.296	8.28E-06	4.29E-02	3.7kb 5' of 7SK		5
rs72904570	18	36585235	G	0.72	0.75 (0.65–0.85)	1.62E-05	0.028	0.164	1.37E-06	0.293	1.28E-05	3.73E-02	11kb 3' of 7SK		6
rs72886457	18	36643966	C	0.70	0.76 (0.67–0.86)	1.99E-05	0.036	0.250	2.09E-06	0.412	2.86E-05	3.69E-02	30kb 3' of U6		7
rs113118767	20	19499434	T	0.99	4.02 (1./99-8.13)	8.97E-06	0.026	0.290	1.29E-01	0.000	4.94E-03	3.72E-03	SLC24A3	intronic	5

*Meta-analysis of BCFR, CCFR, WHI1, WHI2 and VITAL GWAS data; CCFR, Colon Cancer Family Registry; BCFR, Breast Cancer Family Registry; SNP, single nucleotide polymorphism; CHR, chromosome number; BP, location in base-pairs; Freq, frequency; OR, odds ratio; CI, confidence interval; *P*fixed, *P* value for fixed-effects meta-analysis; *P*het, *P* value for heterogeneity.

The meta-analysis of discovery and replication results did not identify a genome-wide significant signal (smallest meta *P* was for rs7429100 on chromosome 3p12.3, *P* = 1.84E-06; Table 3). The strongest suggestive association from the combined dataset (on 3p12.3) was for several highly correlated SNPs in the region of the roundabout guidance receptor 1 gene, *ROBO1* (*P* ranging between 1.8–9.6E-06; Table 3). The *ROBO1* signal was driven by 53 highly correlated SNPs (r^2^: 0.78–1.0) in intron 1.

Association results for the published GWAS hits for breast and colorectal cancer were nominally significant at *P* <0.05 for 4/39 colorectal cancer-associated SNPs and 5/99 breast cancer-associated SNPs (S2 Table and S3 Table). The nominally significant SNPs included colorectal cancer GWAS SNPs in *SMAD7*, *C11orf93*, *SCG5*, and *ATF1*, and breast cancer SNPs related to *CDKN2B-AS1*, *FGFR2*, *GDI2*, *CDYL2* (S2 Table and S3 Table).

The *in silico* functional analysis of the *ROBO1* SNPs displayed a range of altered binding motifs: rs9878764 had protein binding activity with CEBPB (CCAAT/Enhancer Binding Protein Beta), but no breast or colorectal tissue specific expression quantitative trait loci (eQTL) were associated with any of the *ROBO1* SNPs (S4 Table). However, *ROBO1* gene expression is fairly ubiquitous across multiple human tissues (S3 Fig), and *ROBO1* is frequently mutated across almost all cancer types (S4 Fig), including breast and colon.

## Discussion

In this study, we analyzed GWAS data from the Colon and Breast Cancer Family Registries in cases enriched for family history of breast and colorectal cancer, and controls, to agnostically identify novel markers of genetic susceptibility for the joint breast-colorectal cancer phenotype. Our cases were diagnosed at a younger age (mean age, 49.4 years), which coupled with their cancer family histories, favors the likelihood of a genetic predisposition. Our main findings include a suggestive association of a cluster of SNPs in *ROBO1* with the breast-colorectal cancer phenotype, although none of the SNPs were genome-wide statistically significant.

There has been long interest and debate around possible genetic susceptibility to a distinct breast-colorectal cancer phenotype. The clustering of breast and colorectal cancer in families was described as early as in 1972 by Lynch *et al*. who found that some families where many members had breast cancer also had a high predisposition to colorectal cancer [29]. Subsequently, the idea of a distinct hereditary breast and colorectal cancer phenotype (HBCC) was proposed by Meijers-Heijboer and colleagues, when they found a significant association of the *CHEK2* 1100delC mutation with HBCC using a subset of familial breast cancer families that did not carry the *BRCA1* or *BRCA2* mutations [10]. However, a larger study did not confirm the HBCC syndrome as a separate entity linked to the *CHEK2* 1100delC mutation, suggesting that HBCC could be due to chance or yet undiscovered genes [4]. A similar message was conveyed in a commentary by Lipton and colleagues, who suggested that other than the known clinical syndromes, many breast-colorectal cancer families probably result from chance clustering of two common cancers or through a genetic predisposition to one of the cancers and chance co-occurrence of the other. However, they acknowledged that there are families that present with evidence of genetic disease not accounted for by known genes or chance. They also posited that it may be difficult to identify potential breast-colorectal cancer genes, suggesting a candidate gene analysis approach as the most suited (at the time, in the pre-GWAS era) [3]. GWAS data has now allowed us to look beyond candidate genes to use genetic variation across the genome to try to identify breast-colorectal cancer susceptibility loci.

In the present study, no genome-wide significant loci were detected in the Discovery Phase but we found some suggestive associations, across multiple chromosomes, the most promising on chromosome 8q22.3, overlying the *BAALC* gene. However, of the 549 SNPs at 60 loci tested for replication, *BAALC* SNPs did not replicate but P-values <5.0E-03 were found for several correlated SNPs in the region of chromosome 3p12.3; the *ROBO1* gene. Although suggestive, the association signal did not meet the Bonferroni multiple testing threshold, (*P*<9.1E-05 for testing 549 SNPs, or <8.3E-04 if considering 60 loci) but the cluster of SNPs in intron 1 of the *ROBO1* gene also had the smallest P-values from meta-analysis of the discovery and replication datasets (*P*_*smallest*_ = 1.84E-06; rs7429100).

*ROBO1* merits follow-up as it may have a potential functional role in breast-colorectal carcinogenesis. A transmembrane receptor of the immunoglobulin family, *ROBO1*interacts with *SLIT2* (Slit Guidance Ligand 2) to regulate many biological functions, is differentially expressed in human cancers, and has a possible role as a tumor suppressor gene [30]. Studies have found that low ROBO1 expression is an adverse prognostic factor for breast cancer [31, 32] and might play a role in the pathogenesis of colorectal cancer [33].

Evidence in support of *ROBO1* as a susceptibility gene for a hereditary breast-colorectal cancer (HBCC) phenotype comes from a study by Villacis and colleagues [34]. The aim of their study was to identify genomic alterations (copy number variations, CNVs) related to cancer predisposition in patients with a suggestive HBCC phenotype who did not carry high risk mutations in the major genes known to be implicated in hereditary breast or colorectal cancer (i.e., patients who met HBCC criteria as defined by Naseem et al. [4]). The authors identified a *ROBO1* germline deletion in intron 4, spanning 37.470 kb (chr3:78,990,568–79,028,038 hg18), in three unrelated cases out of 113 HBCC patients. Pathogenicity of the deletion was supported by familial co-segregation with disease, its rarity in public CNV databases, and in silico evidence of the deletion having a functional role due to the presence of several enhancers and a histone marker in the deleted region. Notably, the authors reported that direct sequencing did not reveal any pathogenic point mutations in *ROBO1*. From our data and analyses, the association signal for *ROBO1*, comprised a cluster of 53 SNPs in intron 1, spanning 87.866 kb (79,703,548–79,791,414 hg 18) and the SNP closest to the deletion was located 675.51 kb away from the deletion. Furthermore, unlike the rare deletion identified by Villacis and colleagues [34], these were common SNPs with a minor allele frequency ranging between 0.39–0.46.

Of the published GWAS SNPs associated with colorectal cancer risk and breast cancer risk, our analysis of the combined breast-colorectal dataset found that only a few SNPs were nominally significant at *P*<0.05. The *SMAD7* SNP rs4939827, identified as a colorectal cancer risk SNP [35] was the most significantly associated SNP in our breast-colorectal data (*P* = 1.85E-04) with a consistent direction of association for the risk allele (T). To our knowledge, there is no published evidence of association of this SNP with breast cancer risk, although a role of *SMAD7* in the modulation of cancer growth and progression has been suggested for many cancers, including breast cancer [36]. Among the GWAS SNPs known to be associated with breast cancer risk, notable were two *FGFR2* SNPs (rs298175 and rs2981582) [37, 38] that were nominally significant in our breast-colorectal data. Genetic alternations in *FGFR2* have been found to be associated with cancers other than breast but we have not found any association of *FGFR2* SNPs with colorectal cancer in the published literature.

Unlike the present study, which used a familial clustering approach used to identify cases, other studies have applied a meta-analysis approach to large GWAS datasets to identify common genetic susceptibility variants across multiple cancers. For example, using colorectal cancer and endometrial cancer genome-wide data for ~13,000 cases unselected for age of disease onset or family history, and ~40,000 controls, Cheng and colleagues identified two novel polymorphisms, rs3184504 in the *SH2B3* gene and rs12970291 near the *TSHZ1* gene with evidence for a shared colorectal and endometrial cancer predisposition [39]. Another study by Hung and colleagues reported that the same *SH2B3* SNP on chr12q24 was associated with lung, colorectal and breast cancer [40]. In our study, however, we did not find an association of rs3184504 or any other variant in the chr12q24 region, with the breast-colorectal cancer phenotype. Furthermore, a recent comprehensive analysis of pleiotropic associations across five cancers using 61,851 cases and 61,850 controls did not find any evidence of pleiotropy across breast and colorectal cancer [41], which suggests that these two cancers may not have a common genetic susceptibility mechanism.

The strengths of the present study include using an agnostic approach to identify genetic susceptibility loci, and its enrichment for genetic susceptibility through the incorporation of early cancer onset (for breast cancer) and family history to define the breast-colorectal cancer phenotype. Furthermore, any breast-colorectal GWAS signal was less likely to be due to syndromic cases since all cases carrying *BRCA1* or BRCA2 or known colorectal cancer susceptibility gene mutations were excluded from the GWAS. Despite this, our study could have had limited statistical power to detect alleles with small true effect sizes especially if they are rare. Our study was powered to detect risk alleles with a frequency greater than 10% and a per-allele odds ratio of 1.7. Although this detectable odds ratio is high for a GWAS, we reasoned that because the cases had a family history, the frequency of the risk allele could be higher than it would be for unselected cases [42]. Study power could also have been reduced because data were from different GWAS platforms, however, imputation of genetic variants after merging datasets allowed us to maximize genetic markers for our analyses. Another potential limitation was that the breast cancer cases being younger had less time to be diagnosed with CRC and similarly, the relatives had fewer person-years at risk for CRC, than cases that had incident CRC and were older. Furthermore, although the cases were identified based on family history, the inclusion criteria regarding the number of relatives affected or case subjects’ age at cancer onset were not stringent. This is in contrast to the HBCC criteria suggested by Naseem and colleagues, which included age of colorectal or breast cancer onset <50 years as a defining feature for the affected or relative [4]. We used the relaxed criteria to obtain a larger sample size and increase the power to detect an association, as this was an exploratory analysis, however power may be reduced due to smaller effect sizes. Future studies of affected families with stronger clustering of breast and colorectal cancer, might reveal a specific genetic signal. There is also the possibility of recall bias in the capture of family history between cases and controls, however, if the controls had unreported breast or colorectal cancer-affected first- or second-degree relatives, it may lead to type 2 (false-negatives) rather than type 1 error. Finally, the study was limited by the lack of availability of a large dataset for replication. While many thousands of people have been genotyped in the GWASs, these studies provide only limited phenotype data, and most notably, lack data on cancer family history. Lack of replication could also be because the replication data did not closely resemble the discovery data due to the inclusion of only women with a higher mean age in the replication series, in contrast to the discovery data which included both men and women who were relatively younger.

This analysis, aimed at elucidating genes/regions associated with a pleiotropic effect for breast and colorectal cancer risk, did not show a clear susceptibility locus for this phenotype. This raises the possibility that aggregation of these cancers within families may be due to chance co-occurrence of two common cancers. However, since germline variation in the region of *ROBO1* was suggestive of an association with breast-colorectal cancer risk, and given mounting evidence for the role of *ROBO1* as a tumor suppressor gene, further investigation of this association may be warranted.

## Supporting information

S1 Fig Quantile-quantile plot of genotyped and imputed data. (DOCX)

S2 Fig Manhattan plot (Discovery data). (DOCX)

S3 Fig ROBO1 gene expression across multiple human tissues, including breast and colon. (DOCX)

S4 Fig *ROBO1* mutations across different cancer types, including breast and colorectal. (DOCX)

S1 Table SNPs with P<5x10^-4^ (n = 549) in the Discovery Phase, and their Discovery, Replication, Meta-analysis, and stratified (by CCFR/BCFR) P-values, by chromosome. (DOCX)

S2 Table Odds ratio (OR) estimates with 95% confidence interval (CI) for association of selected colorectal cancer GWAS SNPs with the breast-colorectal cancer phenotype. (DOCX)

S3 Table Odds ratio (OR) estimates with 95% confidence interval (CI) for association of selected breast cancer GWAS SNPs with the breast-colorectal cancer phenotype. (DOCX)

S4 Table Functional annotation of the *ROBO1* variants. (DOCX)
