# Supplementary figures and images for: Genetic susceptibility markers for a breast-colorectal cancer phenotype: Exploratory results from genome-wide association studies
Source: PLoS One. 2018 Apr 26;13(4):e0196245. doi: 10.1371/journal.pone.0196245 (PMC5919670; doi:10.1371/journal.pone.0196245)

**S1 Fig. Quantile-quantile plot of genotyped and imputed data.**


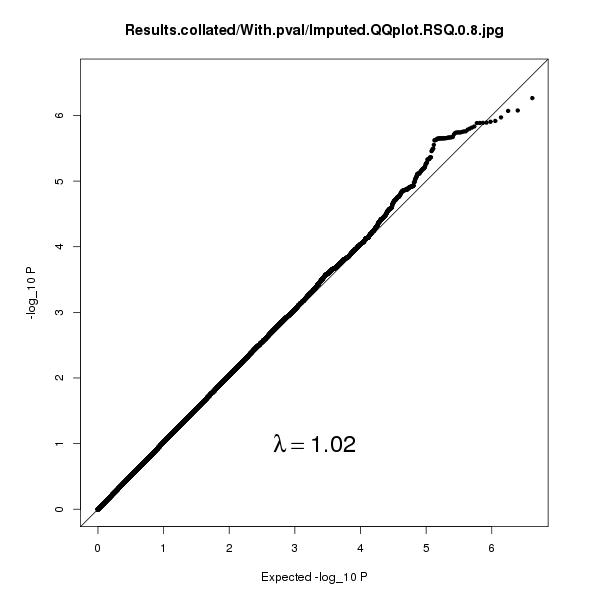

Supplement: S1 Fig — (DOCX) [file pone.0196245.s001.docx]
