# Supplementary material for: Genetic susceptibility markers for a breast-colorectal cancer phenotype: Exploratory results from genome-wide association studies
Source: PLoS One. 2018 Apr 26;13(4):e0196245. doi: 10.1371/journal.pone.0196245 (PMC5919670; doi:10.1371/journal.pone.0196245)

**S2 Fig. Manhattan plot (Discovery data),** imputed, r2=0.8 (# of SNPs = 6220060).

Plot shows –log10-transformed P values for genotyped and imputed SNPs with respect to their physical positions.


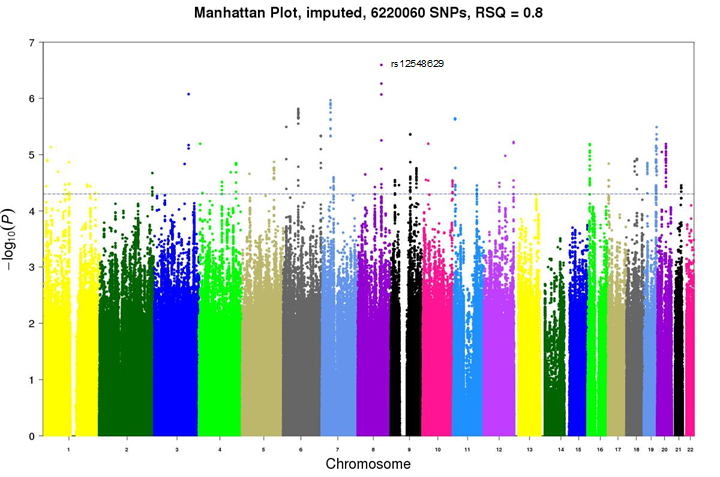

Supplement: S2 Fig — (DOCX) [file pone.0196245.s002.docx]
