# Supplementary material for: Genetic susceptibility markers for a breast-colorectal cancer phenotype: Exploratory results from genome-wide association studies
Source: PLoS One. 2018 Apr 26;13(4):e0196245. doi: 10.1371/journal.pone.0196245 (PMC5919670; doi:10.1371/journal.pone.0196245)
Supplement: S3 Fig — (DOCX) [file pone.0196245.s003.docx]

**S3 Fig. ROBO1 gene expression across multiple human tissues, including breast and colon.**


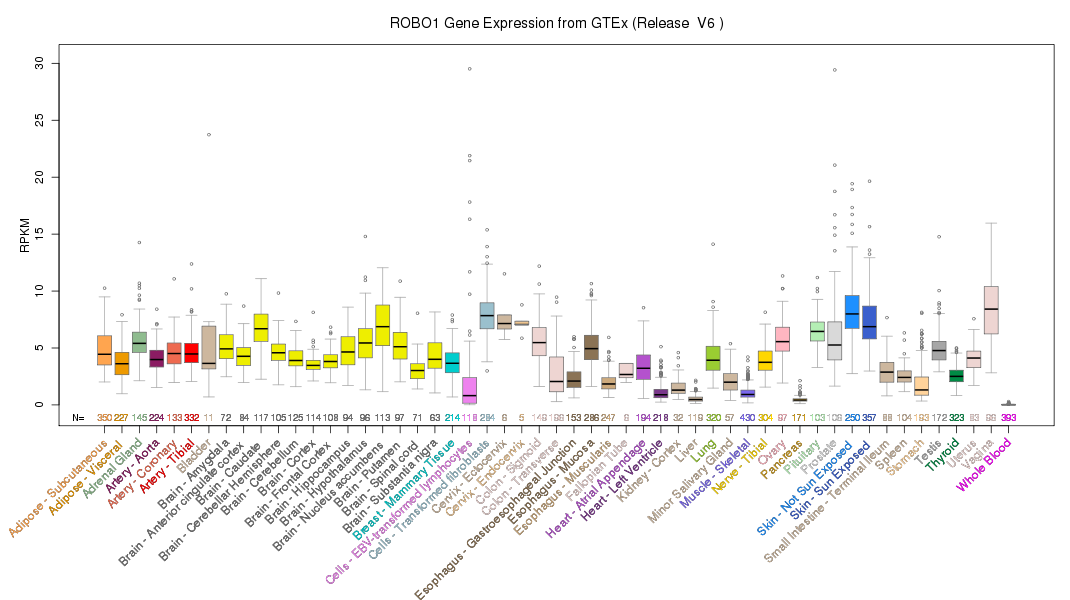


Source: <https://genome.ucsc.edu/cgi-bin/hgc?hgsid=612648669_24i9M1zuDjlH6Osp9aYraDk8M7Kg&c=chr3&l=78649370&r=78749468&o=78646389&t=79816965&g=gtexGene&i=ROBO1>
