# Supplementary material for: Genetic susceptibility markers for a breast-colorectal cancer phenotype: Exploratory results from genome-wide association studies
Source: PLoS One. 2018 Apr 26;13(4):e0196245. doi: 10.1371/journal.pone.0196245 (PMC5919670; doi:10.1371/journal.pone.0196245)
Supplement: S4 Fig — (DOCX) [file pone.0196245.s004.docx]

**S4 Fig. *ROBO1* mutations across different cancer types, including breast and colorectal.**


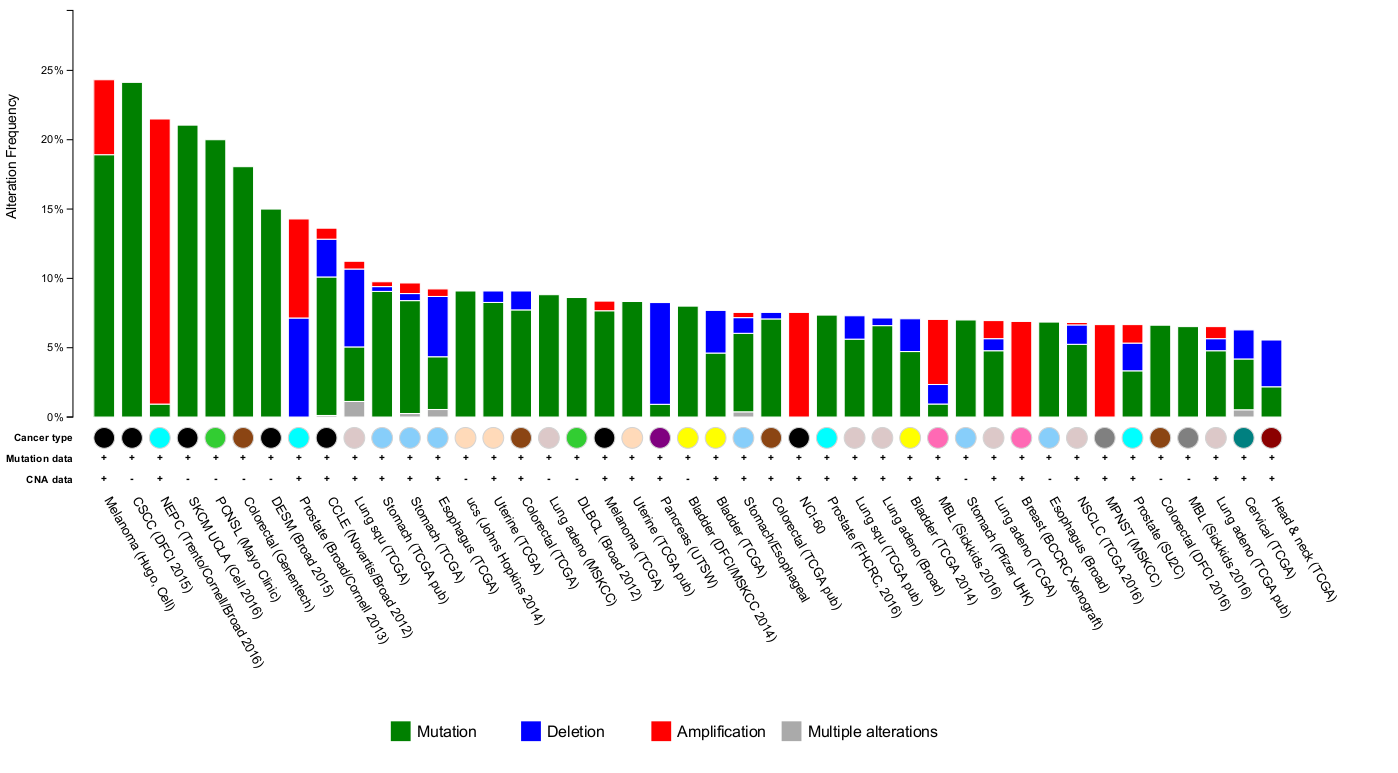


Source: [www.cbioportal.org](http://www.cbioportal.org)
