# Supplementary material for: Genetic susceptibility markers for a breast-colorectal cancer phenotype: Exploratory results from genome-wide association studies
Source: PLoS One. 2018 Apr 26;13(4):e0196245. doi: 10.1371/journal.pone.0196245 (PMC5919670; doi:10.1371/journal.pone.0196245)
Supplement: S1 Table — (DOCX) [file pone.0196245.s005.docx]

**S1 Table**. **SNPs with *P*<5x10^-4^ (n=549) in the Discovery Phase, and their Discovery, Replication, Meta-analysis, and Stratified (by CCFR/BCFR) *P*-values, by Chromosome**

|  | | | | | | **DISCOVERY** | | **REPLICATION** | | | **META-ANALYSIS**  **(CCFR/BCFR+WHI1,2,VITAL)** | | | **CCFR**  only | **BCFR**  Only |
| --- | --- | --- | --- | --- | --- | --- | --- | --- | --- | --- | --- | --- | --- | --- | --- |
| SNP^1^ | CHR^2^ | BP^3^ | Coded  allele | Alternate  allele | Coded allele frequency | OR (95% CI)^4^ | *P* | *P*  fixed^5^ | *P*  random^6^ | *P*  het^7^ | *P*  fixed^5^ | *P*  random^6^ | *P*  het^7^ | *P* CCFR^8^ | *P* BCFR^9^ |
| rs6677152 | 1 | 14414502 | T | C | 0.66 | 1.33 (1.16-1.53) | 3.62E-05 | 0.636 | 0.794 | 0.29 | 0.0017573 | 0.454 | 0.02 | 2.31E-02 | 2.28E-03 |
| rs72655635 | 1 | 18794898 | A | G | 0.83 | 1.4 (1.2-1.64) | 1.24E-05 | 0.306 | 0.306 | 0.39 | 2.75E-05 | 0.006 | 0.28 | 7.78E-02 | 1.62E-04 |
| rs12143755 | 1 | 18795442 | A | G | 0.83 | 1.4 (1.2-1.64) | 1.30E-05 | 0.352 | 0.355 | 0.35 | 3.58E-05 | 0.017 | 0.23 | 8.05E-02 | 1.60E-04 |
| 1:34655226:T_TG | 1 | 34655226 | R | I | 0.95 | 2.05 (1.48-2.84) | 7.35E-06 | 0.421 | 0.421 | 0.96 | 4.52E-03 | 0.649 | 0.01 | 1.73E-03 | 2.69E-03 |
| rs114405179 | 1 | 66284038 | G | C | 0.98 | 0.39 (0.26-0.61) | 2.01E-05 | 0.554 | 0.554 | 0.79 | 1.02E-04 | 0.006 | 0.31 | 3.21E-03 | 1.15E-03 |
| rs6697132 | 1 | 97682026 | A | G | 0.63 | 0.78 (0.69-0.88) | 3.39E-05 | 0.967 | 0.954 | 0.31 | 3.81E-04 | 0.393 | 0.08 | 1.76E-02 | 4.30E-04 |
| rs885622 | 1 | 97691005 | C | T | 0.63 | 0.78 (0.69-0.88) | 4.21E-05 | 0.989 | 0.957 | 0.33 | 4.95E-04 | 0.4 | 0.08 | 2.18E-02 | 4.27E-04 |
| 1:114425702:A_AG | 1 | 114425702 | R | I | 0.98 | 0.43 (0.28-0.64) | 4.95E-05 | 0.779 | 0.857 | 0.28 | 5.11E-04 | 0.251 | 0.08 | 6.73E-01 | 1.23E-05 |
| rs222495 | 1 | 115136771 | A | G | 0.9 | 1.54 (1.25-1.9) | 3.38E-05 | 0.503 | 0.565 | 0.31 | 1.56E-04 | 0.07 | 0.18 | 3.28E-03 | 6.42E-03 |
| rs12021674 | 1 | 115162823 | C | T | 0.9 | 1.54 (1.25-1.89) | 3.31E-05 | 0.488 | 0.543 | 0.31 | 1.34E-04 | 0.056 | 0.2 | 2.99E-03 | 6.67E-03 |
| rs116268993 | 1 | 115272760 | T | G | 0.99 | 4.55 (2.07-10) | 1.36E-05 | 0.501 | 0.501 | 0.75 | 3.77E-04 | 0 | 0.46 | 7.23E-02 | 1.81E-03 |
| rs183326733 | 1 | 115354449 | G | A | 0.99 | 4.26 (1.94-9.32) | 3.27E-05 | 0.459 | 0.813 | 0.34 | 6.19E-04 | 0.043 | 0.25 | 1.19E-01 | 1.82E-03 |
| rs1628124 | 1 | 115643025 | C | T | 0.96 | 1.98 (1.4-2.81) | 4.78E-05 | 0.571 | 0.571 | 0.92 | 4.23E-04 | 0 | 0.4 | 1.58E-01 | 3.87E-04 |
| rs1286350 | 1 | 115644138 | T | C | 0.96 | 1.98 (1.4-2.81) | 4.75E-05 | 0.57 | 0.57 | 0.92 | 4.18E-04 | 0 | 0.4 | 1.58E-01 | 3.85E-04 |
| rs190101485 | 1 | 194925547 | T | C | 0.98 | 2.72 (1.63-4.54) | 3.71E-05 | 0.479 | 0.479 | 0.77 | 6.17E-03 | 0.519 | 0.04 | 4.68E-03 | 5.28E-03 |
| rs80197301 | 1 | 194961694 | G | A | 0.98 | 2.71 (1.63-4.5) | 3.43E-05 | 0.488 | 0.488 | 0.67 | 5.46E-03 | 0.504 | 0.04 | 4.99E-03 | 5.10E-03 |
| rs77439275 | 1 | 194962687 | G | A | 0.98 | 2.71 (1.63-4.5) | 3.43E-05 | 0.5 | 0.5 | 0.66 | 5.23E-03 | 0.493 | 0.04 | 5.01E-03 | 5.08E-03 |
| rs17014889 | 1 | 209784002 | T | C | 0.97 | 0.49 (0.35-0.69) | 3.68E-05 | 0.635 | 0.635 | 0.79 | 1.31E-04 | 0.002 | 0.35 | 1.09E-03 | 4.48E-03 |
| rs12403733 | 1 | 209784188 | G | A | 0.97 | 0.49 (0.35-0.69) | 3.65E-05 | 0.635 | 0.635 | 0.79 | 1.31E-04 | 0.002 | 0.35 | 1.09E-03 | 4.47E-03 |
| rs9727104 | 1 | 232259782 | C | T | 0.67 | 0.78 (0.69-0.88) | 4.77E-05 | 0.615 | 0.615 | 0.98 | 1.54E-03 | 0.461 | 0.08 | 9.03E-04 | 3.47E-02 |
| rs60486020 | 2 | 233931672 | G | A | 0.97 | 2.31 (1.5-3.55) | 4.98E-05 | 0.61 | 0.61 | 0.57 | 4.99E-04 | 0.024 | 0.28 | 4.00E-02 | 1.86E-03 |
| rs73094968 | 2 | 233933122 | G | A | 0.97 | 2.33 (1.52-3.57) | 3.83E-05 | 0.591 | 0.591 | 0.58 | 3.98E-04 | 0.022 | 0.28 | 3.73E-02 | 1.47E-03 |
| rs73094970 | 2 | 233933148 | C | T | 0.97 | 2.33 (1.52-3.57) | 3.84E-05 | 0.594 | 0.594 | 0.6 | 4.08E-04 | 0.021 | 0.28 | 3.74E-02 | 1.47E-03 |
| rs11674347 | 2 | 233941388 | A | G | 0.97 | 2.35 (1.52-3.61) | 3.93E-05 | 0.522 | 0.522 | 0.58 | 3.76E-04 | 0.018 | 0.27 | 2.89E-02 | 1.66E-03 |
| rs74645168 | 2 | 233943347 | G | A | 0.97 | 2.61 (1.63-4.2) | 2.12E-05 | 0.559 | 0.559 | 0.61 | 4.28E-04 | 0.044 | 0.2 | 2.10E-02 | 1.54E-03 |
| rs73094988 | 2 | 233947361 | A | C | 0.98 | 2.73 (1.62-4.58) | 4.52E-05 | 0.753 | 0.753 | 0.79 | 1.26E-03 | 0.073 | 0.22 | 3.15E-02 | 2.66E-03 |
| rs13083227 | 3 | 11948258 | C | A | 0.66 | 1.27 (1.13-1.44) | 5.96E-05 | 0.355 | 0.355 | 0.39 | 1.12E-03 | 0.714 | 0.05 | 3.52E-02 | 3.17E-03 |
| rs62246114 | 3 | 11948649 | C | T | 0.66 | 1.28 (1.14-1.45) | 5.46E-05 | 0.35 | 0.35 | 0.39 | 1.15E-03 | 0.714 | 0.04 | 3.65E-02 | 2.71E-03 |
| rs9819159 | 3 | 46664908 | T | C | 0.92 | 1.58 (1.25-2) | 7.18E-05 | 0.137 | 0.137 | 0.71 | 2.48E-02 | 0.95 | 0.01 | 7.91E-03 | 7.35E-03 |
| rs9836993 | 3 | 46665120 | A | G | 0.93 | 1.62 (1.28-2.07) | 5.29E-05 | 0.106 | 0.106 | 0.38 | 2.82E-02 | 0.989 | 0 | 6.90E-03 | 6.43E-03 |
| rs72904287 | 3 | 46665265 | G | A | 0.92 | 1.58 (1.25-1.99) | 7.22E-05 | 0.091 | 0.091 | 0.59 | 3.56E-02 | 0.926 | 0 | 7.79E-03 | 7.57E-03 |
| rs11928922 | 3 | 46665839 | G | T | 0.92 | 1.58 (1.25-1.99) | 7.25E-05 | 0.092 | 0.092 | 0.59 | 3.54E-02 | 0.928 | 0 | 7.79E-03 | 7.60E-03 |
| rs9880885 | 3 | 46666347 | G | T | 0.92 | 1.58 (1.25-1.99) | 7.29E-05 | 0.092 | 0.092 | 0.59 | 3.52E-02 | 0.928 | 0 | 7.79E-03 | 7.67E-03 |
| rs9829227 | 3 | 46666521 | T | C | 0.92 | 1.58 (1.25-1.99) | 7.29E-05 | 0.091 | 0.091 | 0.59 | 3.52E-02 | 0.927 | 0 | 7.79E-03 | 7.65E-03 |
| rs9810013 | 3 | 46666793 | G | A | 0.92 | 1.58 (1.25-1.99) | 7.27E-05 | 0.093 | 0.093 | 0.59 | 3.50E-02 | 0.929 | 0 | 7.76E-03 | 7.67E-03 |
| rs3796367 | 3 | 46666953 | A | G | 0.92 | 1.58 (1.25-1.99) | 7.30E-05 | 0.093 | 0.093 | 0.59 | 3.46E-02 | 0.928 | 0 | 7.66E-03 | 7.86E-03 |
| rs3796368 | 3 | 46667078 | T | C | 0.92 | 1.58 (1.25-1.99) | 7.32E-05 | 0.093 | 0.093 | 0.59 | 3.45E-02 | 0.928 | 0 | 7.67E-03 | 7.88E-03 |
| rs3796369 | 3 | 46667110 | A | G | 0.92 | 1.58 (1.25-1.99) | 7.32E-05 | 0.093 | 0.093 | 0.59 | 3.45E-02 | 0.928 | 0 | 7.67E-03 | 7.89E-03 |
| rs3796370 | 3 | 46667183 | C | A | 0.92 | 1.58 (1.25-1.98) | 7.34E-05 | 0.093 | 0.093 | 0.59 | 3.45E-02 | 0.928 | 0 | 7.67E-03 | 7.91E-03 |
| rs9834713 | 3 | 46667688 | T | C | 0.92 | 1.58 (1.25-1.98) | 7.34E-05 | 0.086 | 0.086 | 0.62 | 3.66E-02 | 0.913 | 0 | 7.64E-03 | 7.97E-03 |
| rs9835025 | 3 | 46667880 | T | C | 0.92 | 1.57 (1.25-1.98) | 7.36E-05 | 0.086 | 0.086 | 0.62 | 3.65E-02 | 0.914 | 0 | 7.64E-03 | 8.00E-03 |
| rs9852808 | 3 | 46667977 | A | G | 0.92 | 1.57 (1.25-1.98) | 7.36E-05 | 0.095 | 0.095 | 0.59 | 3.38E-02 | 0.932 | 0 | 7.63E-03 | 8.00E-03 |
| rs59157540 | 3 | 46668080 | C | T | 0.92 | 1.57 (1.25-1.98) | 7.39E-05 | 0.095 | 0.095 | 0.59 | 3.37E-02 | 0.932 | 0 | 7.63E-03 | 8.03E-03 |
| rs11919792 | 3 | 46668167 | T | C | 0.92 | 1.57 (1.25-1.98) | 7.39E-05 | 0.096 | 0.096 | 0.59 | 3.36E-02 | 0.932 | 0 | 7.62E-03 | 8.04E-03 |
| rs11915788 | 3 | 46668254 | G | T | 0.92 | 1.57 (1.25-1.98) | 7.42E-05 | 0.087 | 0.087 | 0.62 | 3.62E-02 | 0.915 | 0 | 7.63E-03 | 8.07E-03 |
| rs35165054 | 3 | 46668494 | C | A | 0.92 | 1.57 (1.25-1.98) | 7.41E-05 | 0.097 | 0.097 | 0.59 | 3.34E-02 | 0.933 | 0 | 7.62E-03 | 8.07E-03 |
| rs9820361 | 3 | 46668854 | C | T | 0.92 | 1.57 (1.25-1.98) | 7.43E-05 | 0.088 | 0.088 | 0.62 | 3.58E-02 | 0.916 | 0 | 7.61E-03 | 8.12E-03 |
| rs9820372 | 3 | 46668877 | C | G | 0.92 | 1.57 (1.25-1.98) | 7.44E-05 | 0.097 | 0.097 | 0.59 | 3.31E-02 | 0.935 | 0 | 7.62E-03 | 8.12E-03 |
| rs9820785 | 3 | 46668997 | G | A | 0.92 | 1.57 (1.25-1.98) | 7.44E-05 | 0.088 | 0.088 | 0.62 | 3.57E-02 | 0.917 | 0 | 7.62E-03 | 8.13E-03 |
| rs9820845 | 3 | 46669177 | C | G | 0.92 | 1.57 (1.25-1.98) | 7.46E-05 | 0.098 | 0.098 | 0.59 | 3.29E-02 | 0.936 | 0 | 7.61E-03 | 8.16E-03 |
| rs9820861 | 3 | 46669194 | C | G | 0.92 | 1.57 (1.25-1.98) | 7.45E-05 | 0.097 | 0.097 | 0.58 | 3.33E-02 | 0.935 | 0 | 7.59E-03 | 8.19E-03 |
| rs9821418 | 3 | 46669322 | G | A | 0.92 | 1.57 (1.25-1.98) | 7.42E-05 | 0.098 | 0.098 | 0.59 | 3.27E-02 | 0.936 | 0 | 7.58E-03 | 8.15E-03 |
| rs9841203 | 3 | 46669349 | T | C | 0.92 | 1.57 (1.25-1.98) | 7.46E-05 | 0.099 | 0.099 | 0.59 | 3.27E-02 | 0.936 | 0 | 7.60E-03 | 8.18E-03 |
| rs9841229 | 3 | 46669392 | T | C | 0.92 | 1.57 (1.25-1.98) | 7.45E-05 | 0.099 | 0.099 | 0.6 | 3.26E-02 | 0.936 | 0 | 7.61E-03 | 8.15E-03 |
| rs72906115 | 3 | 46669439 | C | T | 0.92 | 1.57 (1.25-1.98) | 7.48E-05 | 0.09 | 0.09 | 0.62 | 3.51E-02 | 0.919 | 0 | 7.60E-03 | 8.21E-03 |
| rs34727142 | 3 | 46669563 | C | A | 0.92 | 1.57 (1.25-1.98) | 7.52E-05 | 0.099 | 0.099 | 0.59 | 3.26E-02 | 0.936 | 0 | 7.58E-03 | 8.27E-03 |
| rs34671776 | 3 | 46669694 | T | C | 0.92 | 1.57 (1.25-1.98) | 7.51E-05 | 0.1 | 0.1 | 0.6 | 3.24E-02 | 0.937 | 0 | 7.59E-03 | 8.27E-03 |
| rs34256467 | 3 | 46669741 | A | G | 0.92 | 1.57 (1.25-1.98) | 7.53E-05 | 0.1 | 0.1 | 0.59 | 3.23E-02 | 0.938 | 0 | 7.57E-03 | 8.32E-03 |
| rs34326471 | 3 | 46670070 | T | G | 0.92 | 1.57 (1.25-1.98) | 7.51E-05 | 0.091 | 0.091 | 0.62 | 3.45E-02 | 0.921 | 0 | 7.56E-03 | 8.32E-03 |
| rs61621072 | 3 | 46670079 | G | T | 0.92 | 1.57 (1.25-1.98) | 7.52E-05 | 0.105 | 0.105 | 0.6 | 3.07E-02 | 0.944 | 0 | 7.55E-03 | 8.34E-03 |
| rs9864097 | 3 | 46670363 | A | G | 0.92 | 1.57 (1.25-1.98) | 7.53E-05 | 0.101 | 0.101 | 0.6 | 3.18E-02 | 0.94 | 0 | 7.55E-03 | 8.35E-03 |
| rs9868357 | 3 | 46670752 | A | G | 0.92 | 1.58 (1.25-1.98) | 6.56E-05 | 0.088 | 0.088 | 0.57 | 3.31E-02 | 0.92 | 0 | 6.77E-03 | 8.47E-03 |
| rs36174833 | 3 | 46671085 | T | C | 0.92 | 1.57 (1.25-1.97) | 7.57E-05 | 0.102 | 0.102 | 0.6 | 3.15E-02 | 0.942 | 0 | 7.54E-03 | 8.42E-03 |
| rs72906125 | 3 | 46671186 | A | G | 0.92 | 1.57 (1.25-1.97) | 7.77E-05 | 0.106 | 0.106 | 0.6 | 3.10E-02 | 0.947 | 0 | 7.58E-03 | 8.58E-03 |
| rs72906128 | 3 | 46671216 | G | C | 0.92 | 1.57 (1.25-1.97) | 7.57E-05 | 0.105 | 0.105 | 0.59 | 3.10E-02 | 0.945 | 0 | 7.53E-03 | 8.45E-03 |
| rs10510839 | 3 | 60322861 | T | A | 0.56 | 1.24 (1.1-1.39) | 3.17E-04 | 0.322 | 0.349 | 0.18 | 3.68E-04 | 0.041 | 0.2 | 4.97E-03 | 2.52E-02 |
| rs17599845 | 3 | 60327391 | A | G | 0.56 | 1.22 (1.09-1.38) | 6.38E-04 | 0.225 | 0.276 | 0.24 | 4.10E-04 | 5.67E-03 | 0.33 | 1.09E-02 | 2.12E-02 |
| rs6419737 | 3 | 79703548 | G | C | 0.54 | 0.8 (0.7-0.9) | 3.11E-04 | 0.014 | 0.095 | 0.1 | 1.35E-05 | 0.003 | 0.21 | 1.67E-02 | 2.76E-02 |
| rs6775448 | 3 | 79705179 | G | C | 0.54 | 0.79 (0.7-0.9) | 2.83E-04 | 0.011 | 0.085 | 0.1 | 9.50E-06 | 0.002 | 0.21 | 1.45E-02 | 2.75E-02 |
| rs4856228 | 3 | 79705541 | G | A | 0.54 | 0.79 (0.7-0.9) | 2.85E-04 | 0.013 | 0.094 | 0.1 | 1.17E-05 | 0.003 | 0.21 | 1.57E-02 | 2.70E-02 |
| rs4856433 | 3 | 79706059 | A | T | 0.54 | 0.79 (0.7-0.9) | 2.68E-04 | 0.015 | 0.096 | 0.1 | 1.19E-05 | 0.003 | 0.21 | 1.52E-02 | 2.63E-02 |
| rs4856434 | 3 | 79706132 | C | A | 0.54 | 0.79 (0.7-0.9) | 2.77E-04 | 0.013 | 0.093 | 0.1 | 1.13E-05 | 0.003 | 0.21 | 1.54E-02 | 2.69E-02 |
| rs9870711 | 3 | 79706168 | C | A | 0.54 | 0.79 (0.7-0.9) | 2.76E-04 | 0.013 | 0.095 | 0.1 | 1.13E-05 | 0.003 | 0.2 | 1.53E-02 | 2.69E-02 |
| rs4856440 | 3 | 79707004 | G | A | 0.54 | 0.79 (0.7-0.9) | 2.72E-04 | 0.014 | 0.094 | 0.11 | 1.18E-05 | 0.003 | 0.21 | 1.52E-02 | 2.67E-02 |
| rs4856443 | 3 | 79707150 | G | A | 0.54 | 0.79 (0.7-0.9) | 2.69E-04 | 0.014 | 0.093 | 0.11 | 1.14E-05 | 0.003 | 0.21 | 1.51E-02 | 2.66E-02 |
| rs4856444 | 3 | 79707182 | A | G | 0.54 | 0.79 (0.7-0.9) | 2.67E-04 | 0.013 | 0.093 | 0.1 | 1.09E-05 | 0.003 | 0.21 | 1.50E-02 | 2.65E-02 |
| rs4856447 | 3 | 79707357 | T | G | 0.54 | 0.79 (0.7-0.9) | 2.66E-04 | 0.014 | 0.094 | 0.11 | 1.16E-05 | 0.003 | 0.21 | 1.50E-02 | 2.65E-02 |
| rs4856448 | 3 | 79707387 | A | G | 0.54 | 0.79 (0.7-0.9) | 2.65E-04 | 0.013 | 0.091 | 0.11 | 1.06E-05 | 0.002 | 0.21 | 1.50E-02 | 2.65E-02 |
| rs7628280 | 3 | 79707453 | G | C | 0.54 | 0.79 (0.7-0.9) | 2.62E-04 | 0.013 | 0.092 | 0.1 | 1.07E-05 | 0.003 | 0.21 | 1.48E-02 | 2.64E-02 |
| rs7640127 | 3 | 79707605 | A | T | 0.54 | 0.79 (0.7-0.9) | 2.50E-04 | 0.013 | 0.093 | 0.1 | 1.02E-05 | 0.003 | 0.21 | 1.49E-02 | 2.52E-02 |
| rs9880911 | 3 | 79707817 | G | A | 0.54 | 0.79 (0.7-0.9) | 2.58E-04 | 0.014 | 0.093 | 0.11 | 1.12E-05 | 0.002 | 0.21 | 1.46E-02 | 2.63E-02 |
| rs7431260 | 3 | 79708481 | T | A | 0.54 | 0.79 (0.69-0.89) | 1.80E-04 | 0.017 | 0.098 | 0.11 | 9.23E-06 | 0.002 | 0.22 | 1.05E-02 | 2.70E-02 |
| rs6771093 | 3 | 79709304 | A | C | 0.56 | 0.81 (0.72-0.92) | 9.21E-04 | 0.008 | 0.098 | 0.08 | 2.28E-05 | 0.006 | 0.16 | 2.68E-02 | 5.86E-02 |
| rs7428022 | 3 | 79709843 | T | C | 0.54 | 0.79 (0.7-0.89) | 2.05E-04 | 0.014 | 0.092 | 0.11 | 9.02E-06 | 0.002 | 0.22 | 1.24E-02 | 2.48E-02 |
| rs9869899 | 3 | 79712822 | T | C | 0.56 | 0.81 (0.71-0.91) | 6.96E-04 | 0.008 | 0.098 | 0.08 | 1.84E-05 | 0.005 | 0.17 | 2.22E-02 | 5.42E-02 |
| rs9824870 | 3 | 79714522 | C | T | 0.56 | 0.8 (0.71-0.91) | 4.83E-04 | 0.007 | 0.09 | 0.08 | 1.08E-05 | 0.004 | 0.17 | 1.74E-02 | 4.89E-02 |
| rs9825204 | 3 | 79714764 | G | T | 0.56 | 0.8 (0.71-0.91) | 4.34E-04 | 0.007 | 0.087 | 0.09 | 9.55E-06 | 0.003 | 0.17 | 1.61E-02 | 4.78E-02 |
| rs9825870 | 3 | 79715200 | A | T | 0.56 | 0.8 (0.71-0.91) | 4.27E-04 | 0.007 | 0.086 | 0.09 | 9.33E-06 | 0.003 | 0.18 | 1.59E-02 | 4.75E-02 |
| rs9857798 | 3 | 79715860 | G | C | 0.57 | 0.8 (0.71-0.91) | 4.91E-04 | 0.005 | 0.052 | 0.13 | 8.68E-06 | 0.001 | 0.24 | 1.29E-02 | 6.20E-02 |
| rs6770961 | 3 | 79716540 | C | A | 0.57 | 0.8 (0.71-0.9) | 3.23E-04 | 0.003 | 0.063 | 0.08 | 3.78E-06 | 0.002 | 0.16 | 9.59E-03 | 5.29E-02 |
| rs4856253 | 3 | 79719062 | T | C | 0.57 | 0.8 (0.7-0.9) | 2.95E-04 | 0.003 | 0.061 | 0.08 | 3.26E-06 | 0.002 | 0.16 | 8.97E-03 | 5.18E-02 |
| rs9878764 | 3 | 79719636 | T | C | 0.57 | 0.8 (0.7-0.9) | 2.89E-04 | 0.003 | 0.059 | 0.09 | 3.12E-06 | 0.002 | 0.16 | 8.80E-03 | 5.16E-02 |
| rs4856257 | 3 | 79720785 | A | C | 0.57 | 0.8 (0.7-0.9) | 2.78E-04 | 0.003 | 0.057 | 0.09 | 2.88E-06 | 0.002 | 0.16 | 8.56E-03 | 5.11E-02 |
| rs3923148 | 3 | 79727278 | T | A | 0.56 | 0.8 (0.71-0.9) | 2.87E-04 | 0.003 | 0.054 | 0.09 | 3.01E-06 | 0.002 | 0.17 | 1.29E-02 | 4.04E-02 |
| rs6548648 | 3 | 79728730 | G | T | 0.57 | 0.79 (0.7-0.9) | 2.06E-04 | 0.003 | 0.058 | 0.08 | 2.00E-06 | 0.002 | 0.16 | 6.88E-03 | 4.74E-02 |
| rs6762755 | 3 | 79729947 | G | A | 0.57 | 0.79 (0.7-0.9) | 2.00E-04 | 0.003 | 0.059 | 0.08 | 1.96E-06 | 0.002 | 0.16 | 6.70E-03 | 4.71E-02 |
| rs7635296 | 3 | 79730605 | T | C | 0.57 | 0.79 (0.7-0.9) | 1.98E-04 | 0.003 | 0.059 | 0.08 | 1.95E-06 | 0.002 | 0.16 | 6.66E-03 | 4.69E-02 |
| rs7613379 | 3 | 79730821 | C | A | 0.57 | 0.79 (0.7-0.9) | 1.97E-04 | 0.003 | 0.06 | 0.08 | 1.95E-06 | 0.002 | 0.16 | 6.65E-03 | 4.69E-02 |
| rs7635587 | 3 | 79730872 | T | C | 0.57 | 0.79 (0.7-0.9) | 1.98E-04 | 0.003 | 0.06 | 0.08 | 1.96E-06 | 0.002 | 0.16 | 6.65E-03 | 4.68E-02 |
| rs7429100 | 3 | 79737521 | A | T | 0.57 | 0.79 (0.7-0.89) | 1.75E-04 | 0.003 | 0.068 | 0.07 | 1.84E-06 | 0.003 | 0.14 | 5.78E-03 | 4.49E-02 |
| rs12107379 | 3 | 79738224 | A | G | 0.56 | 0.79 (0.7-0.9) | 2.07E-04 | 0.007 | 0.096 | 0.07 | 4.55E-06 | 0.005 | 0.14 | 6.07E-03 | 4.63E-02 |
| rs13060599 | 3 | 79738599 | T | C | 0.56 | 0.79 (0.7-0.9) | 2.09E-04 | 0.009 | 0.116 | 0.05 | 5.73E-06 | 0.007 | 0.12 | 5.80E-03 | 4.89E-02 |
| rs9631514 | 3 | 79740102 | T | A | 0.57 | 0.79 (0.7-0.89) | 1.69E-04 | 0.003 | 0.072 | 0.07 | 1.86E-06 | 0.003 | 0.14 | 5.50E-03 | 4.49E-02 |
| rs10212228 | 3 | 79744923 | C | T | 0.56 | 0.79 (0.7-0.9) | 1.84E-04 | 0.003 | 0.076 | 0.07 | 2.24E-06 | 0.003 | 0.14 | 5.65E-03 | 4.58E-02 |
| rs7431092 | 3 | 79746262 | C | T | 0.57 | 0.79 (0.7-0.89) | 1.60E-04 | 0.004 | 0.079 | 0.07 | 2.07E-06 | 0.003 | 0.14 | 5.05E-03 | 4.50E-02 |
| rs9862551 | 3 | 79746590 | T | C | 0.57 | 0.79 (0.7-0.89) | 1.60E-04 | 0.004 | 0.079 | 0.07 | 2.05E-06 | 0.003 | 0.14 | 5.05E-03 | 4.49E-02 |
| rs9812795 | 3 | 79747727 | G | T | 0.57 | 0.79 (0.7-0.89) | 1.59E-04 | 0.004 | 0.08 | 0.07 | 2.10E-06 | 0.003 | 0.14 | 5.02E-03 | 4.48E-02 |
| rs9873237 | 3 | 79748478 | C | G | 0.57 | 0.79 (0.7-0.89) | 1.59E-04 | 0.004 | 0.081 | 0.07 | 2.16E-06 | 0.003 | 0.14 | 5.00E-03 | 4.51E-02 |
| rs7430339 | 3 | 79766511 | G | A | 0.58 | 0.8 (0.71-0.89) | 1.20E-04 | 0.008 | 0.113 | 0.07 | 3.06E-06 | 0.005 | 0.14 | 2.11E-03 | 5.99E-02 |
| rs4856298 | 3 | 79783137 | A | G | 0.58 | 0.8 (0.72-0.9) | 2.00E-04 | 0.04 | 0.391 | 0.01 | 2.25E-05 | 0.08 | 0.02 | 5.28E-03 | 4.19E-02 |
| rs7426689 | 3 | 79785050 | C | A | 0.55 | 0.81 (0.72-0.91) | 5.21E-04 | 0.031 | 0.453 | 0 | 4.50E-05 | 0.106 | 0.01 | 4.17E-03 | 1.02E-01 |
| rs7430639 | 3 | 79785085 | A | G | 0.59 | 0.81 (0.72-0.9) | 2.21E-04 | 0.03 | 0.417 | 0 | 1.91E-05 | 0.09 | 0.01 | 4.13E-03 | 5.10E-02 |
| rs7431063 | 3 | 79785218 | A | G | 0.59 | 0.81 (0.72-0.9) | 2.40E-04 | 0.031 | 0.42 | 0 | 2.15E-05 | 0.091 | 0.01 | 4.37E-03 | 5.25E-02 |
| rs3924599 | 3 | 79789184 | G | A | 0.59 | 0.81 (0.72-0.91) | 2.60E-04 | 0.032 | 0.426 | 0 | 2.36E-05 | 0.095 | 0.01 | 4.62E-03 | 5.38E-02 |
| rs9309831 | 3 | 79789408 | T | A | 0.59 | 0.81 (0.72-0.91) | 2.64E-04 | 0.031 | 0.424 | 0 | 2.34E-05 | 0.094 | 0.01 | 4.67E-03 | 5.41E-02 |
| rs9309832 | 3 | 79789488 | A | T | 0.61 | 0.8 (0.71-0.9) | 2.02E-04 | 0.077 | 0.545 | 0 | 4.51E-05 | 0.157 | 0.01 | 1.74E-03 | 8.13E-02 |
| rs7426439 | 3 | 79789645 | T | A | 0.57 | 0.8 (0.72-0.91) | 3.06E-04 | 0.026 | 0.426 | 0 | 2.30E-05 | 0.095 | 0.01 | 3.72E-03 | 6.23E-02 |
| rs1995402 | 3 | 79790407 | G | T | 0.59 | 0.81 (0.72-0.91) | 2.70E-04 | 0.031 | 0.425 | 0 | 2.42E-05 | 0.095 | 0.01 | 4.75E-03 | 5.45E-02 |
| rs1995401 | 3 | 79790433 | C | T | 0.59 | 0.81 (0.72-0.91) | 2.72E-04 | 0.031 | 0.425 | 0 | 2.43E-05 | 0.095 | 0.01 | 4.77E-03 | 5.46E-02 |
| rs7649774 | 3 | 79791414 | T | C | 0.59 | 0.81 (0.72-0.91) | 2.79E-04 | 0.033 | 0.429 | 0 | 2.58E-05 | 0.097 | 0.01 | 4.85E-03 | 5.50E-02 |
| rs11706045 | 3 | 133619557 | G | A | 0.94 | 1.65 (1.27-2.16) | 1.40E-04 | 0.418 | 0.418 | 0.58 | 1.34E-02 | 0.582 | 0.03 | 2.50E-02 | 1.45E-03 |
| rs9840646 | 3 | 133621614 | T | G | 0.94 | 1.65 (1.27-2.16) | 1.36E-04 | 0.424 | 0.424 | 0.58 | 1.30E-02 | 0.577 | 0.03 | 2.50E-02 | 1.42E-03 |
| rs114398209 | 3 | 133638227 | G | C | 0.94 | 1.78 (1.36-2.33) | 1.46E-05 | 0.692 | 0.692 | 0.61 | 1.64E-03 | 0.378 | 0.03 | 4.31E-04 | 1.90E-02 |
| rs9820098 | 3 | 133651169 | G | T | 0.95 | 1.81 (1.34-2.46) | 7.48E-05 | 0.824 | 0.824 | 0.61 | 3.59E-03 | 0.307 | 0.07 | 2.41E-02 | 1.57E-03 |
| rs9834244 | 3 | 151422581 | G | A | 0.91 | 1.69 (1.36-2.1) | 8.40E-07 | 0.997 | 0.997 | 0.44 | 6.38E-05 | 0.25 | 0.04 | 8.25E-07 | 1.47E-01 |
| rs13322136 | 3 | 151431173 | G | A | 0.9 | 1.57 (1.28-1.93) | 6.76E-06 | 0.793 | 0.74 | 0.32 | 1.15E-04 | 0.164 | 0.08 | 7.53E-06 | 1.43E-01 |
| rs10222538 | 3 | 151436348 | G | T | 0.9 | 1.57 (1.28-1.92) | 7.76E-06 | 0.808 | 0.742 | 0.3 | 1.30E-04 | 0.172 | 0.08 | 9.87E-06 | 1.35E-01 |
| rs7610178 | 3 | 186191696 | C | T | 0.86 | 0.74 (0.62-0.87) | 2.59E-04 | 0.789 | 0.789 | 0.67 | 8.06E-04 | 0.002 | 0.38 | 6.65E-02 | 3.84E-04 |
| rs7622043 | 3 | 186191909 | T | C | 0.84 | 0.76 (0.65-0.89) | 4.49E-04 | 0.791 | 0.791 | 0.56 | 1.15E-03 | 0.002 | 0.38 | 7.17E-02 | 3.68E-04 |
| rs34775372 | 4 | 4796443 | C | T | 0.92 | 1.69 (1.33-2.13) | 6.39E-06 | 0.292 | 0.292 | 0.62 | 5.45E-03 | 0.643 | 0 | 1.45E-03 | 2.56E-03 |
| rs1532347 | 4 | 15003896 | C | G | 0.94 | 1.75 (1.33-2.32) | 4.81E-05 | 0.826 | 0.877 | 0.11 | 1.87E-03 | 0.556 | 0.02 | 1.29E-02 | 3.19E-03 |
| 4:100960911:AG_A | 4 | 100960911 | R | D | 0.99 | 3.84 (1.82-8.11) | 4.26E-05 | 0.474 | 0.474 | 0.52 | 8.77E-04 | 0.003 | 0.36 | 6.10E-03 | 1.46E-02 |
| rs139320088 | 4 | 100968948 | G | C | 0.99 | 3.93 (1.84-8.37) | 3.63E-05 | 0.398 | 0.398 | 0.53 | 5.77E-04 | 0.001 | 0.44 | 5.84E-03 | 1.47E-02 |
| rs114474719 | 4 | 100974337 | T | C | 0.99 | 4.02 (1.87-8.65) | 3.11E-05 | 0.42 | 0.42 | 0.54 | 5.95E-04 | 0.001 | 0.42 | 5.66E-03 | 1.50E-02 |
| rs139005704 | 4 | 100974971 | C | A | 0.99 | 4.03 (1.87-8.68) | 3.06E-05 | 0.422 | 0.422 | 0.54 | 5.97E-04 | 0.001 | 0.42 | 5.63E-03 | 1.50E-02 |
| rs4863620 | 4 | 139876605 | A | G | 0.63 | 1.29 (1.14-1.45) | 2.06E-05 | 0.11 | 0.11 | 0.38 | 7.61E-06 | 0 | 0.5 | 3.10E-03 | 2.68E-02 |
| 4:163325957:CAT_C | 4 | 163325957 | R | D | 0.91 | 0.66 (0.55-0.81) | 4.59E-05 | 0.358 | 0.358 | 0.93 | 1.91E-03 | 0.703 | 0.04 | 1.64E-01 | 1.05E-04 |
| rs2122494 | 4 | 163331379 | A | G | 0.91 | 0.66 (0.55-0.8) | 3.14E-05 | 0.319 | 0.319 | 0.98 | 1.44E-03 | 0.785 | 0.04 | 7.08E-02 | 2.37E-04 |
| rs35509282 | 4 | 163333405 | T | A | 0.91 | 0.67 (0.55-0.8) | 2.06E-05 | 0.353 | 0.353 | 0.96 | 8.73E-04 | 0.753 | 0.04 | 6.20E-02 | 2.13E-04 |
| rs11736440 | 4 | 163336693 | G | A | 0.91 | 0.66 (0.55-0.8) | 1.41E-05 | 0.323 | 0.323 | 0.94 | 6.86E-04 | 0.796 | 0.04 | 2.80E-02 | 2.26E-04 |
| rs11100443 | 4 | 163337191 | C | T | 0.91 | 0.66 (0.55-0.8) | 1.50E-05 | 0.318 | 0.318 | 0.97 | 7.76E-04 | 0.794 | 0.03 | 2.83E-02 | 2.23E-04 |
| 4:163338255:GC_G | 4 | 163338255 | R | D | 0.91 | 0.66 (0.55-0.8) | 1.50E-05 | 0.305 | 0.305 | 0.97 | 8.08E-04 | 0.811 | 0.03 | 2.83E-02 | 2.27E-04 |
| rs9998942 | 4 | 163340404 | C | T | 0.91 | 0.66 (0.55-0.8) | 1.45E-05 | 0.269 | 0.269 | 0.93 | 8.75E-04 | 0.863 | 0.03 | 3.13E-02 | 2.29E-04 |
| rs57336275 | 4 | 163341215 | T | C | 0.91 | 0.66 (0.55-0.8) | 1.50E-05 | 0.268 | 0.268 | 0.93 | 9.00E-04 | 0.865 | 0.03 | 3.18E-02 | 2.34E-04 |
| rs253937 | 5 | 31655104 | A | C | 0.89 | 1.51 (1.24-1.83) | 2.19E-05 | 0.05 | 0.407 | 0.02 | 4.80E-06 | 0.06 | 0.05 | 1.87E-02 | 1.46E-03 |
| rs535910 | 5 | 140273168 | T | G | 0.97 | 0.48 (0.35-0.67) | 1.84E-05 | 0.198 | 0.198 | 0.66 | 1.21E-05 | 0 | 0.62 | 1.60E-02 | 2.46E-04 |
| rs115893558 | 5 | 140274728 | A | C | 0.97 | 0.48 (0.35-0.67) | 1.72E-05 | 0.163 | 0.163 | 0.44 | 8.85E-06 | 0 | 0.49 | 1.51E-02 | 2.43E-04 |
| rs265315 | 5 | 140279735 | C | T | 0.97 | 0.49 (0.35-0.68) | 2.15E-05 | 0.205 | 0.205 | 0.64 | 1.44E-05 | 0 | 0.61 | 1.29E-02 | 3.42E-04 |
| rs169087 | 5 | 140283860 | C | A | 0.97 | 0.48 (0.34-0.67) | 1.35E-05 | 0.202 | 0.202 | 0.64 | 9.44E-06 | 0 | 0.59 | 1.23E-02 | 2.30E-04 |
| rs265313 | 5 | 140285449 | A | C | 0.97 | 0.49 (0.35-0.68) | 1.92E-05 | 0.219 | 0.219 | 0.63 | 1.43E-05 | 0 | 0.58 | 1.90E-02 | 2.26E-04 |
| rs149646315 | 5 | 140288075 | G | T | 0.97 | 0.49 (0.35-0.68) | 1.90E-05 | 0.187 | 0.187 | 0.42 | 1.14E-05 | 0 | 0.46 | 1.91E-02 | 2.23E-04 |
| rs6887800 | 5 | 140300617 | T | A | 0.97 | 0.48 (0.35-0.67) | 1.75E-05 | 0.199 | 0.199 | 0.6 | 1.14E-05 | 0 | 0.58 | 1.96E-02 | 2.05E-04 |
| 5:140305508:C_CT | 5 | 140305508 | R | I | 0.97 | 0.48 (0.35-0.67) | 1.73E-05 | 0.218 | 0.218 | 0.59 | 1.27E-05 | 0 | 0.56 | 1.94E-02 | 2.04E-04 |
| 5:140305520:CTCTT | 5 | 140305520 | R | D | 0.97 | 0.48 (0.35-0.67) | 1.73E-05 | 0.212 | 0.212 | 0.54 | 1.22E-05 | 0 | 0.53 | 1.94E-02 | 2.04E-04 |
| rs74597681 | 5 | 140307002 | G | A | 0.97 | 0.48 (0.35-0.67) | 1.72E-05 | 0.189 | 0.189 | 0.54 | 1.05E-05 | 0 | 0.55 | 1.92E-02 | 2.04E-04 |
| rs155822 | 5 | 140310375 | A | G | 0.97 | 0.48 (0.35-0.67) | 1.71E-05 | 0.239 | 0.239 | 0.6 | 1.40E-05 | 0 | 0.55 | 1.91E-02 | 2.03E-04 |
| rs140426253 | 5 | 140322033 | A | C | 0.97 | 0.49 (0.35-0.68) | 2.55E-05 | 0.234 | 0.234 | 0.65 | 1.88E-05 | 0 | 0.62 | 2.77E-02 | 1.41E-04 |
| rs145543790 | 5 | 140325300 | A | G | 0.97 | 0.49 (0.35-0.68) | 2.57E-05 | 0.233 | 0.233 | 0.65 | 1.89E-05 | 0 | 0.62 | 2.81E-02 | 1.38E-04 |
| rs116397497 | 5 | 140326900 | C | T | 0.97 | 0.49 (0.35-0.68) | 2.58E-05 | 0.231 | 0.231 | 0.66 | 1.87E-05 | 0 | 0.63 | 2.83E-02 | 1.37E-04 |
| rs77655739 | 5 | 140327357 | G | A | 0.97 | 0.49 (0.35-0.68) | 2.59E-05 | 0.231 | 0.231 | 0.66 | 1.88E-05 | 0 | 0.63 | 2.83E-02 | 1.38E-04 |
| rs116815984 | 5 | 140329269 | C | T | 0.97 | 0.49 (0.35-0.68) | 2.61E-05 | 0.231 | 0.231 | 0.67 | 1.89E-05 | 0 | 0.63 | 2.87E-02 | 1.37E-04 |
| rs74485628 | 5 | 140332880 | C | T | 0.97 | 0.49 (0.35-0.68) | 2.63E-05 | 0.229 | 0.229 | 0.68 | 1.88E-05 | 0 | 0.64 | 2.91E-02 | 1.34E-04 |
| rs145658065 | 5 | 140333932 | G | A | 0.97 | 0.49 (0.35-0.68) | 2.64E-05 | 0.228 | 0.228 | 0.68 | 1.87E-05 | 0 | 0.64 | 2.93E-02 | 1.34E-04 |
| rs76093065 | 5 | 140337098 | T | C | 0.97 | 0.49 (0.35-0.68) | 2.68E-05 | 0.227 | 0.227 | 0.69 | 1.88E-05 | 0 | 0.65 | 2.97E-02 | 1.33E-04 |
| rs150796442 | 5 | 140338674 | C | T | 0.97 | 0.49 (0.35-0.68) | 2.68E-05 | 0.226 | 0.226 | 0.69 | 1.87E-05 | 0 | 0.65 | 2.98E-02 | 1.33E-04 |
| rs78198535 | 5 | 140339541 | G | A | 0.97 | 0.49 (0.35-0.69) | 2.70E-05 | 0.226 | 0.226 | 0.69 | 1.88E-05 | 0 | 0.65 | 2.99E-02 | 1.33E-04 |
| rs6891232 | 5 | 140341087 | T | C | 0.97 | 0.49 (0.35-0.69) | 2.69E-05 | 0.226 | 0.226 | 0.69 | 1.87E-05 | 0 | 0.65 | 2.99E-02 | 1.32E-04 |
| rs78940637 | 5 | 140341935 | C | A | 0.97 | 0.49 (0.35-0.69) | 2.70E-05 | 0.226 | 0.226 | 0.69 | 1.88E-05 | 0 | 0.65 | 3.00E-02 | 1.32E-04 |
| rs80298031 | 5 | 140341939 | A | G | 0.97 | 0.49 (0.35-0.69) | 2.70E-05 | 0.226 | 0.226 | 0.69 | 1.88E-05 | 0 | 0.65 | 2.99E-02 | 1.32E-04 |
| rs9476499 | 6 | 14103953 | T | C | 0.96 | 1.92 (1.39-2.66) | 4.06E-05 | 0.084 | 0.084 | 0.79 | 3.82E-02 | 0.837 | 0 | 1.08E-02 | 1.24E-02 |
| rs71564305 | 6 | 14108031 | G | A | 0.96 | 2.31 (1.58-3.37) | 3.21E-06 | 0.571 | 0.571 | 0.41 | 9.65E-04 | 0.735 | 0.02 | 3.70E-03 | 7.30E-03 |
| 6:14108558:GAAGA | 6 | 14108558 | R | D | 0.95 | 1.96 (1.43-2.7) | 1.20E-05 | 0.22 | 0.22 | 0.69 | 8.91E-03 | 0.987 | 0.01 | 3.16E-03 | 9.31E-03 |
| 6:66887242:GTTAC | 6 | 66887242 | R | D | 0.99 | 0.34 (0.2-0.57) | 3.56E-05 | 0.245 | 0.546 | 0.17 | 7.24E-05 | 0.069 | 0.12 | 3.73E-01 | 8.76E-07 |
| rs112250666 | 6 | 66891721 | C | T | 0.99 | 0.33 (0.19-0.56) | 2.86E-05 | 0.245 | 0.536 | 0.18 | 6.49E-05 | 0.068 | 0.12 | 3.77E-01 | 8.86E-07 |
| rs112676223 | 6 | 66898285 | C | T | 0.99 | 0.31 (0.18-0.53) | 1.64E-05 | 0.243 | 0.536 | 0.18 | 4.96E-05 | 0.074 | 0.1 | 3.84E-01 | 9.22E-07 |
| rs113836409 | 6 | 66901267 | A | C | 0.99 | 0.26 (0.14-0.47) | 2.81E-06 | 0.236 | 0.541 | 0.17 | 2.40E-05 | 0.092 | 0.06 | 4.96E-01 | 1.15E-06 |
| rs113213396 | 6 | 66901759 | A | G | 0.99 | 0.25 (0.14-0.46) | 2.24E-06 | 0.236 | 0.541 | 0.17 | 2.22E-05 | 0.094 | 0.06 | 5.71E-01 | 1.19E-06 |
| rs79699040 | 6 | 66902665 | A | T | 0.99 | 0.25 (0.14-0.46) | 2.17E-06 | 0.236 | 0.542 | 0.17 | 2.19E-05 | 0.095 | 0.06 | 5.88E-01 | 1.20E-06 |
| rs112770610 | 6 | 66903303 | T | C | 0.99 | 0.25 (0.14-0.46) | 2.13E-06 | 0.236 | 0.542 | 0.17 | 2.17E-05 | 0.095 | 0.06 | 5.98E-01 | 1.20E-06 |
| rs112477258 | 6 | 66904699 | A | C | 0.99 | 0.25 (0.14-0.45) | 1.95E-06 | 0.234 | 0.541 | 0.18 | 2.05E-05 | 0.094 | 0.06 | 6.68E-01 | 1.22E-06 |
| rs111694744 | 6 | 66905848 | A | T | 0.99 | 0.25 (0.14-0.45) | 1.75E-06 | 0.252 | 0.586 | 0.16 | 2.17E-05 | 0.111 | 0.05 | 7.31E-01 | 1.46E-06 |
| rs112319963 | 6 | 66907171 | T | C | 0.99 | 0.25 (0.13-0.45) | 1.53E-06 | 0.233 | 0.546 | 0.17 | 1.81E-05 | 0.097 | 0.05 | 6.23E-01 | 1.33E-06 |
| rs112796103 | 6 | 66913441 | C | T | 0.99 | 0.24 (0.13-0.45) | 1.59E-06 | 0.237 | 0.567 | 0.16 | 1.89E-05 | 0.103 | 0.05 | 6.19E-01 | 1.38E-06 |
| rs113091378 | 6 | 66918814 | C | T | 0.99 | 0.24 (0.13-0.45) | 1.65E-06 | 0.231 | 0.551 | 0.17 | 1.84E-05 | 0.097 | 0.06 | 6.13E-01 | 1.43E-06 |
| rs111342653 | 6 | 66929163 | G | A | 0.99 | 0.24 (0.13-0.45) | 1.75E-06 | 0.234 | 0.577 | 0.16 | 1.91E-05 | 0.104 | 0.05 | 5.96E-01 | 1.51E-06 |
| rs79682182 | 6 | 66934687 | T | A | 0.99 | 0.24 (0.13-0.45) | 1.81E-06 | 0.233 | 0.584 | 0.16 | 1.94E-05 | 0.105 | 0.05 | 5.73E-01 | 1.58E-06 |
| rs112388593 | 6 | 66936022 | A | G | 0.99 | 0.24 (0.13-0.45) | 1.82E-06 | 0.226 | 0.556 | 0.17 | 1.84E-05 | 0.096 | 0.06 | 5.58E-01 | 1.59E-06 |
| rs113410483 | 6 | 66936093 | T | C | 0.99 | 0.24 (0.13-0.45) | 1.82E-06 | 0.226 | 0.556 | 0.17 | 1.84E-05 | 0.096 | 0.06 | 5.54E-01 | 1.59E-06 |
| rs188823759 | 6 | 66936430 | A | T | 0.99 | 0.24 (0.13-0.45) | 1.81E-06 | 0.224 | 0.548 | 0.17 | 1.82E-05 | 0.093 | 0.06 | 5.48E-01 | 1.59E-06 |
| rs111608927 | 6 | 66940238 | C | T | 0.99 | 0.24 (0.13-0.45) | 1.85E-06 | 0.232 | 0.585 | 0.16 | 1.94E-05 | 0.105 | 0.05 | 5.54E-01 | 1.62E-06 |
| rs113787803 | 6 | 66957788 | T | C | 0.99 | 0.24 (0.13-0.45) | 2.14E-06 | 0.221 | 0.576 | 0.16 | 1.89E-05 | 0.097 | 0.06 | 5.70E-01 | 1.84E-06 |
| rs142539400 | 6 | 66959151 | C | A | 0.99 | 0.24 (0.13-0.45) | 2.16E-06 | 0.226 | 0.593 | 0.16 | 1.97E-05 | 0.103 | 0.06 | 5.74E-01 | 1.85E-06 |
| rs111516034 | 6 | 66976771 | G | T | 0.99 | 0.24 (0.13-0.45) | 2.19E-06 | 0.209 | 0.58 | 0.16 | 1.72E-05 | 0.094 | 0.06 | 5.63E-01 | 1.88E-06 |
| rs111428992 | 6 | 66996495 | G | C | 0.99 | 0.24 (0.13-0.44) | 2.24E-06 | 0.2 | 0.582 | 0.16 | 1.57E-05 | 0.09 | 0.07 | 5.75E-01 | 1.92E-06 |
| rs112715220 | 6 | 66996702 | C | T | 0.99 | 0.24 (0.13-0.44) | 2.23E-06 | 0.199 | 0.581 | 0.16 | 1.55E-05 | 0.089 | 0.07 | 5.78E-01 | 1.91E-06 |
| rs112103904 | 6 | 66997327 | T | C | 0.99 | 0.24 (0.13-0.44) | 2.24E-06 | 0.198 | 0.581 | 0.16 | 1.52E-05 | 0.088 | 0.07 | 5.84E-01 | 1.90E-06 |
| rs113019479 | 6 | 66997935 | T | G | 0.99 | 0.24 (0.13-0.44) | 2.24E-06 | 0.195 | 0.579 | 0.16 | 1.47E-05 | 0.086 | 0.07 | 5.91E-01 | 1.90E-06 |
| rs113493780 | 6 | 66998358 | G | A | 0.99 | 0.24 (0.13-0.44) | 2.24E-06 | 0.195 | 0.584 | 0.16 | 1.47E-05 | 0.087 | 0.07 | 5.96E-01 | 1.90E-06 |
| rs113371349 | 6 | 66998952 | A | T | 0.99 | 0.24 (0.13-0.44) | 2.23E-06 | 0.191 | 0.575 | 0.16 | 1.39E-05 | 0.083 | 0.07 | 5.95E-01 | 1.89E-06 |
| rs111797154 | 6 | 66999117 | C | T | 0.99 | 0.24 (0.13-0.44) | 2.23E-06 | 0.19 | 0.575 | 0.16 | 1.37E-05 | 0.082 | 0.07 | 5.88E-01 | 1.90E-06 |
| rs112589797 | 6 | 66999374 | C | T | 0.99 | 0.24 (0.13-0.44) | 2.22E-06 | 0.189 | 0.575 | 0.16 | 1.36E-05 | 0.082 | 0.07 | 5.86E-01 | 1.89E-06 |
| rs111732502 | 6 | 66999522 | G | A | 0.99 | 0.23 (0.13-0.44) | 2.23E-06 | 0.189 | 0.574 | 0.16 | 1.38E-05 | 0.082 | 0.07 | 5.95E-01 | 1.89E-06 |
| rs113163746 | 6 | 66999794 | C | T | 0.99 | 0.23 (0.13-0.44) | 2.17E-06 | 0.19 | 0.577 | 0.16 | 1.35E-05 | 0.082 | 0.07 | 8.60E-01 | 1.72E-06 |
| rs73454471 | 6 | 67000181 | T | A | 0.99 | 0.23 (0.12-0.44) | 2.22E-06 | 0.203 | 0.601 | 0.16 | 1.50E-05 | 0.091 | 0.07 | 5.81E-01 | 1.89E-06 |
| rs72915109 | 6 | 92335740 | A | G | 0.91 | 0.66 (0.54-0.81) | 7.20E-05 | 0.844 | 0.94 | 0.04 | 1.12E-03 | 0.571 | 0.01 | 1.35E-02 | 1.61E-02 |
| rs235701 | 6 | 124247963 | C | T | 0.81 | 0.75 (0.65-0.88) | 2.79E-04 | 0.638 | 0.781 | 0.28 | 8.74E-04 | 0.15 | 0.17 | 1.02E-03 | 3.62E-01 |
| rs9347489 | 6 | 161718532 | A | G | 0.57 | 0.79 (0.7-0.9) | 2.06E-04 | 0.366 | 0.995 | 0.02 | 3.14E-04 | 0.406 | 0.03 | 7.26E-04 | 3.43E-02 |
| rs6902135 | 6 | 166710938 | A | G | 0.55 | 1.32 (1.16-1.49) | 1.72E-05 | 0.992 | 0.992 | 0.76 | 2.11E-04 | 0.181 | 0.17 | 8.55E-05 | 1.02E-01 |
| rs9348117 | 6 | 166720614 | T | C | 0.51 | 1.29 (1.15-1.45) | 7.98E-06 | 0.598 | 0.598 | 0.58 | 3.64E-04 | 0.599 | 0.04 | 5.36E-05 | 7.08E-02 |
| rs13205770 | 6 | 166721424 | C | T | 0.51 | 1.3 (1.15-1.46) | 1.41E-05 | 0.732 | 0.732 | 0.8 | 3.24E-04 | 0.389 | 0.1 | 1.01E-04 | 8.44E-02 |
| rs4075452 | 6 | 166722417 | T | C | 0.51 | 1.3 (1.16-1.45) | 4.67E-06 | 0.669 | 0.669 | 0.52 | 2.99E-04 | 0.588 | 0.03 | 5.77E-05 | 3.69E-02 |
| rs4075453 | 6 | 166722467 | C | T | 0.51 | 1.3 (1.16-1.45) | 4.65E-06 | 0.67 | 0.67 | 0.52 | 2.95E-04 | 0.587 | 0.03 | 5.75E-05 | 3.69E-02 |
| rs4075454 | 6 | 166722486 | T | C | 0.51 | 1.3 (1.16-1.45) | 4.64E-06 | 0.669 | 0.669 | 0.52 | 2.95E-04 | 0.587 | 0.03 | 5.73E-05 | 3.69E-02 |
| rs60636526 | 6 | 166727450 | G | A | 0.63 | 1.27 (1.13-1.43) | 7.75E-05 | 0.424 | 0.424 | 0.98 | 2.37E-03 | 0.642 | 0.08 | 4.36E-04 | 5.49E-02 |
| rs4318852 | 6 | 166728853 | G | A | 0.64 | 1.26 (1.12-1.42) | 1.49E-04 | 0.327 | 0.327 | 0.99 | 4.79E-03 | 0.759 | 0.06 | 3.66E-04 | 8.83E-02 |
| rs7986 | 7 | 36552656 | G | A | 0.63 | 1.23 (1.09-1.39) | 5.01E-04 | 0.258 | 0.258 | 0.61 | 1.36E-02 | 0.934 | 0.04 | 2.56E-03 | 1.00E-01 |
| rs7778570 | 7 | 38749008 | A | C | 0.8 | 0.7 (0.61-0.81) | 1.31E-06 | 0.245 | 0.245 | 0.42 | 2.34E-06 | 0.005 | 0.23 | 2.25E-04 | 2.48E-02 |
| rs7779329 | 7 | 38749544 | A | G | 0.8 | 0.7 (0.61-0.81) | 1.30E-06 | 0.246 | 0.246 | 0.42 | 2.34E-06 | 0.005 | 0.23 | 2.26E-04 | 2.46E-02 |
| rs1978172 | 7 | 38749836 | A | T | 0.8 | 0.7 (0.61-0.81) | 1.29E-06 | 0.246 | 0.246 | 0.42 | 2.33E-06 | 0.005 | 0.23 | 2.26E-04 | 2.44E-02 |
| rs1978171 | 7 | 38750012 | C | T | 0.81 | 0.71 (0.61-0.81) | 1.78E-06 | 0.222 | 0.222 | 0.43 | 2.60E-06 | 0.003 | 0.27 | 3.04E-04 | 2.44E-02 |
| rs17700072 | 7 | 38750986 | G | C | 0.8 | 0.7 (0.61-0.81) | 1.30E-06 | 0.244 | 0.244 | 0.48 | 2.30E-06 | 0.003 | 0.26 | 1.95E-04 | 3.05E-02 |
| rs7794030 | 7 | 38752094 | A | G | 0.81 | 0.7 (0.61-0.81) | 1.07E-06 | 0.268 | 0.268 | 0.47 | 2.21E-06 | 0.005 | 0.24 | 2.13E-04 | 2.64E-02 |
| rs35791079 | 7 | 38752153 | G | A | 0.8 | 0.7 (0.61-0.81) | 1.25E-06 | 0.254 | 0.254 | 0.46 | 2.37E-06 | 0.004 | 0.25 | 2.00E-04 | 2.92E-02 |
| rs1859494 | 7 | 38753322 | T | A | 0.8 | 0.7 (0.61-0.81) | 1.21E-06 | 0.255 | 0.255 | 0.46 | 2.32E-06 | 0.005 | 0.24 | 2.04E-04 | 2.82E-02 |
| rs7783546 | 7 | 38756960 | G | T | 0.81 | 0.7 (0.6-0.81) | 1.47E-06 | 0.4 | 0.405 | 0.27 | 5.64E-06 | 0.052 | 0.11 | 3.25E-04 | 1.49E-02 |
| rs7797871 | 7 | 38758326 | A | G | 0.73 | 0.77 (0.68-0.87) | 3.86E-05 | 0.725 | 0.725 | 0.66 | 2.21E-04 | 0.05 | 0.23 | 5.19E-02 | 2.35E-03 |
| rs12701659 | 7 | 38758903 | T | C | 0.8 | 0.71 (0.62-0.82) | 2.35E-06 | 0.161 | 0.172 | 0.34 | 2.03E-06 | 0.002 | 0.27 | 3.71E-04 | 3.34E-02 |
| rs35253728 | 7 | 38758963 | A | G | 0.8 | 0.71 (0.62-0.82) | 2.38E-06 | 0.161 | 0.173 | 0.34 | 2.06E-06 | 0.002 | 0.27 | 3.74E-04 | 3.38E-02 |
| rs66796288 | 7 | 38759534 | C | T | 0.8 | 0.71 (0.62-0.82) | 2.40E-06 | 0.16 | 0.174 | 0.33 | 2.06E-06 | 0.002 | 0.27 | 3.74E-04 | 3.39E-02 |
| rs17767770 | 7 | 38763577 | A | T | 0.8 | 0.72 (0.62-0.82) | 3.50E-06 | 0.198 | 0.201 | 0.36 | 3.74E-06 | 0.002 | 0.27 | 4.32E-04 | 4.41E-02 |
| rs17700172 | 7 | 38764586 | T | C | 0.8 | 0.72 (0.62-0.82) | 3.40E-06 | 0.156 | 0.182 | 0.3 | 2.64E-06 | 0.002 | 0.26 | 3.93E-04 | 4.65E-02 |
| rs3735347 | 7 | 38765527 | A | C | 0.8 | 0.72 (0.62-0.83) | 4.60E-06 | 0.158 | 0.183 | 0.3 | 3.46E-06 | 0.002 | 0.27 | 3.93E-04 | 5.14E-02 |
| rs1468348 | 7 | 38767914 | T | C | 0.8 | 0.72 (0.62-0.83) | 4.72E-06 | 0.172 | 0.204 | 0.28 | 3.99E-06 | 0.004 | 0.25 | 4.68E-04 | 4.45E-02 |
| rs34263795 | 7 | 38772671 | G | A | 0.95 | 0.58 (0.44-0.75) | 7.00E-05 | 0.217 | 0.26 | 0.21 | 5.92E-05 | 0.015 | 0.22 | 7.81E-05 | 6.81E-01 |
| rs4433098 | 7 | 52284370 | A | G | 0.58 | 1.31 (1.15-1.48) | 2.53E-05 | 0.278 | 0.592 | 0.2 | 4.61E-05 | 0.065 | 0.15 | 1.41E-03 | 3.78E-02 |
| rs12672974 | 7 | 52285911 | A | G | 0.58 | 1.31 (1.15-1.48) | 2.61E-05 | 0.275 | 0.59 | 0.2 | 4.61E-05 | 0.064 | 0.15 | 3.90E-02 | 1.40E-03 |
| rs12673016 | 7 | 52286230 | A | G | 0.58 | 1.31 (1.15-1.48) | 2.61E-05 | 0.272 | 0.578 | 0.2 | 4.58E-05 | 0.061 | 0.15 | 3.92E-02 | 1.40E-03 |
| rs4523204 | 7 | 52287973 | A | G | 0.58 | 1.31 (1.15-1.48) | 2.61E-05 | 0.275 | 0.592 | 0.19 | 4.70E-05 | 0.066 | 0.15 | 1.38E-03 | 3.94E-02 |
| rs60882649 | 7 | 52291173 | A | T | 0.56 | 1.31 (1.15-1.48) | 3.58E-05 | 0.327 | 0.632 | 0.21 | 8.02E-05 | 0.082 | 0.14 | 6.31E-02 | 1.08E-03 |
| rs11238188 | 7 | 52293518 | A | T | 0.58 | 1.31 (1.15-1.48) | 2.75E-05 | 0.276 | 0.635 | 0.17 | 5.07E-05 | 0.082 | 0.13 | 1.53E-03 | 3.95E-02 |
| rs10899744 | 7 | 52295325 | G | A | 0.58 | 1.31 (1.15-1.48) | 2.79E-05 | 0.276 | 0.64 | 0.17 | 5.14E-05 | 0.084 | 0.13 | 1.52E-03 | 4.00E-02 |
| rs4947405 | 7 | 52298707 | T | C | 0.58 | 1.3 (1.15-1.48) | 4.03E-05 | 0.315 | 0.65 | 0.19 | 8.42E-05 | 0.089 | 0.13 | 2.05E-03 | 4.28E-02 |
| rs4317525 | 7 | 52303988 | A | C | 0.58 | 1.3 (1.15-1.48) | 3.05E-05 | 0.258 | 0.598 | 0.18 | 4.95E-05 | 0.066 | 0.15 | 1.47E-03 | 4.38E-02 |
| rs75053163 | 7 | 52307141 | I | R | 0.56 | 1.3 (1.15-1.48) | 4.03E-05 | 0.295 | 0.59 | 0.22 | 7.62E-05 | 0.064 | 0.16 | 6.76E-02 | 1.21E-03 |
| rs59386953 | 7 | 52311538 | C | T | 0.61 | 1.29 (1.14-1.47) | 5.38E-05 | 0.515 | 0.515 | 0.53 | 2.30E-04 | 0.037 | 0.24 | 1.38E-03 | 7.52E-02 |
| rs6963554 | 7 | 52316977 | T | C | 0.58 | 1.3 (1.14-1.47) | 3.82E-05 | 0.252 | 0.538 | 0.21 | 5.51E-05 | 0.044 | 0.18 | 5.19E-02 | 1.31E-03 |
| rs62463161 | 7 | 52320600 | G | A | 0.58 | 1.29 (1.14-1.46) | 4.13E-05 | 0.287 | 0.63 | 0.18 | 7.01E-05 | 0.078 | 0.14 | 5.54E-02 | 1.31E-03 |
| rs296313 | 7 | 52435408 | G | A | 0.71 | 0.77 (0.68-0.88) | 1.79E-04 | 0.103 | 0.103 | 0.71 | 5.53E-05 | 5.53E-05 | 0.77 | 2.13E-03 | 1.18E-02 |
| rs11772792 | 7 | 52481483 | A | G | 0.59 | 0.78 (0.7-0.88) | 5.40E-05 | 0.385 | 0.385 | 0.45 | 1.12E-04 | 0.008 | 0.3 | 4.49E-04 | 3.63E-02 |
| rs11981322 | 7 | 52485878 | T | C | 0.57 | 0.78 (0.69-0.88) | 4.20E-05 | 0.444 | 0.444 | 0.64 | 1.24E-04 | 0.004 | 0.33 | 3.15E-04 | 4.50E-02 |
| rs59191429 | 7 | 137663901 | G | A | 0.93 | 1.62 (1.27-2.06) | 5.37E-05 | 0.988 | 0.843 | 0.07 | 4.56E-04 | 0.525 | 0.04 | 5.43E-05 | 3.96E-01 |
| rs34793944 | 8 | 17024449 | A | G | 0.67 | 0.79 (0.7-0.9) | 2.89E-04 | 0.752 | 0.752 | 0.52 | 1.34E-03 | 0.079 | 0.24 | 1.73E-03 | 5.37E-02 |
| rs13267854 | 8 | 17025769 | C | T | 0.67 | 0.79 (0.7-0.9) | 3.94E-04 | 0.963 | 0.963 | 0.44 | 2.79E-03 | 0.235 | 0.15 | 1.45E-03 | 7.21E-02 |
| rs6996680 | 8 | 32844577 | A | G | 0.63 | 1.3 (1.15-1.47) | 2.25E-05 | 0.171 | 0.23 | 0.1 | 1.66E-05 | 0.028 | 0.14 | 1.33E-03 | 1.11E-02 |
| rs62499673 | 8 | 32852624 | A | G | 0.57 | 1.25 (1.11-1.41) | 1.56E-04 | 0.133 | 0.198 | 0.06 | 6.53E-05 | 0.04 | 0.1 | 5.20E-03 | 1.13E-02 |
| rs7461712 | 8 | 75405213 | C | T | 0.62 | 0.78 (0.7-0.88) | 3.77E-05 | 0.194 | 0.194 | 0.65 | 3.16E-05 | 0 | 0.54 | 1.08E-02 | 8.35E-03 |
| rs10955309 | 8 | 104141937 | G | A | 0.82 | 0.75 (0.65-0.87) | 1.93E-04 | 0.972 | 0.972 | 0.97 | 1.41E-03 | 0.072 | 0.28 | 1.10E-05 | 6.91E-01 |
| rs28484802 | 8 | 104142530 | C | A | 0.82 | 0.75 (0.65-0.87) | 1.95E-04 | 0.952 | 0.952 | 0.97 | 1.48E-03 | 0.084 | 0.27 | 1.12E-05 | 6.83E-01 |
| rs2935579 | 8 | 104157531 | G | A | 0.76 | 1.3 (1.13-1.49) | 1.64E-04 | 0.822 | 0.822 | 0.76 | 2.84E-03 | 0.279 | 0.13 | 5.25E-03 | 3.56E-02 |
| rs12548629 | 8 | 104201401 | C | T | 0.73 | 1.41 (1.24-1.61) | 2.54E-07 | 0.537 | 0.772 | 0.25 | 1.02E-04 | 0.522 | 0 | 8.52E-05 | 1.49E-03 |
| rs13279226 | 8 | 104202425 | G | A | 0.73 | 1.4 (1.22-1.6) | 5.44E-07 | 0.535 | 0.771 | 0.25 | 1.68E-04 | 0.524 | 0 | 1.03E-04 | 2.28E-03 |
| rs7002269 | 8 | 104218443 | T | C | 0.73 | 1.39 (1.22-1.59) | 8.52E-07 | 0.618 | 0.806 | 0.26 | 1.72E-04 | 0.502 | 0.01 | 9.48E-05 | 3.68E-03 |
| rs17799604 | 8 | 104229824 | C | T | 0.85 | 1.42 (1.2-1.67) | 3.87E-05 | 0.652 | 0.652 | 0.75 | 1.99E-03 | 0.508 | 0.06 | 4.16E-02 | 1.91E-04 |
| rs17205332 | 8 | 104232037 | T | C | 0.89 | 1.45 (1.2-1.75) | 8.94E-05 | 0.489 | 0.489 | 0.62 | 5.58E-03 | 0.585 | 0.04 | 5.99E-03 | 5.01E-03 |
| rs4734693 | 8 | 104238747 | T | C | 0.64 | 1.26 (1.12-1.42) | 1.22E-04 | 0.23 | 0.23 | 0.74 | 1.20E-02 | 0.922 | 0.02 | 8.12E-03 | 1.58E-03 |
| rs17205590 | 8 | 104243414 | C | T | 0.96 | 2.05 (1.45-2.89) | 1.79E-05 | 0.496 | 0.496 | 0.66 | 2.54E-03 | 0.555 | 0.03 | 9.77E-03 | 1.58E-03 |
| rs12543038 | 8 | 104244309 | T | C | 0.89 | 1.47 (1.21-1.78) | 5.77E-05 | 0.529 | 0.529 | 0.63 | 3.94E-03 | 0.553 | 0.04 | 5.84E-03 | 3.80E-03 |
| rs12541167 | 8 | 104245670 | G | A | 0.89 | 1.47 (1.21-1.78) | 5.76E-05 | 0.53 | 0.53 | 0.62 | 3.93E-03 | 0.552 | 0.04 | 5.78E-03 | 3.82E-03 |
| rs13275377 | 8 | 104253034 | A | G | 0.89 | 1.47 (1.21-1.78) | 5.70E-05 | 0.533 | 0.533 | 0.62 | 3.89E-03 | 0.549 | 0.04 | 5.42E-03 | 4.05E-03 |
| rs4734695 | 8 | 104256725 | T | C | 0.64 | 1.27 (1.12-1.43) | 1.04E-04 | 0.209 | 0.209 | 0.71 | 1.18E-02 | 0.954 | 0.01 | 7.16E-03 | 1.60E-03 |
| rs1982892 | 8 | 104261724 | G | A | 0.89 | 1.47 (1.21-1.79) | 7.06E-05 | 0.513 | 0.513 | 0.65 | 4.85E-03 | 0.566 | 0.04 | 5.44E-03 | 4.71E-03 |
| rs1982893 | 8 | 104261731 | T | C | 0.89 | 1.47 (1.21-1.79) | 7.06E-05 | 0.513 | 0.513 | 0.65 | 4.85E-03 | 0.566 | 0.04 | 5.44E-03 | 4.71E-03 |
| rs1494278 | 8 | 104263332 | A | T | 0.89 | 1.47 (1.21-1.79) | 7.09E-05 | 0.517 | 0.517 | 0.65 | 4.81E-03 | 0.561 | 0.04 | 5.43E-03 | 4.71E-03 |
| rs34201011 | 8 | 104264620 | C | T | 0.89 | 1.46 (1.21-1.77) | 6.38E-05 | 0.638 | 0.638 | 0.66 | 3.08E-03 | 0.465 | 0.05 | 6.40E-03 | 3.63E-03 |
| rs34273148 | 8 | 104266195 | C | T | 0.89 | 1.47 (1.21-1.78) | 5.79E-05 | 0.549 | 0.549 | 0.63 | 3.77E-03 | 0.532 | 0.04 | 5.25E-03 | 4.07E-03 |
| rs13278540 | 8 | 104269544 | G | A | 0.86 | 1.41 (1.19-1.67) | 5.76E-05 | 0.901 | 0.901 | 0.62 | 1.34E-03 | 0.316 | 0.1 | 5.74E-02 | 1.37E-04 |
| rs13278361 | 8 | 104269649 | C | T | 0.89 | 1.47 (1.21-1.78) | 5.86E-05 | 0.552 | 0.552 | 0.63 | 3.76E-03 | 0.528 | 0.04 | 5.25E-03 | 4.08E-03 |
| rs3133824 | 8 | 104276312 | T | C | 0.89 | 1.47 (1.21-1.78) | 6.05E-05 | 0.561 | 0.561 | 0.64 | 3.74E-03 | 0.519 | 0.04 | 5.26E-03 | 4.09E-03 |
| rs1138873 | 8 | 104279038 | G | A | 0.86 | 1.41 (1.19-1.67) | 6.71E-05 | 0.914 | 0.914 | 0.63 | 1.44E-03 | 0.299 | 0.11 | 5.88E-02 | 1.53E-04 |
| rs2220826 | 8 | 104281357 | C | A | 0.65 | 1.32 (1.17-1.49) | 5.58E-06 | 0.432 | 0.581 | 0.21 | 7.19E-04 | 0.783 | 0.01 | 5.42E-04 | 1.90E-03 |
| rs4115670 | 8 | 104312715 | G | A | 0.49 | 0.8 (0.71-0.9) | 1.03E-04 | 0.17 | 0.17 | 0.67 | 1.11E-02 | 0.89 | 0.01 | 4.07E-02 | 2.03E-04 |
| rs3133832 | 8 | 104357600 | C | T | 0.92 | 1.58 (1.26-1.99) | 5.55E-05 | 0.56 | 0.56 | 0.69 | 3.57E-03 | 0.471 | 0.05 | 1.16E-02 | 2.84E-03 |
| rs1965482 | 8 | 104359449 | C | G | 0.92 | 1.6 (1.27-2.01) | 3.37E-05 | 0.489 | 0.489 | 0.55 | 3.15E-03 | 0.513 | 0.03 | 8.91E-03 | 2.11E-03 |
| rs3133834 | 8 | 104362174 | C | G | 0.92 | 1.57 (1.25-1.97) | 6.99E-05 | 0.559 | 0.559 | 0.66 | 4.06E-03 | 0.471 | 0.05 | 1.43E-02 | 2.52E-03 |
| rs3133835 | 8 | 104363126 | G | A | 0.92 | 1.53 (1.23-1.92) | 1.26E-04 | 0.512 | 0.512 | 0.58 | 6.13E-03 | 0.504 | 0.05 | 1.66E-02 | 3.60E-03 |
| rs3133836 | 8 | 104363338 | T | C | 0.92 | 1.53 (1.22-1.92) | 1.27E-04 | 0.524 | 0.524 | 0.57 | 5.98E-03 | 0.494 | 0.05 | 1.67E-02 | 3.59E-03 |
| rs1755276 | 9 | 17942032 | A | G | 0.99 | 0.38 (0.23-0.62) | 7.99E-05 | 0.026 | 0.026 | 0.63 | 8.63E-06 | 8.63E-06 | 0.8 | 3.63E-02 | 1.13E-02 |
| rs2383057 | 9 | 17946622 | T | A | 0.99 | 0.38 (0.23-0.62) | 8.62E-05 | 0.028 | 0.028 | 0.58 | 9.82E-06 | 0 | 0.74 | 3.49E-02 | 1.23E-02 |
| rs2840782 | 9 | 17947425 | A | G | 0.99 | 0.38 (0.23-0.62) | 8.63E-05 | 0.03 | 0.03 | 0.58 | 1.03E-05 | 0 | 0.73 | 3.49E-02 | 1.23E-02 |
| rs2772690 | 9 | 17947742 | G | A | 0.99 | 0.38 (0.23-0.62) | 8.62E-05 | 0.024 | 0.024 | 0.63 | 8.29E-06 | 0 | 0.79 | 3.49E-02 | 1.22E-02 |
| rs2811824 | 9 | 17948254 | C | A | 0.99 | 0.38 (0.23-0.62) | 8.63E-05 | 0.03 | 0.03 | 0.57 | 1.05E-05 | 0 | 0.73 | 3.49E-02 | 1.22E-02 |
| rs2772692 | 9 | 17949040 | G | A | 0.99 | 0.38 (0.23-0.62) | 8.67E-05 | 0.03 | 0.03 | 0.58 | 1.04E-05 | 0 | 0.73 | 3.49E-02 | 1.22E-02 |
| rs2840779 | 9 | 17950343 | G | T | 0.99 | 0.38 (0.23-0.62) | 8.69E-05 | 0.029 | 0.029 | 0.58 | 1.02E-05 | 0 | 0.73 | 3.49E-02 | 1.21E-02 |
| rs145162794 | 9 | 17959449 | A | G | 0.99 | 0.38 (0.23-0.61) | 4.90E-05 | 0.027 | 0.027 | 0.72 | 5.45E-06 | 0 | 0.86 | 1.00E-02 | 2.37E-02 |
| rs113609979 | 9 | 17961146 | T | G | 0.99 | 0.37 (0.23-0.6) | 3.55E-05 | 0.026 | 0.026 | 0.72 | 3.94E-06 | 0 | 0.86 | 9.70E-03 | 1.80E-02 |
| rs1618634 | 9 | 17962581 | G | T | 0.99 | 0.37 (0.23-0.6) | 2.84E-05 | 0.072 | 0.097 | 0.31 | 1.02E-05 | 0 | 0.41 | 1.89E-02 | 7.30E-03 |
| rs2811805 | 9 | 17962856 | T | C | 0.99 | 0.37 (0.23-0.6) | 3.06E-05 | 0.071 | 0.096 | 0.31 | 1.08E-05 | 0 | 0.41 | 1.93E-02 | 7.38E-03 |
| rs1778181 | 9 | 17964230 | T | A | 0.99 | 0.37 (0.23-0.6) | 3.47E-05 | 0.024 | 0.024 | 0.69 | 3.72E-06 | 3.72E-06 | 0.84 | 2.01E-02 | 7.47E-03 |
| 9:35969579:G_GT | 9 | 35969579 | R | I | 0.77 | 0.74 (0.64-0.85) | 3.49E-05 | 0.113 | 0.113 | 0.67 | 6.13E-03 | 0.925 | 0 | 6.52E-03 | 1.03E-02 |
| rs10217546 | 9 | 35986887 | C | A | 0.54 | 0.82 (0.73-0.92) | 8.89E-04 | 0.04 | 0.04 | 0.83 | 8.24E-02 | 0.634 | 0.01 | 2.59E-02 | 3.21E-02 |
| rs1359164 | 9 | 85811127 | G | C | 0.64 | 1.3 (1.15-1.47) | 2.46E-05 | 0.235 | 0.235 | 0.67 | 3.73E-03 | 0.758 | 0.01 | 2.33E-03 | 3.46E-03 |
| rs1359165 | 9 | 85811229 | C | G | 0.64 | 1.29 (1.14-1.46) | 4.34E-05 | 0.329 | 0.329 | 0.67 | 3.65E-03 | 0.671 | 0.02 | 3.48E-03 | 4.51E-03 |
| rs11795061 | 9 | 85811478 | A | T | 0.64 | 1.29 (1.14-1.46) | 4.55E-05 | 0.309 | 0.309 | 0.65 | 4.25E-03 | 0.688 | 0.02 | 4.41E-03 | 3.43E-03 |
| rs11139953 | 9 | 85811540 | C | T | 0.64 | 1.29 (1.14-1.46) | 3.80E-05 | 0.296 | 0.296 | 0.66 | 3.98E-03 | 0.697 | 0.02 | 3.57E-03 | 3.57E-03 |
| rs1323765 | 9 | 85811734 | G | C | 0.64 | 1.29 (1.14-1.46) | 3.81E-05 | 0.297 | 0.297 | 0.65 | 3.93E-03 | 0.696 | 0.02 | 3.58E-03 | 3.55E-03 |
| rs1323766 | 9 | 85811825 | G | A | 0.64 | 1.28 (1.14-1.44) | 4.59E-05 | 0.352 | 0.352 | 0.65 | 3.64E-03 | 0.648 | 0.02 | 5.32E-03 | 2.92E-03 |
| rs7864580 | 9 | 85812142 | C | A | 0.64 | 1.27 (1.13-1.44) | 5.81E-05 | 0.288 | 0.288 | 0.63 | 5.28E-03 | 0.709 | 0.02 | 6.12E-03 | 3.32E-03 |
| rs1830772 | 9 | 85812407 | G | T | 0.64 | 1.28 (1.14-1.44) | 4.29E-05 | 0.3 | 0.3 | 0.62 | 4.18E-03 | 0.691 | 0.02 | 5.09E-03 | 2.80E-03 |
| rs7041473 | 9 | 85812980 | C | A | 0.64 | 1.28 (1.14-1.44) | 4.26E-05 | 0.291 | 0.291 | 0.63 | 4.34E-03 | 0.701 | 0.02 | 5.10E-03 | 2.77E-03 |
| rs7041613 | 9 | 85813004 | T | C | 0.64 | 1.28 (1.14-1.44) | 4.26E-05 | 0.291 | 0.291 | 0.63 | 4.34E-03 | 0.701 | 0.02 | 5.10E-03 | 2.76E-03 |
| rs7024372 | 9 | 85816527 | A | G | 0.51 | 0.8 (0.71-0.9) | 1.19E-04 | 0.84 | 0.84 | 0.76 | 1.60E-03 | 0.247 | 0.15 | 1.46E-02 | 4.15E-03 |
| rs34600500 | 9 | 85822179 | G | A | 0.51 | 0.8 (0.72-0.9) | 1.38E-04 | 0.889 | 0.889 | 0.78 | 1.58E-03 | 0.204 | 0.17 | 1.41E-02 | 5.86E-03 |
| rs12335503 | 9 | 85826840 | C | T | 0.51 | 0.81 (0.72-0.91) | 2.54E-04 | 0.989 | 0.989 | 0.82 | 2.05E-03 | 0.111 | 0.24 | 1.61E-02 | 1.16E-02 |
| rs2181711 | 9 | 85830083 | C | T | 0.65 | 1.3 (1.16-1.47) | 1.36E-05 | 0.532 | 0.532 | 0.78 | 9.13E-04 | 0.506 | 0.04 | 3.37E-03 | 1.99E-03 |
| rs13301143 | 9 | 85830147 | C | T | 0.52 | 0.8 (0.72-0.9) | 1.64E-04 | 0.628 | 0.628 | 0.93 | 3.49E-03 | 0.409 | 0.11 | 1.03E-02 | 8.55E-03 |
| rs12340847 | 9 | 85831163 | G | T | 0.52 | 0.8 (0.71-0.9) | 1.48E-04 | 0.649 | 0.649 | 0.93 | 3.09E-03 | 0.392 | 0.12 | 9.55E-03 | 8.38E-03 |
| rs11139967 | 9 | 85831498 | G | A | 0.52 | 0.8 (0.71-0.9) | 1.55E-04 | 0.673 | 0.673 | 0.91 | 3.03E-03 | 0.37 | 0.12 | 9.92E-03 | 8.45E-03 |
| rs13292972 | 9 | 85832367 | C | G | 0.64 | 1.32 (1.17-1.49) | 4.37E-06 | 0.588 | 0.588 | 0.69 | 3.56E-04 | 0.462 | 0.03 | 1.16E-03 | 1.75E-03 |
| rs13293114 | 9 | 85832384 | A | G | 0.64 | 1.32 (1.17-1.49) | 4.35E-06 | 0.588 | 0.588 | 0.69 | 3.55E-04 | 0.462 | 0.03 | 1.15E-03 | 1.76E-03 |
| rs7848774 | 9 | 85832774 | A | T | 0.8 | 1.34 (1.16-1.55) | 6.10E-05 | 0.302 | 0.302 | 0.52 | 5.69E-03 | 0.701 | 0.02 | 1.31E-03 | 5.60E-02 |
| rs10125314 | 9 | 85833248 | G | A | 0.57 | 1.28 (1.14-1.43) | 2.62E-05 | 0.81 | 0.81 | 0.39 | 6.35E-04 | 0.333 | 0.05 | 1.75E-03 | 1.31E-02 |
| rs1323772 | 9 | 85833752 | G | A | 0.8 | 1.36 (1.18-1.58) | 3.21E-05 | 0.512 | 0.512 | 0.54 | 1.87E-03 | 0.528 | 0.03 | 6.13E-04 | 7.62E-02 |
| rs11139975 | 9 | 85834312 | G | A | 0.8 | 1.36 (1.18-1.57) | 2.76E-05 | 0.467 | 0.467 | 0.47 | 1.97E-03 | 0.55 | 0.02 | 1.68E-03 | 2.96E-02 |
| rs1408101 | 9 | 85834349 | G | T | 0.58 | 1.25 (1.12-1.4) | 1.14E-04 | 0.991 | 0.991 | 0.4 | 1.17E-03 | 0.233 | 0.11 | 5.48E-03 | 2.55E-02 |
| rs1408102 | 9 | 85834374 | A | G | 0.58 | 1.25 (1.12-1.4) | 1.16E-04 | 0.991 | 0.991 | 0.4 | 1.19E-03 | 0.233 | 0.11 | 5.50E-03 | 2.58E-02 |
| rs1408103 | 9 | 85834617 | T | A | 0.58 | 1.25 (1.12-1.41) | 1.11E-04 | 0.986 | 0.986 | 0.4 | 1.16E-03 | 0.236 | 0.11 | 5.58E-03 | 2.41E-02 |
| rs12237222 | 9 | 85834743 | T | G | 0.58 | 1.25 (1.12-1.4) | 1.16E-04 | 0.978 | 0.978 | 0.39 | 1.22E-03 | 0.242 | 0.11 | 5.65E-03 | 2.47E-02 |
| rs10512404 | 9 | 113035784 | A | G | 0.84 | 1.4 (1.19-1.65) | 3.86E-05 | 0.129 | 0.129 | 0.56 | 1.24E-02 | 0.842 | 0 | 3.68E-02 | 1.32E-04 |
| rs59394137 | 9 | 113036125 | A | G | 0.83 | 1.41 (1.2-1.66) | 2.23E-05 | 0.127 | 0.127 | 0.56 | 9.11E-03 | 0.853 | 0 | 3.11E-02 | 9.66E-05 |
| rs59345897 | 9 | 113037297 | T | C | 0.84 | 1.41 (1.2-1.65) | 2.44E-05 | 0.117 | 0.117 | 0.56 | 1.03E-02 | 0.838 | 0 | 3.43E-02 | 8.69E-05 |
| rs61002198 | 9 | 113039645 | A | C | 0.84 | 1.41 (1.2-1.66) | 2.13E-05 | 0.102 | 0.102 | 0.55 | 1.11E-02 | 0.808 | 0 | 4.22E-02 | 5.86E-05 |
| 9:113040338:C_CT | 9 | 113040338 | R | I | 0.88 | 1.49 (1.23-1.81) | 2.55E-05 | 0.199 | 0.199 | 0.71 | 4.94E-03 | 0.994 | 0.01 | 2.22E-04 | 2.60E-02 |
| rs62580774 | 9 | 113042337 | T | C | 0.84 | 1.41 (1.2-1.65) | 1.97E-05 | 0.097 | 0.097 | 0.56 | 1.10E-02 | 0.802 | 0 | 4.39E-02 | 4.92E-05 |
| rs62580776 | 9 | 113042514 | G | C | 0.84 | 1.41 (1.2-1.65) | 1.95E-05 | 0.097 | 0.097 | 0.56 | 1.10E-02 | 0.801 | 0 | 4.41E-02 | 4.84E-05 |
| rs55939008 | 9 | 113043945 | T | C | 0.84 | 1.41 (1.2-1.65) | 1.90E-05 | 0.095 | 0.095 | 0.56 | 1.11E-02 | 0.799 | 0 | 4.46E-02 | 4.62E-05 |
| rs10980300 | 9 | 113045164 | G | A | 0.84 | 1.41 (1.2-1.66) | 1.80E-05 | 0.091 | 0.091 | 0.56 | 1.13E-02 | 0.792 | 0 | 4.34E-02 | 4.57E-05 |
| rs10816959 | 9 | 113046742 | A | G | 0.66 | 1.27 (1.13-1.43) | 8.55E-05 | 0.444 | 0.444 | 0.82 | 3.91E-03 | 0.717 | 0.05 | 2.51E-02 | 1.37E-03 |
| rs60702108 | 9 | 113047837 | T | C | 0.84 | 1.42 (1.21-1.67) | 1.75E-05 | 0.08 | 0.08 | 0.53 | 1.22E-02 | 0.776 | 0 | 4.41E-02 | 4.35E-05 |
| rs62580781 | 9 | 113049069 | G | A | 0.84 | 1.4 (1.19-1.64) | 2.52E-05 | 0.092 | 0.092 | 0.55 | 1.31E-02 | 0.79 | 0 | 5.88E-02 | 4.38E-05 |
| rs62580784 | 9 | 113050126 | C | G | 0.84 | 1.4 (1.19-1.64) | 2.77E-05 | 0.092 | 0.092 | 0.54 | 1.35E-02 | 0.792 | 0 | 6.28E-02 | 4.50E-05 |
| rs10980301 | 9 | 113050873 | C | A | 0.84 | 1.4 (1.19-1.64) | 2.88E-05 | 0.095 | 0.095 | 0.54 | 1.35E-02 | 0.795 | 0 | 6.45E-02 | 4.55E-05 |
| 9:113052534:T_TC | 9 | 113052534 | R | I | 0.85 | 1.41 (1.2-1.67) | 3.27E-05 | 0.174 | 0.174 | 0.46 | 8.62E-03 | 0.893 | 0 | 1.10E-04 | 4.54E-02 |
| rs59210554 | 9 | 135012819 | A | G | 0.77 | 0.79 (0.69-0.9) | 5.72E-04 | 0.22 | 0.254 | 0.35 | 3.62E-04 | 3.62E-04 | 0.43 | 1.11E-02 | 3.11E-02 |
| rs72763658 | 9 | 135014038 | G | T | 0.77 | 0.79 (0.69-0.9) | 5.74E-04 | 0.227 | 0.289 | 0.34 | 3.77E-04 | 3.77E-04 | 0.41 | 1.13E-02 | 3.12E-02 |
| rs192386529 | 10 | 13664943 | A | G | 0.97 | 0.45 (0.31-0.66) | 2.80E-05 | 0.962 | 0.962 | 0.47 | 3.66E-04 | 0.241 | 0.09 | 1.81E-01 | 2.20E-05 |
| rs11013837 | 10 | 24284651 | T | A | 0.77 | 1.37 (1.19-1.58) | 6.40E-06 | 0.37 | 0.37 | 0.61 | 2.44E-05 | 0.007 | 0.28 | 1.94E-04 | 1.96E-02 |
| rs12358374 | 10 | 24287188 | C | A | 0.8 | 1.36 (1.18-1.58) | 2.89E-05 | 0.225 | 0.494 | 0.2 | 3.27E-05 | 0.024 | 0.22 | 4.42E-04 | 1.94E-02 |
| rs4750773 | 10 | 131583388 | G | A | 0.6 | 0.79 (0.7-0.88) | 4.88E-05 | 0.754 | 0.516 | 0.06 | 1.18E-03 | 0.863 | 0.01 | 1.74E-02 | 1.35E-03 |
| rs4751122 | 10 | 131583538 | C | G | 0.59 | 0.78 (0.7-0.88) | 2.88E-05 | 0.822 | 0.531 | 0.06 | 6.74E-04 | 0.832 | 0.01 | 1.05E-02 | 1.24E-03 |
| rs7908271 | 10 | 131584161 | G | A | 0.61 | 0.77 (0.69-0.87) | 3.66E-05 | 0.826 | 0.541 | 0.08 | 8.14E-04 | 0.814 | 0.01 | 6.92E-03 | 2.11E-03 |
| rs913114 | 10 | 131584627 | C | T | 0.54 | 0.78 (0.7-0.88) | 3.05E-05 | 0.652 | 0.437 | 0.05 | 1.07E-03 | 0.986 | 0 | 1.11E-03 | 5.47E-03 |
| rs11816476 | 10 | 131585846 | C | T | 0.54 | 0.78 (0.7-0.88) | 3.33E-05 | 0.64 | 0.433 | 0.05 | 1.17E-03 | 0.995 | 0 | 1.15E-03 | 5.68E-03 |
| rs3750832 | 10 | 131587140 | G | A | 0.54 | 0.79 (0.7-0.88) | 4.62E-05 | 0.633 | 0.432 | 0.05 | 1.48E-03 | 0.998 | 0.01 | 1.59E-03 | 5.82E-03 |
| rs35028872 | 10 | 131588705 | A | G | 0.59 | 0.79 (0.7-0.88) | 4.66E-05 | 0.776 | 0.518 | 0.06 | 1.04E-03 | 0.855 | 0.01 | 1.07E-02 | 1.82E-03 |
| rs7933282 | 11 | 7400623 | A | G | 0.58 | 1.32 (1.18-1.49) | 2.27E-06 | 0.793 | 0.976 | 0.25 | 1.18E-04 | 0.329 | 0.02 | 1.65E-03 | 7.78E-04 |
| rs7947307 | 11 | 7400755 | C | T | 0.58 | 1.32 (1.18-1.49) | 2.37E-06 | 0.802 | 0.965 | 0.25 | 1.20E-04 | 0.326 | 0.02 | 1.78E-03 | 7.51E-04 |
| rs1023996 | 11 | 7400790 | G | A | 0.58 | 1.32 (1.18-1.48) | 2.27E-06 | 0.797 | 0.959 | 0.24 | 1.17E-04 | 0.329 | 0.02 | 1.65E-03 | 7.77E-04 |
| rs7108135 | 11 | 8402917 | A | G | 0.93 | 1.66 (1.3-2.13) | 3.59E-05 | 0.445 | 0.445 | 0.32 | 2.35E-03 | 0.47 | 0.01 | 1.55E-02 | 3.78E-03 |
| rs7122354 | 11 | 8403190 | G | A | 0.93 | 1.66 (1.29-2.14) | 4.23E-05 | 0.444 | 0.444 | 0.33 | 2.74E-03 | 0.471 | 0.01 | 1.65E-02 | 3.90E-03 |
| rs7123170 | 11 | 8403803 | G | A | 0.93 | 1.66 (1.29-2.12) | 3.78E-05 | 0.447 | 0.447 | 0.33 | 2.43E-03 | 0.47 | 0.02 | 1.56E-02 | 3.79E-03 |
| rs10840044 | 11 | 8446940 | G | C | 0.94 | 1.73 (1.32-2.26) | 3.45E-05 | 0.297 | 0.297 | 0.36 | 4.48E-03 | 0.552 | 0.01 | 2.03E-02 | 3.65E-03 |
| rs10840045 | 11 | 8452471 | T | C | 0.94 | 1.79 (1.36-2.35) | 1.73E-05 | 0.284 | 0.284 | 0.4 | 3.35E-03 | 0.557 | 0.01 | 1.65E-02 | 2.56E-03 |
| rs55740932 | 11 | 8453025 | C | A | 0.94 | 1.79 (1.36-2.35) | 1.72E-05 | 0.302 | 0.302 | 0.4 | 3.12E-03 | 0.546 | 0.01 | 1.65E-02 | 2.56E-03 |
| rs10459005 | 11 | 103416881 | T | C | 0.77 | 1.34 (1.16-1.54) | 4.12E-05 | 0.861 | 0.861 | 0.5 | 9.70E-04 | 0.298 | 0.07 | 6.11E-03 | 5.69E-03 |
| rs4754082 | 11 | 103417927 | G | A | 0.78 | 1.33 (1.16-1.53) | 4.89E-05 | 0.894 | 0.895 | 0.37 | 1.05E-03 | 0.295 | 0.06 | 2.49E-03 | 1.51E-02 |
| rs10895460 | 11 | 103420519 | C | G | 0.78 | 1.33 (1.16-1.53) | 4.32E-05 | 0.717 | 0.717 | 0.38 | 1.54E-03 | 0.386 | 0.04 | 2.08E-03 | 1.56E-02 |
| 11:103738106:TTTTG | 11 | 103738106 | D | R | 0.8 | 1.37 (1.17-1.6) | 4.98E-05 | 0.252 | 0.252 | 0.52 | 8.49E-03 | 0.693 | 0.01 | 7.68E-04 | 1.33E-02 |
| rs965505 | 11 | 103739232 | C | T | 0.81 | 1.37 (1.18-1.6) | 3.55E-05 | 0.205 | 0.205 | 0.52 | 8.66E-03 | 0.749 | 0.01 | 1.08E-03 | 9.91E-03 |
| rs471165 | 11 | 103740462 | G | A | 0.81 | 1.37 (1.18-1.59) | 4.29E-05 | 0.212 | 0.212 | 0.52 | 9.36E-03 | 0.742 | 0.01 | 1.34E-03 | 9.81E-03 |
| rs61917308 | 12 | 68029527 | T | C | 0.86 | 0.7 (0.59-0.83) | 3.71E-05 | 0.698 | 0.698 | 0.85 | 1.45E-04 | 0.001 | 0.37 | 2.20E-02 | 3.11E-03 |
| rs7302318 | 12 | 68094969 | R | I | 0.85 | 0.7 (0.59-0.83) | 3.17E-05 | 0.241 | 0.241 | 0.63 | 3.30E-05 | 0 | 0.48 | 4.10E-02 | 1.21E-03 |
| rs10848145 | 12 | 94245578 | G | A | 0.86 | 0.69 (0.59-0.82) | 1.05E-05 | 0.549 | 0.44 | 0.02 | 5.30E-05 | 0.185 | 0.01 | 1.08E-01 | 1.32E-05 |
| rs71451375 | 12 | 131039814 | C | T | 0.53 | 0.78 (0.7-0.88) | 2.88E-05 | 0.254 | 0.27 | 0.01 | 3.57E-05 | 0.123 | 0.01 | 3.03E-03 | 4.20E-02 |
| rs4759493 | 12 | 131042460 | G | T | 0.53 | 0.79 (0.7-0.88) | 3.77E-05 | 0.294 | 0.295 | 0.01 | 5.51E-05 | 0.135 | 0.02 | 2.48E-03 | 5.61E-02 |
| rs7311454 | 12 | 131044768 | C | T | 0.57 | 0.77 (0.68-0.86) | 6.22E-06 | 0.217 | 0.262 | 0.03 | 7.79E-06 | 0.082 | 0.03 | 1.66E-03 | 2.94E-02 |
| rs7975553 | 12 | 131045130 | G | A | 0.57 | 0.77 (0.68-0.86) | 5.95E-06 | 0.218 | 0.262 | 0.03 | 7.53E-06 | 0.083 | 0.03 | 1.58E-03 | 2.94E-02 |
| rs9931034 | 16 | 9506148 | G | A | 0.87 | 1.46 (1.22-1.74) | 1.86E-05 | 0.481 | 0.481 | 0.92 | 5.90E-05 | 0 | 0.36 | 2.98E-03 | 5.76E-03 |
| rs73511306 | 16 | 9507575 | T | C | 0.86 | 1.46 (1.22-1.73) | 1.83E-05 | 0.547 | 0.547 | 0.89 | 7.16E-05 | 0.002 | 0.32 | 2.89E-03 | 5.87E-03 |
| rs12325488 | 16 | 9509850 | A | G | 0.86 | 1.46 (1.24-1.73) | 6.48E-06 | 0.511 | 0.511 | 0.78 | 2.91E-05 | 0.006 | 0.27 | 3.60E-03 | 1.67E-03 |
| rs148851398 | 16 | 9510488 | G | T | 0.98 | 2.89 (1.74-4.8) | 6.77E-06 | 0.636 | 0.636 | 0.61 | 1.17E-03 | 0.519 | 0.03 | 1.92E-02 | 9.90E-04 |
| rs727053 | 16 | 9512218 | G | A | 0.87 | 1.44 (1.21-1.72) | 2.68E-05 | 0.589 | 0.589 | 0.81 | 1.13E-04 | 0.006 | 0.3 | 2.84E-03 | 8.63E-03 |
| rs112156521 | 16 | 9514318 | C | G | 0.86 | 1.44 (1.21-1.72) | 2.65E-05 | 0.554 | 0.554 | 0.83 | 9.96E-05 | 0.002 | 0.32 | 3.19E-03 | 8.32E-03 |
| rs73494611 | 16 | 9514370 | T | G | 0.87 | 1.43 (1.21-1.71) | 3.36E-05 | 0.62 | 0.62 | 0.81 | 1.47E-04 | 0.009 | 0.29 | 3.13E-03 | 9.96E-03 |
| rs58317623 | 16 | 9514658 | G | A | 0.87 | 1.43 (1.21-1.71) | 3.40E-05 | 0.537 | 0.537 | 0.84 | 1.15E-04 | 0.001 | 0.35 | 3.13E-03 | 1.01E-02 |
| rs60782078 | 16 | 9514707 | C | T | 0.87 | 1.43 (1.21-1.7) | 3.43E-05 | 0.537 | 0.537 | 0.84 | 1.15E-04 | 0.001 | 0.35 | 3.13E-03 | 1.01E-02 |
| rs9302525 | 16 | 9515402 | C | A | 0.86 | 1.45 (1.22-1.71) | 1.06E-05 | 0.442 | 0.442 | 0.74 | 3.38E-05 | 0 | 0.33 | 3.70E-03 | 2.69E-03 |
| rs73494614 | 16 | 9515438 | A | G | 0.85 | 1.44 (1.23-1.7) | 6.55E-06 | 0.423 | 0.423 | 0.77 | 1.95E-05 | 0 | 0.35 | 2.99E-04 | 1.39E-02 |
| rs73494616 | 16 | 9515766 | C | T | 0.87 | 1.43 (1.2-1.7) | 3.50E-05 | 0.551 | 0.551 | 0.83 | 1.22E-04 | 0.001 | 0.34 | 3.10E-03 | 1.04E-02 |
| rs12449072 | 16 | 9517454 | C | T | 0.87 | 1.44 (1.21-1.71) | 3.03E-05 | 0.557 | 0.557 | 0.83 | 1.11E-04 | 0.002 | 0.33 | 3.09E-03 | 9.04E-03 |
| rs12102909 | 16 | 9517561 | G | A | 0.86 | 1.45 (1.23-1.72) | 8.49E-06 | 0.443 | 0.443 | 0.75 | 2.87E-05 | 0.001 | 0.32 | 3.32E-03 | 2.28E-03 |
| rs9937969 | 16 | 9519053 | G | C | 0.87 | 1.44 (1.21-1.71) | 3.13E-05 | 0.543 | 0.543 | 0.85 | 1.09E-04 | 0.001 | 0.34 | 2.94E-03 | 9.33E-03 |
| 16:9519073:GAGAA | 16 | 9519073 | R | D | 0.87 | 1.44 (1.21-1.71) | 3.08E-05 | 0.555 | 0.555 | 0.84 | 1.12E-04 | 0.001 | 0.33 | 2.94E-03 | 9.38E-03 |
| rs16965238 | 16 | 9522660 | A | G | 0.86 | 1.46 (1.23-1.74) | 9.21E-06 | 0.493 | 0.493 | 0.51 | 3.11E-05 | 0.005 | 0.29 | 2.96E-04 | 1.57E-02 |
| rs9931860 | 16 | 9523949 | G | C | 0.86 | 1.45 (1.22-1.73) | 1.57E-05 | 0.39 | 0.39 | 0.47 | 3.34E-05 | 0 | 0.36 | 3.53E-04 | 1.84E-02 |
| rs16965242 | 16 | 9524459 | C | T | 0.86 | 1.45 (1.23-1.72) | 1.21E-05 | 0.468 | 0.468 | 0.53 | 3.53E-05 | 0.001 | 0.32 | 3.32E-04 | 1.80E-02 |
| rs74010816 | 16 | 9524826 | A | T | 0.86 | 1.45 (1.23-1.72) | 1.23E-05 | 0.471 | 0.471 | 0.51 | 3.60E-05 | 0.002 | 0.32 | 3.31E-04 | 1.81E-02 |
| rs1964340 | 16 | 9534486 | G | A | 0.85 | 1.41 (1.2-1.65) | 2.76E-05 | 0.453 | 0.453 | 0.71 | 6.74E-05 | 0 | 0.4 | 4.70E-04 | 2.86E-02 |
| rs7225656 | 17 | 2913063 | G | A | 0.55 | 1.27 (1.14-1.43) | 3.63E-05 | 0.27 | 0.27 | 0.87 | 4.91E-05 | 0 | 0.59 | 3.18E-03 | 1.03E-02 |
| rs8069242 | 17 | 2914224 | T | G | 0.55 | 1.28 (1.14-1.43) | 3.70E-05 | 0.288 | 0.288 | 0.9 | 5.58E-05 | 0 | 0.58 | 2.78E-03 | 1.18E-02 |
| rs4359482 | 17 | 2915699 | A | G | 0.55 | 1.29 (1.15-1.45) | 1.44E-05 | 0.264 | 0.264 | 0.93 | 2.30E-05 | 0 | 0.55 | 3.81E-03 | 3.92E-03 |
| rs9902489 | 17 | 2915772 | T | C | 0.56 | 1.28 (1.14-1.44) | 2.84E-05 | 0.255 | 0.255 | 0.9 | 3.75E-05 | 0 | 0.6 | 4.47E-03 | 5.64E-03 |
| rs9908715 | 17 | 2916117 | A | C | 0.56 | 1.28 (1.14-1.44) | 2.87E-05 | 0.262 | 0.262 | 0.9 | 3.92E-05 | 0 | 0.59 | 4.66E-03 | 5.46E-03 |
| rs72904570 | 18 | 36585235 | G | C | 0.72 | 0.75 (0.65-0.85) | 1.62E-05 | 0.028 | 0.131 | 0.16 | 1.37E-06 | 0 | 0.29 | 1.28E-05 | 3.73E-02 |
| rs17659787 | 18 | 36600043 | T | A | 0.71 | 0.75 (0.65-0.85) | 1.31E-05 | 0.028 | 0.129 | 0.17 | 1.14E-06 | 0 | 0.3 | 8.28E-06 | 4.29E-02 |
| rs72886417 | 18 | 36602286 | G | A | 0.71 | 0.75 (0.66-0.85) | 1.31E-05 | 0.029 | 0.129 | 0.17 | 1.13E-06 | 0 | 0.3 | 8.17E-06 | 4.31E-02 |
| rs72886457 | 18 | 36643966 | C | T | 0.7 | 0.76 (0.67-0.86) | 1.99E-05 | 0.036 | 0.093 | 0.25 | 2.09E-06 | 0 | 0.41 | 2.86E-05 | 3.69E-02 |
| rs6507874 | 18 | 46448805 | T | C | 0.55 | 1.28 (1.14-1.44) | 4.09E-05 | 0.2 | 0.2 | 0.81 | 3.49E-05 | 0 | 0.66 | 1.61E-01 | 2.16E-05 |
| rs8085824 | 18 | 46449111 | T | C | 0.54 | 0.77 (0.69-0.87) | 1.20E-05 | 0.306 | 0.306 | 0.84 | 2.30E-05 | 0 | 0.47 | 2.00E-02 | 1.28E-04 |
| rs58920878 | 18 | 46449565 | C | G | 0.54 | 0.78 (0.69-0.87) | 2.28E-05 | 0.202 | 0.202 | 0.65 | 2.11E-05 | 0 | 0.52 | 1.70E-02 | 3.77E-04 |
| rs34007497 | 18 | 46451073 | C | G | 0.54 | 0.78 (0.69-0.87) | 1.19E-05 | 0.292 | 0.292 | 0.81 | 2.04E-05 | 0 | 0.48 | 1.72E-02 | 1.62E-04 |
| rs12953717 | 18 | 46453929 | C | T | 0.54 | 0.78 (0.69-0.87) | 1.25E-05 | 0.288 | 0.288 | 0.84 | 2.09E-05 | 0 | 0.5 | 1.64E-02 | 1.71E-04 |
| rs73927509 | 19 | 14793072 | I | R | 0.56 | 1.28 (1.13-1.44) | 4.86E-05 | 0.821 | 0.555 | 0.06 | 4.81E-04 | 0.304 | 0.02 | 1.29E-02 | 5.74E-03 |
| rs12982955 | 19 | 14811834 | A | G | 0.52 | 1.28 (1.14-1.44) | 1.84E-05 | 0.423 | 0.659 | 0.05 | 5.94E-04 | 0.419 | 0 | 5.81E-03 | 1.17E-02 |
| rs3850143 | 19 | 14812356 | G | A | 0.52 | 1.28 (1.14-1.44) | 1.85E-05 | 0.441 | 0.647 | 0.05 | 5.61E-04 | 0.411 | 0 | 5.84E-03 | 1.19E-02 |
| rs4808319 | 19 | 14817062 | A | G | 0.52 | 1.28 (1.14-1.44) | 2.05E-05 | 0.415 | 0.666 | 0.05 | 6.63E-04 | 0.426 | 0 | 6.25E-03 | 1.19E-02 |
| rs11670629 | 19 | 14822027 | A | G | 0.5 | 1.3 (1.15-1.46) | 1.42E-05 | 0.552 | 0.619 | 0.05 | 3.30E-04 | 0.371 | 0.01 | 5.74E-03 | 9.08E-03 |
| rs11085908 | 19 | 14822559 | G | T | 0.52 | 1.29 (1.15-1.44) | 1.57E-05 | 0.436 | 0.646 | 0.04 | 4.93E-04 | 0.41 | 0 | 4.52E-03 | 1.28E-02 |
| rs73014710 | 19 | 14825265 | A | G | 0.52 | 1.29 (1.15-1.44) | 1.46E-05 | 0.516 | 0.619 | 0.04 | 3.59E-04 | 0.382 | 0.01 | 4.52E-03 | 1.25E-02 |
| rs3752185 | 19 | 14828737 | A | G | 0.52 | 1.29 (1.15-1.44) | 1.41E-05 | 0.546 | 0.604 | 0.04 | 3.11E-04 | 0.371 | 0.01 | 4.52E-03 | 1.25E-02 |
| rs11666622 | 19 | 14830568 | T | G | 0.52 | 1.28 (1.15-1.44) | 1.65E-05 | 0.539 | 0.602 | 0.04 | 3.52E-04 | 0.375 | 0.01 | 4.52E-03 | 1.37E-02 |
| rs11085909 | 19 | 14830749 | A | G | 0.52 | 1.28 (1.14-1.44) | 2.21E-05 | 0.407 | 0.661 | 0.05 | 6.19E-04 | 0.435 | 0 | 5.20E-03 | 1.62E-02 |
| rs11670658 | 19 | 14836559 | C | T | 0.52 | 1.29 (1.15-1.44) | 1.68E-05 | 0.564 | 0.593 | 0.04 | 3.37E-04 | 0.365 | 0.01 | 4.67E-03 | 1.39E-02 |
| rs344801 | 19 | 45805147 | A | G | 0.66 | 1.23 (1.09-1.4) | 1.04E-03 | 0.834 | 0.773 | 0.22 | 4.86E-03 | 0.315 | 0.12 | 1.56E-01 | 3.92E-03 |
| rs326458 | 19 | 53027520 | C | T | 0.78 | 0.71 (0.61-0.83) | 8.57E-06 | 0.805 | 0.904 | 0.31 | 1.07E-04 | 0.311 | 0.07 | 5.37E-05 | 3.94E-02 |
| rs162278 | 19 | 53030170 | T | A | 0.79 | 0.71 (0.61-0.83) | 7.44E-06 | 0.779 | 0.907 | 0.3 | 8.29E-05 | 0.301 | 0.07 | 4.17E-05 | 3.77E-02 |
| rs162277 | 19 | 53030198 | C | G | 0.79 | 0.71 (0.61-0.83) | 6.95E-06 | 0.795 | 0.918 | 0.3 | 8.39E-05 | 0.312 | 0.06 | 4.21E-05 | 3.53E-02 |
| rs441850 | 19 | 53032790 | A | G | 0.78 | 0.74 (0.65-0.85) | 1.25E-05 | 0.812 | 0.967 | 0.28 | 1.14E-04 | 0.315 | 0.08 | 5.84E-05 | 3.57E-02 |
| rs413751 | 19 | 53032997 | T | A | 0.78 | 0.74 (0.65-0.85) | 1.39E-05 | 0.886 | 0.992 | 0.3 | 1.48E-04 | 0.348 | 0.07 | 6.42E-05 | 3.57E-02 |
| rs440545 | 19 | 53033037 | G | A | 0.78 | 0.74 (0.65-0.85) | 1.38E-05 | 0.869 | 0.978 | 0.3 | 1.44E-04 | 0.337 | 0.07 | 6.39E-05 | 3.55E-02 |
| rs429367 | 19 | 53033052 | T | C | 0.78 | 0.74 (0.65-0.85) | 1.38E-05 | 0.885 | 0.99 | 0.3 | 1.50E-04 | 0.348 | 0.07 | 6.40E-05 | 3.55E-02 |
| 19:53033160:ATATA | 19 | 53033160 | R | D | 0.79 | 0.72 (0.62-0.83) | 1.23E-05 | 0.934 | 0.99 | 0.33 | 1.52E-04 | 0.363 | 0.07 | 2.19E-02 | 7.55E-05 |
| rs410046 | 19 | 53033221 | C | T | 0.78 | 0.74 (0.65-0.85) | 1.39E-05 | 0.855 | 0.964 | 0.3 | 1.40E-04 | 0.326 | 0.08 | 6.42E-05 | 3.55E-02 |
| rs688646 | 19 | 53033898 | G | A | 0.78 | 0.74 (0.65-0.85) | 1.38E-05 | 0.851 | 0.965 | 0.3 | 1.38E-04 | 0.327 | 0.08 | 6.31E-05 | 3.52E-02 |
| rs449671 | 19 | 53033963 | C | G | 0.78 | 0.74 (0.65-0.85) | 1.37E-05 | 0.82 | 0.944 | 0.3 | 1.26E-04 | 0.307 | 0.08 | 6.29E-05 | 3.51E-02 |
| rs445500 | 19 | 53034112 | C | G | 0.78 | 0.74 (0.65-0.85) | 1.36E-05 | 0.855 | 0.97 | 0.3 | 1.39E-04 | 0.331 | 0.08 | 6.28E-05 | 3.49E-02 |
| rs434709 | 19 | 53034275 | G | T | 0.78 | 0.74 (0.65-0.85) | 1.36E-05 | 0.856 | 0.97 | 0.3 | 1.39E-04 | 0.331 | 0.07 | 6.23E-05 | 3.46E-02 |
| rs439618 | 19 | 53035300 | G | A | 0.78 | 0.74 (0.65-0.85) | 1.35E-05 | 0.84 | 0.956 | 0.3 | 1.34E-04 | 0.32 | 0.08 | 6.36E-05 | 3.44E-02 |
| rs400709 | 19 | 53035439 | T | A | 0.78 | 0.74 (0.65-0.85) | 1.35E-05 | 0.84 | 0.956 | 0.3 | 1.34E-04 | 0.321 | 0.08 | 7.22E-05 | 3.29E-02 |
| rs396663 | 19 | 53035704 | A | C | 0.78 | 0.74 (0.65-0.85) | 1.38E-05 | 0.841 | 0.952 | 0.3 | 1.41E-04 | 0.321 | 0.08 | 7.20E-05 | 3.32E-02 |
| rs422195 | 19 | 53035794 | G | C | 0.78 | 0.74 (0.65-0.85) | 1.30E-05 | 0.837 | 0.955 | 0.3 | 1.32E-04 | 0.322 | 0.08 | 6.66E-05 | 3.29E-02 |
| rs417599 | 19 | 53036339 | G | A | 0.78 | 0.74 (0.65-0.85) | 1.35E-05 | 0.829 | 0.948 | 0.3 | 1.30E-04 | 0.314 | 0.08 | 7.21E-05 | 3.30E-02 |
| rs413814 | 19 | 53036503 | C | G | 0.78 | 0.74 (0.65-0.85) | 1.22E-05 | 0.779 | 0.952 | 0.27 | 1.05E-04 | 0.302 | 0.08 | 6.57E-05 | 3.29E-02 |
| rs2608522 | 19 | 53036628 | C | T | 0.78 | 0.74 (0.65-0.85) | 1.35E-05 | 0.835 | 0.951 | 0.3 | 1.33E-04 | 0.318 | 0.08 | 7.21E-05 | 3.29E-02 |
| rs4802972 | 19 | 53036927 | T | G | 0.78 | 0.74 (0.65-0.85) | 1.35E-05 | 0.832 | 0.95 | 0.3 | 1.32E-04 | 0.316 | 0.08 | 6.35E-05 | 3.44E-02 |
| rs376595 | 19 | 53037612 | C | G | 0.78 | 0.74 (0.65-0.85) | 1.35E-05 | 0.839 | 0.953 | 0.3 | 1.33E-04 | 0.319 | 0.08 | 7.22E-05 | 3.29E-02 |
| rs369806 | 19 | 53038084 | G | C | 0.8 | 0.72 (0.62-0.84) | 1.75E-05 | 0.712 | 0.836 | 0.29 | 7.10E-05 | 0.173 | 0.15 | 1.63E-04 | 2.74E-02 |
| rs642493 | 19 | 53038498 | T | G | 0.77 | 0.75 (0.65-0.86) | 3.51E-05 | 0.696 | 0.841 | 0.3 | 2.02E-04 | 0.217 | 0.12 | 1.19E-04 | 6.03E-02 |
| rs656541 | 19 | 53039361 | T | A | 0.77 | 0.75 (0.66-0.86) | 3.87E-05 | 0.707 | 0.866 | 0.29 | 2.26E-04 | 0.23 | 0.11 | 1.36E-04 | 5.97E-02 |
| rs668882 | 19 | 53039793 | T | G | 0.77 | 0.75 (0.66-0.86) | 3.88E-05 | 0.671 | 0.83 | 0.29 | 2.05E-04 | 0.204 | 0.12 | 1.37E-04 | 5.97E-02 |
| rs2925976 | 19 | 53039893 | T | C | 0.77 | 0.75 (0.66-0.86) | 3.76E-05 | 0.674 | 0.832 | 0.29 | 2.02E-04 | 0.206 | 0.12 | 1.32E-04 | 6.00E-02 |
| rs2615586 | 19 | 53039961 | T | G | 0.77 | 0.75 (0.65-0.86) | 3.78E-05 | 0.669 | 0.844 | 0.28 | 1.93E-04 | 0.205 | 0.12 | 1.27E-04 | 6.14E-02 |
| rs2615585 | 19 | 53039978 | A | C | 0.77 | 0.75 (0.65-0.86) | 4.92E-05 | 0.689 | 0.868 | 0.28 | 2.56E-04 | 0.219 | 0.12 | 1.58E-04 | 6.78E-02 |
| rs2633514 | 19 | 53039980 | A | G | 0.77 | 0.75 (0.65-0.86) | 4.93E-05 | 0.687 | 0.866 | 0.28 | 2.52E-04 | 0.217 | 0.12 | 1.58E-04 | 6.79E-02 |
| rs2633513 | 19 | 53040022 | G | C | 0.77 | 0.75 (0.65-0.86) | 3.82E-05 | 0.676 | 0.869 | 0.27 | 2.05E-04 | 0.221 | 0.11 | 1.28E-04 | 6.11E-02 |
| rs2608527 | 19 | 53040100 | G | A | 0.76 | 0.75 (0.66-0.86) | 3.69E-05 | 0.607 | 0.811 | 0.27 | 1.63E-04 | 0.178 | 0.13 | 1.33E-04 | 6.16E-02 |
| 19:53040255:C_CAT | 19 | 53040255 | R | I | 0.78 | 0.74 (0.64-0.85) | 4.69E-05 | 0.636 | 0.832 | 0.28 | 2.17E-04 | 0.193 | 0.13 | 5.96E-02 | 1.83E-04 |
| 19:53040439:C_CA | 19 | 53040439 | R | I | 0.76 | 0.74 (0.64-0.86) | 4.42E-05 | 0.574 | 0.91 | 0.2 | 1.70E-04 | 0.212 | 0.11 | 5.90E-02 | 1.83E-04 |
| rs536063 | 19 | 53040450 | C | T | 0.77 | 0.75 (0.66-0.86) | 4.11E-05 | 0.665 | 0.82 | 0.29 | 2.06E-04 | 0.196 | 0.13 | 6.07E-02 | 1.53E-04 |
| rs532449 | 19 | 53040841 | G | A | 0.77 | 0.76 (0.66-0.86) | 4.16E-05 | 0.652 | 0.814 | 0.29 | 2.06E-04 | 0.191 | 0.13 | 1.59E-04 | 6.00E-02 |
| rs685200 | 19 | 53041107 | T | C | 0.77 | 0.76 (0.66-0.86) | 4.17E-05 | 0.652 | 0.815 | 0.29 | 2.07E-04 | 0.192 | 0.13 | 1.60E-04 | 5.99E-02 |
| rs529698 | 19 | 53041119 | A | C | 0.77 | 0.76 (0.66-0.86) | 4.18E-05 | 0.652 | 0.816 | 0.29 | 2.08E-04 | 0.192 | 0.13 | 1.61E-04 | 6.00E-02 |
| 19:53041229:G_GA | 19 | 53041229 | R | I | 0.77 | 0.75 (0.66-0.86) | 4.92E-05 | 0.64 | 0.796 | 0.29 | 2.20E-04 | 0.176 | 0.14 | 6.75E-02 | 1.75E-04 |
| 19:53041260:TTTAA | 19 | 53041260 | R | D | 0.77 | 0.75 (0.66-0.86) | 4.28E-05 | 0.651 | 0.808 | 0.29 | 2.08E-04 | 0.188 | 0.13 | 6.20E-02 | 1.66E-04 |
| rs2633502 | 19 | 53041385 | T | A | 0.77 | 0.76 (0.66-0.86) | 4.19E-05 | 0.65 | 0.812 | 0.29 | 2.05E-04 | 0.189 | 0.13 | 1.62E-04 | 5.99E-02 |
| rs12976905 | 19 | 53041749 | C | T | 0.8 | 0.73 (0.62-0.85) | 6.36E-05 | 0.694 | 0.866 | 0.29 | 3.38E-04 | 0.223 | 0.12 | 1.26E-04 | 9.66E-02 |
| rs2617795 | 19 | 53041995 | C | T | 0.76 | 0.76 (0.66-0.87) | 5.69E-05 | 0.633 | 0.823 | 0.28 | 2.60E-04 | 0.188 | 0.13 | 2.29E-04 | 6.93E-02 |
| rs92520 | 19 | 53042265 | C | T | 0.78 | 0.75 (0.65-0.86) | 6.42E-05 | 0.552 | 0.715 | 0.3 | 2.41E-04 | 0.133 | 0.15 | 1.40E-04 | 1.14E-01 |
| rs368922 | 19 | 53042477 | C | G | 0.77 | 0.76 (0.67-0.87) | 5.64E-05 | 0.631 | 0.8 | 0.29 | 2.50E-04 | 0.176 | 0.14 | 2.26E-04 | 6.72E-02 |
| rs402737 | 19 | 53042568 | C | T | 0.77 | 0.76 (0.67-0.87) | 5.64E-05 | 0.627 | 0.798 | 0.29 | 2.48E-04 | 0.174 | 0.14 | 2.26E-04 | 6.71E-02 |
| rs434788 | 19 | 53042715 | T | C | 0.77 | 0.76 (0.67-0.87) | 5.64E-05 | 0.627 | 0.798 | 0.29 | 2.48E-04 | 0.174 | 0.14 | 2.26E-04 | 6.71E-02 |
| rs606521 | 19 | 53043029 | C | A | 0.77 | 0.76 (0.66-0.87) | 5.85E-05 | 0.626 | 0.79 | 0.29 | 2.59E-04 | 0.173 | 0.14 | 2.26E-04 | 7.14E-02 |
| rs606538 | 19 | 53043039 | C | T | 0.77 | 0.76 (0.66-0.87) | 5.83E-05 | 0.623 | 0.79 | 0.29 | 2.54E-04 | 0.171 | 0.14 | 2.23E-04 | 7.15E-02 |
| rs606914 | 19 | 53043092 | C | T | 0.77 | 0.76 (0.67-0.87) | 5.82E-05 | 0.622 | 0.789 | 0.29 | 2.54E-04 | 0.171 | 0.14 | 2.33E-04 | 6.79E-02 |
| rs607477 | 19 | 53043275 | T | C | 0.77 | 0.76 (0.67-0.87) | 5.78E-05 | 0.628 | 0.795 | 0.29 | 2.57E-04 | 0.175 | 0.14 | 2.34E-04 | 6.74E-02 |
| rs10411295 | 19 | 54218314 | G | C | 0.7 | 1.3 (1.13-1.49) | 2.27E-04 | 0.523 | 0.531 | 0.3 | 5.01E-04 | 0.043 | 0.25 | 2.54E-03 | 2.28E-02 |
| rs8105296 | 19 | 54218388 | G | C | 0.72 | 1.29 (1.13-1.48) | 2.15E-04 | 0.574 | 0.575 | 0.3 | 5.58E-04 | 0.061 | 0.23 | 3.45E-03 | 1.81E-02 |
| rs4803167 | 19 | 54219395 | A | G | 0.7 | 1.29 (1.12-1.48) | 3.41E-04 | 0.554 | 0.558 | 0.28 | 8.37E-04 | 0.065 | 0.23 | 4.75E-03 | 2.12E-02 |
| rs730451 | 19 | 54221367 | A | G | 0.72 | 1.29 (1.13-1.48) | 1.55E-04 | 0.535 | 0.44 | 0.1 | 2.96E-04 | 0.105 | 0.13 | 1.98E-03 | 2.16E-02 |
| rs9304754 | 19 | 54222824 | C | T | 0.73 | 1.31 (1.15-1.5) | 4.33E-05 | 0.485 | 0.43 | 0.11 | 9.21E-05 | 0.086 | 0.12 | 7.45E-04 | 1.61E-02 |
| rs2217653 | 19 | 54223164 | T | C | 0.73 | 1.31 (1.15-1.5) | 4.30E-05 | 0.489 | 0.431 | 0.11 | 9.24E-05 | 0.087 | 0.12 | 7.41E-04 | 1.61E-02 |
| rs12976702 | 19 | 54224716 | C | T | 0.71 | 1.31 (1.14-1.51) | 1.12E-04 | 0.57 | 0.441 | 0.09 | 2.56E-04 | 0.122 | 0.11 | 2.05E-03 | 1.90E-02 |
| rs12975348 | 19 | 54224725 | C | G | 0.71 | 1.31 (1.14-1.51) | 1.12E-04 | 0.572 | 0.441 | 0.09 | 2.59E-04 | 0.123 | 0.11 | 2.05E-03 | 1.90E-02 |
| rs6509809 | 19 | 54225805 | A | G | 0.7 | 1.31 (1.14-1.51) | 1.12E-04 | 0.59 | 0.445 | 0.09 | 2.71E-04 | 0.129 | 0.1 | 1.53E-03 | 2.35E-02 |
| rs6509810 | 19 | 54226158 | T | C | 0.71 | 1.34 (1.16-1.54) | 6.22E-05 | 0.761 | 0.467 | 0.08 | 2.55E-04 | 0.188 | 0.06 | 6.34E-04 | 2.82E-02 |
| rs7248768 | 19 | 54226463 | G | T | 0.72 | 1.34 (1.16-1.54) | 7.78E-05 | 0.831 | 0.487 | 0.08 | 3.60E-04 | 0.208 | 0.06 | 7.65E-04 | 3.18E-02 |
| rs77893935 | 19 | 55505289 | G | C | 0.97 | 2.22 (1.52-3.23) | 9.84E-06 | 0.615 | 0.615 | 0.61 | 8.08E-05 | 0.001 | 0.34 | 2.84E-02 | 8.65E-05 |
| rs149737317 | 19 | 55510505 | G | A | 0.97 | 2.23 (1.51-3.29) | 1.60E-05 | 0.639 | 0.639 | 0.56 | 1.32E-04 | 0.006 | 0.31 | 2.24E-02 | 1.99E-04 |
| rs140469219 | 19 | 55517968 | T | A | 0.97 | 2.15 (1.47-3.13) | 2.31E-05 | 0.709 | 0.709 | 0.58 | 1.99E-04 | 0.012 | 0.3 | 2.37E-02 | 2.39E-04 |
| rs141745287 | 19 | 55524565 | A | G | 0.97 | 2.16 (1.48-3.15) | 1.92E-05 | 0.749 | 0.749 | 0.56 | 2.08E-04 | 0.038 | 0.25 | 1.86E-02 | 2.23E-04 |
| rs41275820 | 19 | 55525115 | G | A | 0.97 | 2.16 (1.48-3.14) | 1.93E-05 | 0.777 | 0.777 | 0.59 | 2.26E-04 | 0.045 | 0.24 | 2.23E-04 | 1.87E-02 |
| rs41275822 | 19 | 55525818 | C | T | 0.97 | 2.16 (1.48-3.14) | 2.02E-05 | 0.784 | 0.784 | 0.59 | 2.37E-04 | 0.047 | 0.24 | 2.23E-04 | 1.86E-02 |
| rs141784988 | 19 | 55528486 | G | A | 0.97 | 2.16 (1.48-3.14) | 1.96E-05 | 0.795 | 0.795 | 0.61 | 2.39E-04 | 0.051 | 0.23 | 1.82E-02 | 2.20E-04 |
| rs143750748 | 19 | 55528638 | C | T | 0.97 | 2.16 (1.48-3.14) | 1.96E-05 | 0.789 | 0.789 | 0.6 | 2.35E-04 | 0.049 | 0.23 | 1.82E-02 | 2.20E-04 |
| rs147804806 | 19 | 55530470 | T | C | 0.97 | 2.16 (1.48-3.14) | 1.96E-05 | 0.802 | 0.802 | 0.62 | 2.44E-04 | 0.054 | 0.23 | 1.81E-02 | 2.19E-04 |
| rs112822051 | 19 | 55547043 | C | T | 0.97 | 2.56 (1.67-3.93) | 3.24E-06 | 0.914 | 0.914 | 0.72 | 1.62E-04 | 0.168 | 0.1 | 1.71E-02 | 5.36E-05 |
| rs111687779 | 19 | 55547050 | G | A | 0.97 | 2.77 (1.73-4.43) | 4.34E-06 | 0.928 | 0.928 | 0.77 | 1.77E-04 | 0.157 | 0.12 | 1.77E-02 | 7.72E-05 |
| rs1654434 | 19 | 55550697 | C | T | 0.97 | 2.27 (1.55-3.34) | 7.65E-06 | 0.941 | 0.941 | 0.73 | 2.55E-04 | 0.161 | 0.12 | 7.43E-05 | 2.55E-02 |
| rs1671210 | 19 | 55551077 | G | C | 0.97 | 2.27 (1.55-3.34) | 7.67E-06 | 0.942 | 0.942 | 0.48 | 2.57E-04 | 0.231 | 0.04 | 7.49E-05 | 2.58E-02 |
| rs1654438 | 19 | 55551662 | G | A | 0.97 | 2.27 (1.55-3.34) | 7.70E-06 | 0.944 | 0.944 | 0.76 | 2.58E-04 | 0.232 | 0.04 | 7.54E-05 | 2.60E-02 |
| rs61009189 | 19 | 55551801 | G | C | 0.97 | 2.27 (1.55-3.34) | 7.76E-06 | 0.944 | 0.944 | 0.74 | 2.60E-04 | 0.232 | 0.04 | 7.64E-05 | 2.63E-02 |
| rs142159649 | 19 | 55552745 | C | T | 0.97 | 2.27 (1.54-3.34) | 7.76E-06 | 0.95 | 0.95 | 0.99 | 2.57E-04 | 0.153 | 0.13 | 2.65E-02 | 7.67E-05 |
| rs1671221 | 19 | 55557185 | G | A | 0.97 | 2.32 (1.57-3.43) | 5.36E-06 | 0.971 | 0.971 | 0.85 | 2.02E-04 | 0.163 | 0.13 | 7.94E-05 | 2.41E-02 |
| rs117257957 | 19 | 55559078 | A | G | 0.97 | 2.34 (1.58-3.47) | 5.22E-06 | 0.963 | 0.963 | 0.85 | 1.97E-04 | 0.16 | 0.13 | 2.62E-02 | 7.74E-05 |
| rs139915676 | 19 | 55561071 | C | T | 0.97 | 2.34 (1.58-3.48) | 5.41E-06 | 0.961 | 0.961 | 0.84 | 1.63E-04 | 0.135 | 0.16 | 2.72E-02 | 7.62E-05 |
| rs113118767 | 20 | 19499434 | T | A | 0.99 | 4.02 (1.99-8.13) | 8.97E-06 | 0.026 | 0.159 | 0.29 | 1.29E-01 | 0.868 | 0 | 4.94E-03 | 3.72E-03 |
| rs6071641 | 20 | 37677661 | T | A | 0.58 | 1.32 (1.17-1.5) | 6.44E-06 | 0.535 | 0.535 | 0.98 | 5.63E-04 | 0.563 | 0.03 | 5.13E-03 | 4.03E-04 |
| rs200218903 | 20 | 37677676 | R | D | 0.59 | 1.31 (1.16-1.48) | 9.47E-06 | 0.876 | 0.876 | 0.95 | 1.44E-04 | 0.121 | 0.17 | 7.96E-04 | 3.04E-03 |
| 20:37677898:TA_T | 20 | 37677898 | R | D | 0.57 | 1.3 (1.16-1.46) | 7.16E-06 | 0.83 | 0.83 | 0.98 | 2.49E-04 | 0.313 | 0.09 | 4.57E-04 | 3.97E-03 |
| rs742276 | 20 | 37679849 | G | A | 0.56 | 1.3 (1.16-1.45) | 8.74E-06 | 0.909 | 0.909 | 0.98 | 2.28E-04 | 0.245 | 0.11 | 4.85E-03 | 4.54E-04 |
| rs742274 | 20 | 37680944 | T | C | 0.57 | 1.28 (1.14-1.44) | 2.57E-05 | 0.976 | 0.976 | 0.93 | 4.12E-04 | 0.175 | 0.16 | 1.16E-02 | 5.11E-04 |
| rs4812357 | 20 | 37683071 | T | C | 0.56 | 1.3 (1.16-1.46) | 7.69E-06 | 0.885 | 0.885 | 0.99 | 2.25E-04 | 0.267 | 0.1 | 4.67E-03 | 4.33E-04 |
| rs7268772 | 20 | 37684501 | A | G | 0.56 | 1.3 (1.16-1.46) | 7.05E-06 | 0.856 | 0.856 | 0.91 | 2.31E-04 | 0.318 | 0.09 | 5.47E-03 | 3.50E-04 |
| rs1569538 | 20 | 37685216 | G | A | 0.56 | 1.29 (1.15-1.45) | 1.03E-05 | 0.863 | 0.863 | 0.9 | 2.94E-04 | 0.308 | 0.09 | 7.48E-03 | 3.44E-04 |
| rs6124105 | 20 | 37686081 | G | A | 0.56 | 1.29 (1.15-1.45) | 1.18E-05 | 0.803 | 0.803 | 0.87 | 3.89E-04 | 0.357 | 0.08 | 7.68E-03 | 4.14E-04 |
| rs6513588 | 20 | 37686281 | T | C | 0.56 | 1.29 (1.15-1.45) | 1.19E-05 | 0.831 | 0.831 | 0.88 | 3.56E-04 | 0.331 | 0.09 | 7.73E-03 | 4.15E-04 |
| rs6513589 | 20 | 37686585 | A | G | 0.56 | 1.29 (1.15-1.45) | 1.20E-05 | 0.849 | 0.849 | 0.85 | 3.46E-04 | 0.321 | 0.09 | 7.78E-03 | 4.15E-04 |
| rs6129163 | 20 | 37687130 | A | G | 0.57 | 1.28 (1.14-1.43) | 3.01E-05 | 0.762 | 0.762 | 0.93 | 8.05E-04 | 0.352 | 0.1 | 1.43E-02 | 6.98E-04 |
| 20:37687237:A_AG | 20 | 37687237 | R | I | 0.57 | 1.27 (1.13-1.43) | 3.75E-05 | 0.879 | 0.879 | 0.87 | 7.10E-04 | 0.262 | 0.13 | 9.49E-04 | 1.03E-02 |
| rs714108 | 20 | 37687838 | G | A | 0.56 | 1.29 (1.15-1.45) | 1.20E-05 | 0.833 | 0.833 | 0.85 | 3.64E-04 | 0.332 | 0.09 | 7.96E-03 | 3.91E-04 |
| rs6129164 | 20 | 37688347 | T | C | 0.56 | 1.29 (1.15-1.45) | 1.21E-05 | 0.834 | 0.834 | 0.86 | 3.63E-04 | 0.328 | 0.09 | 8.02E-03 | 3.92E-04 |
| rs6129165 | 20 | 37688553 | C | T | 0.56 | 1.29 (1.15-1.45) | 1.22E-05 | 0.855 | 0.855 | 0.91 | 3.47E-04 | 0.298 | 0.1 | 8.06E-03 | 3.93E-04 |
| rs6129166 | 20 | 37688825 | T | C | 0.56 | 1.29 (1.15-1.45) | 1.24E-05 | 0.845 | 0.845 | 0.86 | 3.63E-04 | 0.319 | 0.09 | 8.15E-03 | 3.94E-04 |
| rs6129167 | 20 | 37689228 | T | C | 0.56 | 1.29 (1.15-1.45) | 1.26E-05 | 0.845 | 0.845 | 0.86 | 3.67E-04 | 0.318 | 0.09 | 8.24E-03 | 3.95E-04 |
| rs4812360 | 20 | 37689259 | A | G | 0.56 | 1.29 (1.15-1.45) | 1.28E-05 | 0.844 | 0.844 | 0.87 | 3.73E-04 | 0.319 | 0.09 | 8.28E-03 | 4.00E-04 |
| rs6129168 | 20 | 37689360 | T | C | 0.56 | 1.29 (1.15-1.45) | 1.27E-05 | 0.854 | 0.854 | 0.87 | 3.49E-04 | 0.305 | 0.1 | 7.96E-03 | 4.13E-04 |
| rs926392 | 20 | 37690464 | A | G | 0.56 | 1.29 (1.15-1.44) | 1.38E-05 | 0.755 | 0.755 | 0.92 | 4.45E-04 | 0.369 | 0.09 | 8.63E-03 | 4.18E-04 |
| rs926391 | 20 | 37690485 | A | G | 0.56 | 1.29 (1.15-1.44) | 1.40E-05 | 0.768 | 0.768 | 0.92 | 4.35E-04 | 0.354 | 0.09 | 8.67E-03 | 4.24E-04 |
| rs2867898 | 20 | 37690952 | G | A | 0.56 | 1.29 (1.15-1.44) | 1.58E-05 | 0.821 | 0.821 | 0.88 | 4.18E-04 | 0.318 | 0.1 | 8.89E-03 | 4.78E-04 |
| rs6513593 | 20 | 37691392 | G | A | 0.56 | 1.28 (1.14-1.44) | 1.83E-05 | 0.815 | 0.815 | 0.88 | 4.71E-04 | 0.319 | 0.1 | 1.00E-02 | 4.97E-04 |
| rs200132136 | 20 | 37692035 | R | D | 0.58 | 1.29 (1.14-1.46) | 3.52E-05 | 0.753 | 0.753 | 0.86 | 8.69E-04 | 0.366 | 0.1 | 3.48E-04 | 2.01E-02 |
| rs6028258 | 20 | 37692844 | T | A | 0.56 | 1.28 (1.14-1.44) | 2.02E-05 | 0.822 | 0.822 | 0.87 | 4.83E-04 | 0.309 | 0.11 | 9.14E-03 | 6.48E-04 |
| rs6124106 | 20 | 37693042 | G | A | 0.56 | 1.28 (1.14-1.44) | 2.12E-05 | 0.789 | 0.789 | 0.85 | 5.53E-04 | 0.341 | 0.1 | 9.30E-03 | 6.75E-04 |
| rs6027867 | 20 | 59434375 | G | T | 0.82 | 1.35 (1.16-1.58) | 8.68E-05 | 0.222 | 0.26 | 0.13 | 1.16E-02 | 0.759 | 0 | 5.55E-03 | 3.67E-02 |
| rs912903 | 20 | 59434399 | T | C | 0.82 | 1.35 (1.16-1.58) | 8.72E-05 | 0.221 | 0.259 | 0.14 | 1.17E-02 | 0.759 | 0 | 5.55E-03 | 3.67E-02 |
| rs6071381 | 20 | 59434798 | T | G | 0.82 | 1.35 (1.16-1.58) | 8.93E-05 | 0.22 | 0.258 | 0.14 | 1.19E-02 | 0.758 | 0 | 5.56E-03 | 3.69E-02 |
| rs6027868 | 20 | 59435257 | A | C | 0.82 | 1.35 (1.16-1.58) | 9.35E-05 | 0.233 | 0.265 | 0.17 | 1.16E-02 | 0.786 | 0 | 5.67E-03 | 3.79E-02 |
| rs6027869 | 20 | 59435312 | T | C | 0.82 | 1.35 (1.16-1.58) | 9.46E-05 | 0.217 | 0.256 | 0.14 | 1.24E-02 | 0.756 | 0 | 5.70E-03 | 3.82E-02 |
| rs6015867 | 20 | 59435732 | G | A | 0.82 | 1.35 (1.16-1.58) | 9.69E-05 | 0.221 | 0.26 | 0.14 | 1.21E-02 | 0.764 | 0 | 5.76E-03 | 3.88E-02 |
| rs6027870 | 20 | 59435791 | G | C | 0.82 | 1.35 (1.16-1.58) | 9.80E-05 | 0.217 | 0.256 | 0.14 | 1.26E-02 | 0.755 | 0 | 5.78E-03 | 3.90E-02 |
| rs6027871 | 20 | 59435967 | G | A | 0.82 | 1.35 (1.16-1.58) | 9.49E-05 | 0.219 | 0.259 | 0.13 | 1.22E-02 | 0.754 | 0 | 5.69E-03 | 3.66E-02 |
| rs16985040 | 20 | 59436894 | C | T | 0.82 | 1.35 (1.16-1.57) | 1.07E-04 | 0.215 | 0.255 | 0.14 | 1.32E-02 | 0.753 | 0 | 5.98E-03 | 4.10E-02 |
| rs2038892 | 20 | 59438080 | T | C | 0.82 | 1.34 (1.15-1.57) | 1.36E-04 | 0.214 | 0.254 | 0.14 | 1.51E-02 | 0.748 | 0 | 6.61E-03 | 4.73E-02 |
| rs6093016 | 20 | 59438911 | C | G | 0.79 | 1.33 (1.15-1.54) | 1.36E-04 | 0.244 | 0.286 | 0.2 | 1.05E-02 | 0.825 | 0.01 | 6.82E-03 | 5.97E-02 |
| rs6027876 | 20 | 59440363 | C | T | 0.82 | 1.34 (1.15-1.56) | 1.55E-04 | 0.211 | 0.254 | 0.15 | 1.59E-02 | 0.751 | 0 | 7.09E-03 | 5.15E-02 |
| rs6027877 | 20 | 59440390 | G | A | 0.82 | 1.34 (1.15-1.56) | 1.57E-04 | 0.211 | 0.254 | 0.15 | 1.60E-02 | 0.752 | 0 | 7.12E-03 | 5.19E-02 |
| rs59603367 | 21 | 42433821 | T | C | 0.95 | 0.57 (0.44-0.74) | 3.52E-05 | 0.497 | 0.462 | 0.18 | 5.98E-05 | 0.048 | 0.18 | 1.31E-02 | 9.58E-04 |
| rs66931916 | 21 | 42436854 | A | G | 0.95 | 0.57 (0.44-0.75) | 3.83E-05 | 0.433 | 0.421 | 0.15 | 5.14E-05 | 0.043 | 0.18 | 1.67E-02 | 8.14E-04 |
| rs746288 | 21 | 42437924 | T | C | 0.95 | 0.57 (0.44-0.75) | 3.97E-05 | 0.537 | 0.46 | 0.13 | 7.43E-05 | 0.079 | 0.14 | 1.76E-02 | 7.98E-04 |
| rs35828049 | 21 | 42440044 | A | T | 0.95 | 0.58 (0.44-0.75) | 4.55E-05 | 0.435 | 0.422 | 0.15 | 6.01E-05 | 0.043 | 0.18 | 2.09E-02 | 7.47E-04 |

^1^SNP=single nucleotide polymorphism; ^2^CHR=chromosome; ^3^BP=chromosomal position in base pairs; ^4^OR=odds ratio, 95% CI=95% confidence interval; ^5^Pfixed=fixed effects P; ^6^Prandom=random effects P; ^7^Phet=P value for heterogeneity of effect across studies; ^8^CCFR=Colon Cancer Family Registry; ^9^BCFR= Breast Cancer Family Registry.
