# Supplementary material for: Genetic susceptibility markers for a breast-colorectal cancer phenotype: Exploratory results from genome-wide association studies
Source: PLoS One. 2018 Apr 26;13(4):e0196245. doi: 10.1371/journal.pone.0196245 (PMC5919670; doi:10.1371/journal.pone.0196245)
Supplement: S2 Table — (DOCX) [file pone.0196245.s006.docx]

**S2 Table**. **Odds ratio (OR) estimates with 95% confidence interval (CI) for association of selected colorectal cancer** **GWAS SNPs with the breast-colorectal cancer phenotype**. (Selected GWAS SNPs are SNPs found to be associated with colorectal cancer among non-Hispanic whites in published GWASs [39 loci from 10 GWAS studies in EUR, accessed from Haploreg V3, on 03/20/2018]).

| **CHR^1^** | **BP**^2^ | **SNP**^3^ | **Coded allele** | **Alternate allele** | **Coded allele frequency** | **OR (95% CI)**^4^ | ***P*** | **Within or nearby gene**^5^ | |
| --- | --- | --- | --- | --- | --- | --- | --- | --- | --- |
| 18 | 18:46453463 | rs4939827 | T | C | 0.54 | 1.24 (1.11-1.39) | 0.0002 | SMAD7 |  |
| 11 | 11:111171709 | rs3802842 | A | C | 0.7 | 0.85 (0.76-0.96) | 0.01 | C11orf93 |  |
| 15 | 15:32994756 | rs4779584 | C | T | 0.8 | 0.84 (0.73-0.97) | 0.015 | 5.5kb 3' of SCG5 | |
| 12 | 12:51155663 | rs11169552 | C | T | 0.72 | 1.16 (1.02-1.32) | 0.023 | 1.8kb 5' of ATF1 | |
| 6 | 6:36622900 | rs1321311 | C | A | 0.74 | 0.89 (0.78-1.01) | 0.068 | PI16 |  |
| 20 | 20:6699595 | rs4813802 | T | G | 0.65 | 0.9 (0.8-1.01) | 0.079 | 12kb 3' of RP5-859D4.3 | |
| 11 | 11:74345550 | rs3824999 | G | T | 0.53 | 1.19 (0.98-1.44) | 0.086 | POLD3 |  |
| 8 | 8:117630683 | rs16892766 | A | C | 0.92 | 0.84 (0.69-1.03) | 0.093 | 24kb 3' of EIF3H | |
| 4 | 4:94887031 | rs13130787 | T | C | 0.54 | 0.91 (0.81-1.02) | 0.116 | 136kb 3' of ATOH1 | |
| 10 | 10:8701219 | rs10795668 | G | A | 0.68 | 1.1 (0.97-1.24) | 0.137 | 2.4kb 5' of RN5S299 | |
| 8 | 8:130820039 | rs2128382 | C | T | 0.83 | 1.12 (0.96-1.31) | 0.146 | 21kb 5' of GSDMC | |
| 12 | 12:115891403 | rs7315438 | T | C | 0.56 | 1.09 (0.97-1.23) | 0.161 | 89kb 3' of RP11-116D17.1 | |
| 8 | 8:128413305 | rs6983267 | T | G | 0.48 | 0.92 (0.82-1.03) | 0.167 | RP11-382A18.1 |  |
| 16 | 16:68820946 | rs9929218 | G | A | 0.7 | 1.09 (0.95-1.24) | 0.214 | CDH1 |  |
| 20 | 20:6404281 | rs961253 | C | A | 0.65 | 0.93 (0.82-1.04) | 0.215 | 23kb 5' of RP11-199O14.1 | |
| 12 | 12:72414563 | rs10879357 | G | A | 0.62 | 0.93 (0.83-1.05) | 0.233 | TPH2 |  |
| 8 | 8:128424792 | rs7014346 | G | A | 0.62 | 0.93 (0.83-1.05) | 0.253 | RP11-382A18.1 |  |
| 14 | 14:59189361 | rs17094983 | G | A | 0.88 | 1.26 (0.84-1.89) | 0.259 | 49kb 3' of AL121819.1 | |
| 20 | 20:60921044 | rs4925386 | C | T | 0.71 | 1.07 (0.95-1.21) | 0.274 | LAMA5 |  |
| 14 | 14:54410919 | rs4444235 | T | C | 0.44 | 1.07 (0.95-1.2) | 0.279 | 4.2kb 3' of MIR5580 | |
| 1 | 1:162821291 | rs1912453 | G | A | 0.62 | 1.06 (0.94-1.19) | 0.316 | C1orf110 |  |
| 2 | 2:192587204 | rs11903757 | T | C | 0.85 | 0.93 (0.79-1.08) | 0.34 | 34kb 3' of NABP1 | |
| 3 | 3:23143047 | rs4591517 | C | T | 0.69 | 1.06 (0.94-1.2) | 0.367 | 94kb 3' of AC135966.1 | |
| 1 | 1:183081194 | rs10911251 | A | C | 0.56 | 1.05 (0.94-1.18) | 0.382 | LAMC1 |  |
| 19 | 19:33532300 | rs10411210 | C | T | 0.9 | 1.09 (0.9-1.32) | 0.395 | RHPN2 |  |
| 3 | 3:169492101 | rs10936599 | C | T | 0.75 | 1.06 (0.92-1.21) | 0.416 | MYNN |  |
| 9 | 9:96631134 | rs10114408 | A | T | 0.62 | 1.04 (0.93-1.17) | 0.458 | 11kb 3' of RP11-53B5.1 | |
| 7 | 7:25133849 | rs39453 | T | C | 0.65 | 0.96 (0.85-1.08) | 0.471 | 26kb 3' of CYCS | |
| 12 | 12:115116352 | rs59336 | A | T | 0.51 | 0.96 (0.85-1.08) | 0.487 | TBX3 |  |
| 3 | 3:47388947 | rs8180040 | T | A | 0.61 | 1.04 (0.93-1.17) | 0.490 | 640bp 3' of KLHL18 | |
| 4 | 4:67357454 | rs17730929 | A | C | 0.92 | 0.94 (0.76-1.16) | 0.541 | 215kb 3' of MIR1269A | |
| 5 | 5:108948937 | rs367615 | T | C | 0.82 | 1.03 (0.88-1.21) | 0.686 | 72kb 5' of AC012603.1 | |
| 1 | 1:222164948 | rs6687758 | A | G | 0.79 | 1.03 (0.89-1.18) | 0.701 | 6.6kb 3' of RP11-400N13.2 | |
| 8 | 8:96595736 | rs3104964 | A | G | 0.60 | 0.98 (0.87-1.1) | 0.708 | KB-1047C11.2 |  |
| 8 | 8:29344462 | rs12548021 | A | G | 0.62 | 0.99 (0.88-1.11) | 0.828 | 40kb 5' of RP11-486M23.1 | |
| 6 | 6:158435572 | rs9365723 | G | A | 0.62 | 1.01 (0.9-1.13) | 0.905 | SYNJ2 |  |
| 6 | 6:117819357 | rs2057314 | G | A | 0.49 | 1.01 (0.9-1.13) | 0.912 | GOPC |  |
| 1 | 1:222045446 | rs6691170 | G | T | 0.63 | 1.00 (0.89-1.13) | 0.936 | 8.9kb 3' of RP11-815M8.1 | |
| 12 | 12:4405389 | rs3217901 | A | G | 0.59 | 1 (0.89-1.12) | 0.967 | CCND2 |  |

^1^CHR=chromosome; ^2^BP=chromosomal position in base pairs; ^3^SNP=single nucleotide polymorphism; ^4^OR=odds ratio, 95% CI=95% confidence interval; ^5^from HaploReg
