# Supplementary material for: Genetic susceptibility markers for a breast-colorectal cancer phenotype: Exploratory results from genome-wide association studies
Source: PLoS One. 2018 Apr 26;13(4):e0196245. doi: 10.1371/journal.pone.0196245 (PMC5919670; doi:10.1371/journal.pone.0196245)
Supplement: S3 Table — (DOCX) [file pone.0196245.s007.docx]

**S3 Table.** **Odds ratio (OR) estimates with 95% confidence interval (CI) for association of selected breast cancer** **GWAS SNPs with the breast-colorectal cancer phenotype**. SNPs were selected from published GWASs if found to be associated with breast cancer among non-Hispanic whites.

| **CHR**^1^ | **BP**^2^ | **SNP**^3^ | **Coded allele** | **Alternate allele** | **Coded allele frequency** | **OR (95% CI)**^4^ | ***P*** | **Within or nearby gene**^5^ |
| --- | --- | --- | --- | --- | --- | --- | --- | --- |
| 9 | 9:22062134 | rs1011970 | G | T | 0.82 | 0.82 (0.71-0.95) | 0.007 | CDKN2B-AS1 |
| 10 | 10:123337335 | rs2981579 | G | A | 0.57 | 0.86 (0.77-0.96) | 0.01 | FGFR2 |
| 10 | 10:123352317 | rs2981582 | G | A | 0.59 | 0.86 (0.77-0.97) | 0.012 | FGFR2 |
| 10 | 10:5886734 | rs2380205 | C | T | 0.57 | 0.88 (0.79-0.99) | 0.027 | 2.6kb 5' of GDI2 |
| 16 | 16:80650805 | rs13329835 | A | G | 0.77 | 0.87 (0.76-0.99) | 0.035 | CDYL2 |
| 16 | 16:52635164 | rs3112612 | G | A | 0.57 | 0.89 (0.8-1) | 0.04 | RP11-297L17.1 |
| 10 | 10:64251977 | rs10822013 | T | C | 0.52 | 0.89 (0.79-1) | 0.04 | ZNF365 |
| 12 | 12:115836522 | rs1292011 | A | G | 0.59 | 1.12 (1-1.26) | 0.058 | 34kb 3' of RP11-116D17.1 |
| 16 | 16:53813367 | rs17817449 | T | G | 0.63 | 0.90 (0.8-1.01) | 0.063 | FTO |
| 22 | 22:40876234 | rs6001930 | T | C | 0.9 | 1.20 (0.99-1.46) | 0.068 | MKL1 |
| 8 | 8:128355618 | rs13281615 | A | G | 0.58 | 0.90 (0.8-1.01) | 0.071 | RP11-382A18.2 |
| 20 | 20:32588095 | rs2284378 | C | T | 0.68 | 0.90 (0.8-1.01) | 0.081 | RALY |
| 10 | 10:64278682 | rs10995190 | G | A | 0.85 | 0.87 (0.74-1.02) | 0.086 | ZNF365 |
| 11 | 11:129461171 | rs11820646 | C | T | 0.6 | 1.11 (0.99-1.24) | 0.086 | 139kb 3' of BARX2 |
| 5 | 5:56031884 | rs889312 | A | C | 0.72 | 0.90 (0.79-1.02) | 0.087 | 35kb 5' of AC008940.1 |
| 7 | 7:130653851 | rs2048672 | C | A | 0.71 | 1.12 (0.98-1.28) | 0.09 | AC058791.2 |
| 5 | 5:58184061 | rs10472076 | T | C | 0.63 | 0.91 (0.8-1.02) | 0.102 | 25kb 5' of CTD-2176I21.2 |
| 5 | 5:56023083 | rs16886165 | T | G | 0.84 | 0.89 (0.76-1.03) | 0.12 | 44kb 5' of AC008940.1 |
| 4 | 4:175846426 | rs6828523 | C | A | 0.88 | 1.15 (0.96-1.38) | 0.121 | ADAM29 |
| 12 | 12:28155080 | rs10771399 | A | G | 0.89 | 1.16 (0.96-1.4) | 0.124 | 29kb 5' of PTHLH |
| 19 | 19:52372976 | rs10411161 | C | T | 0.88 | 0.87 (0.74-1.04) | 0.13 | ZNF577 |
| 5 | 5:45285718 | rs981782 | A | C | 0.55 | 1.09 (0.97-1.22) | 0.13 | HCN1 |
| 8 | 8:29509616 | rs9693444 | C | A | 0.68 | 0.91 (0.81-1.03) | 0.134 | 22kb 3' of LINC00589 |
| 8 | 8:76417937 | rs2943559 | A | G | 0.92 | 0.86 (0.7-1.05) | 0.14 | HNF4G |
| 14 | 14:91841069 | rs941764 | A | G | 0.65 | 0.92 (0.82-1.03) | 0.162 | CCDC88C |
| 2 | 2:217905832 | rs13387042 | A | G | 0.52 | 1.08 (0.97-1.21) | 0.173 | 45kb 3' of RP11-574O16.1 |
| 2 | 2:218296508 | rs16857609 | C | T | 0.72 | 0.92 (0.81-1.04) | 0.181 | DIRC3 |
| 16 | 16:53855291 | rs11075995 | T | A | 0.76 | 0.91 (0.8-1.04) | 0.182 | FTO |
| 9 | 9:110306115 | rs10759243 | C | A | 0.72 | 0.92 (0.81-1.04) | 0.185 | 53kb 5' of KLF4 |
| 6 | 6:19443935 | rs16882214 | C | G | 0.85 | 0.89 (0.74-1.06) | 0.2 | 5.3kb 3' of RN5S205 |
| 2 | 2:174212894 | rs1550623 | A | G | 0.85 | 1.11 (0.94-1.29) | 0.209 | AC092573.3 |
| 15 | 15:48900586 | rs1876206 | T | C | 0.85 | 0.9 (0.76-1.07) | 0.22 | FBN1 |
| 5 | 5:158244083 | rs1432679 | T | C | 0.56 | 0.93 (0.83-1.05) | 0.238 | EBF1 |
| 10 | 10:22032942 | rs7072776 | G | A | 0.7 | 0.93 (0.82-1.05) | 0.246 | 382bp 3' of MLLT10 |
| 10 | 10:80841148 | rs704010 | C | T | 0.61 | 0.93 (0.83-1.05) | 0.253 | ZMIZ1 |
| 4 | 4:106084778 | rs9790517 | C | T | 0.79 | 0.92 (0.81-1.06) | 0.256 | TET2 |
| 13 | 13:95874956 | rs1926657 | C | T | 0.84 | 1.09 (0.94-1.27) | 0.26 | ABCC4 |
| 16 | 16:74472696 | rs10871290 | T | C | 0.64 | 1.07 (0.95-1.2) | 0.27 | 3.5kb 3' of RP11-252A24.5 |
| 19 | 19:18571141 | rs4808801 | A | G | 0.65 | 1.07 (0.95-1.21) | 0.271 | ELL |
| 10 | 10:123625190 | rs10510102 | T | C | 0.82 | 0.92 (0.8-1.07) | 0.29 | ATE1 |
| 14 | 14:37132769 | rs2236007 | G | A | 0.81 | 1.26 (0.81-1.96) | 0.31 | PAX9 |
| 5 | 5:44875005 | rs7716600 | C | A | 0.77 | 0.93 (0.82-1.07) | 0.31 | 54kb 3' of MRPS30 |
| 15 | 15:78269472 | rs12906542 | A | G | 0.79 | 0.63 (0.25-1.58) | 0.32 | 6.9kb 3' of TBC1D2B |
| 11 | 11:65583066 | rs3903072 | G | T | 0.67 | 0.91 (0.77-1.09) | 0.327 | 7.4kb 3' of CFL1 |
| 10 | 10:114773927 | rs7904519 | A | G | 0.53 | 0.94 (0.84-1.06) | 0.328 | TCF7L2 |
| 16 | 16:52586341 | rs3803662 | G | A | 0.72 | 0.94 (0.83-1.07) | 0.341 | RP11-297L17.1 |
| 17 | 17:48274291 | rs2075555 | G | T | 0.86 | 0.92 (0.77-1.1) | 0.37 | COL1A1 |
| 6 | 6:151948366 | rs2046210 | G | A | 0.65 | 0.95 (0.85-1.07) | 0.379 | 6kb 3' of CCDC170 |
| 1 | 1:204518842 | rs4245739 | A | C | 0.73 | 1.06 (0.93-1.2) | 0.386 | MDM4 |
| 5 | 5:44706498 | rs10941679 | A | G | 0.74 | 0.95 (0.83-1.08) | 0.411 | 5.6kb 5' of RP11-503D12.1 |
| 11 | 11:1909006 | rs3817198 | T | C | 0.68 | 1.05 (0.93-1.18) | 0.432 | LSP1 |
| 6 | 6:82193109 | rs17530068 | T | C | 0.77 | 0.95 (0.83-1.08) | 0.44 | 8kb 3' of FAM46A |
| 6 | 6:1318878 | rs11242675 | T | C | 0.64 | 1.05 (0.93-1.18) | 0.443 | 3.9kb 3' of FOXQ1 |
| 9 | 9:110888478 | rs865686 | T | G | 0.63 | 0.96 (0.85-1.07) | 0.451 | 126kb 3' of RP11-505C13.1 |
| 6 | 6:151987357 | rs9383938 | G | T | 0.91 | 1.08 (0.88-1.32) | 0.46 | ESR1 |
| 12 | 12:73989837 | rs1154865 | C | G | 0.77 | 1.05 (0.92-1.21) | 0.46 | RP11-314D7.4 |
| 21 | 21:31177511 | rs458685 | A | G | 0.84 | 1.06 (0.9-1.25) | 0.46 | GRIK1 |
| 8 | 8:129194641 | rs11780156 | C | T | 0.82 | 1.06 (0.91-1.22) | 0.467 | 32kb 3' of MIR1208 |
| 7 | 7:67059267 | rs10263639 | T | C | 0.85 | 1.06 (0.9-1.24) | 0.47 | 97kb 3' of RP4-736H5.3 |
| 14 | 14:68660428 | rs2588809 | C | T | 0.9 | 1.11 (0.82-1.5) | 0.499 | RAD51B |
| 3 | 3:30682939 | rs12493607 | G | C | 0.66 | 0.96 (0.85-1.08) | 0.501 | TGFBR2 |
| 8 | 8:128387852 | rs1562430 | T | C | 0.58 | 1.04 (0.93-1.17) | 0.51 | RP11-382A18.2 |
| 3 | 3:4742276 | rs6762644 | A | G | 0.61 | 0.96 (0.86-1.08) | 0.516 | ITPR1 |
| 2 | 2:19320803 | rs12710696 | C | T | 0.62 | 1.04 (0.93-1.17) | 0.517 | AC092594.1 |
| 1 | 1:10566215 | rs616488 | A | G | 0.67 | 1.04 (0.92-1.17) | 0.519 | PEX14 |
| 5 | 5:44662515 | rs4415084 | C | T | 0.58 | 0.96 (0.86-1.08) | 0.52 | 36kb 3' of RP11-503D12.1 |
| 12 | 12:96027759 | rs17356907 | A | G | 0.69 | 0.96 (0.84-1.09) | 0.53 | 16kb 3' of Y_RNA |
| 5 | 5:10467702 | rs1092913 | G | A | 0.89 | 1.08 (0.85-1.37) | 0.53 | ROPN1L |
| 2 | 2:172972971 | rs2016394 | G | A | 0.53 | 0.96 (0.86-1.08) | 0.531 | AC104801.1 |
| 6 | 6:151914113 | rs3757318 | G | A | 0.92 | 1.07 (0.86-1.33) | 0.538 | CCDC170 |
| 6 | 6:127600630 | rs2180341 | A | G | 0.75 | 1.04 (0.91-1.19) | 0.54 | RNF146 |
| 14 | 14:69034682 | rs999737 | C | T | 0.9 | 0.9 (0.62-1.29) | 0.56 | RAD51B |
| 5 | 5:163889280 | rs6556756 | T | G | 0.88 | 1.05 (0.89-1.25) | 0.56 | CTD-2313P7.1 |
| 19 | 19:17389704 | rs8170 | G | A | 0.81 | 1.04 (0.9-1.21) | 0.567 | BABAM1 |
| 11 | 11:69328764 | rs614367 | C | T | 0.88 | 0.82 (0.37-1.83) | 0.63 | 34kb 5' of AP000439.3 |
| 10 | 10:123093901 | rs11199914 | C | T | 0.68 | 0.97 (0.86-1.1) | 0.63 | 77kb 5' of 7SK |
| 19 | 19:44286513 | rs3760982 | G | A | 0.53 | 0.97 (0.87-1.09) | 0.648 | 1.1kb 5' of KCNN4 |
| 2 | 2:121245122 | rs4849887 | C | T | 0.9 | 1.05 (0.86-1.27) | 0.649 | 55kb 3' of AC073257.2 |
| 2 | 2:59499347 | rs10490113 | A | C | 0.89 | 1.04 (0.87-1.26) | 0.65 | AC007131.1 |
| 10 | 10:22315843 | rs11814448 | A | C | 0.98 | 1.1 (0.71-1.7) | 0.659 | 23kb 5' of DNAJC1 |
| 11 | 11:1941946 | rs909116 | T | C | 0.52 | 0.98 (0.87-1.09) | 0.66 | TNNT3 |
| 18 | 18:24570667 | rs1436904 | T | G | 0.6 | 1.02 (0.91-1.15) | 0.684 | CHST9-AS1 |
| 12 | 12:14413931 | rs12422552 | G | C | 0.73 | 0.97 (0.85-1.11) | 0.689 | 11kb 5' of U6 |
| 5 | 5:1279790 | rs10069690 | C | T | 0.74 | 1.03 (0.9-1.17) | 0.697 | TERT |
| 13 | 13:32972626 | rs11571833 | A | T | 0.99 | 0.88 (0.45-1.7) | 0.7 | BRCA2 |
| 8 | 8:76230301 | rs6472903 | T | G | 0.83 | 1.03 (0.88-1.2) | 0.721 | RP11-697M17.1 |
| 1 | 1:114448389 | rs11552449 | C | T | 0.84 | 0.97 (0.84-1.14) | 0.744 | DCLRE1B |
| 3 | 3:27416013 | rs4973768 | C | T | 0.51 | 1.02 (0.91-1.14) | 0.78 | SLC4A7 |
| 18 | 18:24337424 | rs527616 | G | C | 0.63 | 0.98 (0.85-1.13) | 0.785 | CHST9-AS1 |
| 1 | 1:202187176 | rs6678914 | G | A | 0.59 | 0.99 (0.88-1.11) | 0.798 | LGR6 |
| 1 | 1:121280613 | rs11249433 | A | G | 0.59 | 1.01 (0.91-1.13) | 0.808 | 28kb 5' of AL592494.4 |
| 7 | 7:144074929 | rs720475 | G | A | 0.75 | 1.02 (0.89-1.16) | 0.817 | ARHGEF5 |
| 6 | 6:13722523 | rs204247 | A | G | 0.57 | 1.01 (0.9-1.13) | 0.878 | 11kb 5' of RANBP9 |
| 5 | 5:58337481 | rs1353747 | T | G | 0.9 | 1.01 (0.83-1.23) | 0.882 | RP11-266N13.2 |
| 19 | 19:17392894 | rs8100241 | A | G | 0.52 | 0.99 (0.89-1.11) | 0.9 | ANKLE1 |
| 22 | 22:29621477 | rs132390 | T | C | 0.96 | 1.02 (0.7-1.5) | 0.905 | EMID1 |
| 17 | 17:53056471 | rs6504950 | G | A | 0.71 | 1.00 (0.88-1.14) | 0.947 | STXBP4 |
| 18 | 18:53664282 | rs1978503 | A | G | 0.81 | 1 (0.86-1.15) | 0.96 | 6.6kb 3' of AC006305.1 |
| 21 | 21:16520832 | rs283093 | G | A | 0.74 | 1.00 (0.88-1.14) | 0.993 | 81kb 3' of AF127577.12 |

^1^CHR=chromosome; ^2^BP=chromosomal position in base pairs; ^3^SNP=single nucleotide polymorphism; ^4^OR=odds ratio, 95% CI=95% confidence interval; ^5^from HaploReg
