# Supplementary material for: Genetic susceptibility markers for a breast-colorectal cancer phenotype: Exploratory results from genome-wide association studies
Source: PLoS One. 2018 Apr 26;13(4):e0196245. doi: 10.1371/journal.pone.0196245 (PMC5919670; doi:10.1371/journal.pone.0196245)
Supplement: S4 Table — (DOCX) [file pone.0196245.s008.docx]

**S4 Table**. **Functional annotation of the *ROBO1* variants**; output generated using HaploReg and RegulomeDB

| CHR | Position  (hg38) | Variant | GENCODE genes | dbSNP  annotation | Promoter histone marks | Enhancer histone marks | DNase | Proteins bound | Motifs changed^1^ | GWAS^2^ hits | GRASP^3^ QTL hits | Selected^4^ eQTL hits | RDB^5^ score |
| --- | --- | --- | --- | --- | --- | --- | --- | --- | --- | --- | --- | --- | --- |
| 3 | 79654398 | rs6419737 | ROBO1 | intronic |  |  |  |  | Sox |  |  |  | 7 |
| 3 | 79656029 | rs6775448 | ROBO1 | intronic |  |  |  |  | 5 altered motifs |  |  |  | 5 |
| 3 | 79656391 | rs4856228 | ROBO1 | intronic |  |  |  |  |  |  |  |  | 6 |
| 3 | 79656909 | rs4856433 | ROBO1 | intronic |  |  |  |  | 5 altered motifs |  |  |  | 7 |
| 3 | 79656982 | rs4856434 | ROBO1 | intronic |  |  |  |  | 4 altered motifs |  | 1 hit |  | 6 |
| 3 | 79657018 | rs9870711 | ROBO1 | intronic |  |  |  |  | Sox |  |  |  | 7 |
| 3 | 79657854 | rs4856440 | ROBO1 | intronic |  |  |  |  | 4 altered motifs |  |  |  | 6 |
| 3 | 79658000 | rs4856443 | ROBO1 | intronic |  |  |  |  | ELF1,Ik-2,NF-AT |  |  |  | 6 |
| 3 | 79658032 | rs4856444 | ROBO1 | intronic |  |  |  |  | 5 altered motifs |  |  |  | 7 |
| 3 | 79658207 | rs4856447 | ROBO1 | intronic |  |  |  |  | GATA |  |  |  | 7 |
| 3 | 79658237 | rs4856448 | ROBO1 | intronic |  |  |  |  | SEF-1 |  |  |  | 7 |
| 3 | 79658303 | rs7628280 | ROBO1 | intronic |  |  |  |  | Pax-4,p300 |  |  |  | 7 |
| 3 | 79658455 | rs7640127 | ROBO1 | intronic |  |  |  |  | Cart1,Pax7,Pou2f2 |  |  |  | 6 |
| 3 | 79658667 | rs9880911 | ROBO1 | intronic |  |  |  |  | 15 altered motifs |  |  |  | 6 |
| 3 | 79659331 | rs7431260 | ROBO1 | intronic |  |  |  |  | 6 altered motifs |  |  |  | 6 |
| 3 | 79660154 | rs6771093 | ROBO1 | intronic |  |  |  |  | CEBPB,Myc,p300 |  |  |  | 5 |
| 3 | 79660693 | rs7428022 | ROBO1 | intronic |  |  |  |  | Irx,Myc,XBP-1 |  |  |  | 6 |
| 3 | 79663672 | rs9869899 | ROBO1 | intronic |  |  |  |  | 6 altered motifs |  |  |  | 6 |
| 3 | 79665372 | rs9824870 | ROBO1 | intronic |  |  |  |  |  |  |  |  | 7 |
| 3 | 79665614 | rs9825204 | ROBO1 | intronic |  |  |  |  |  |  |  |  | 7 |
| 3 | 79666050 | rs9825870 | ROBO1 | intronic |  |  |  |  | 4 altered motifs |  |  |  | 6 |
| 3 | 79666710 | rs9857798 | ROBO1 | intronic |  |  |  |  | GR |  |  |  | 7 |
| 3 | 79667390 | rs6770961 | ROBO1 | intronic |  |  |  |  | 5 altered motifs |  |  |  | 6 |
| 3 | 79669912 | rs4856253 | ROBO1 | intronic |  |  |  |  | 5 altered motifs |  |  |  | 7 |
| 3 | 79670486 | rs9878764 | ROBO1 | intronic |  |  |  | CEBPB | 4 altered motifs |  |  |  | 5 |
| 3 | 79671635 | rs4856257 | ROBO1 | intronic |  |  |  |  | GCNF |  |  |  | 6 |
| 3 | 79678128 | rs3923148 | ROBO1 | intronic |  |  |  |  | Cdx |  |  |  | 7 |
| 3 | 79679580 | rs6548648 | ROBO1 | intronic |  |  |  |  | 7 altered motifs |  |  |  | 6 |
| 3 | 79680797 | rs6762755 | ROBO1 | intronic |  |  |  |  | GCM |  |  |  | 7 |
| 3 | 79681455 | rs7635296 | ROBO1 | intronic |  |  |  |  | HNF4 |  |  |  | 5 |
| 3 | 79681671 | rs7613379 | ROBO1 | intronic |  |  |  |  | p300 |  |  |  | 7 |
| 3 | 79681722 | rs7635587 | ROBO1 | intronic |  |  |  |  | EBF |  |  |  | 7 |
| 3 | 79688371 | rs7429100 | ROBO1 | intronic |  |  |  |  | 6 altered motifs |  |  |  | 7 |
| 3 | 79689074 | rs12107379 | ROBO1 | intronic |  |  |  |  | Mef2,TATA |  |  |  | 6 |
| 3 | 79689449 | rs13060599 | ROBO1 | intronic |  |  |  |  | PLZF,Pou2f2 |  |  |  | 6 |
| 3 | 79690952 | rs9631514 | ROBO1 | intronic |  |  |  |  | DMRT1 |  |  |  | 7 |
| 3 | 79695773 | rs10212228 | ROBO1 | intronic |  |  |  |  | CEBPB |  |  |  | 6 |
| 3 | 79697112 | rs7431092 | ROBO1 | intronic |  |  |  |  | AIRE,EBF,Myc |  |  |  | 7 |
| 3 | 79697440 | rs9862551 | ROBO1 | intronic |  |  |  |  | Maf,YY1 |  |  |  | 7 |
| 3 | 79698577 | rs9812795 | ROBO1 | intronic |  |  |  |  | 6 altered motifs |  |  |  | 7 |
| 3 | 79699328 | rs9873237 | ROBO1 | intronic |  |  |  |  | 5 altered motifs |  |  |  | 6 |
| 3 | 79717361 | rs7430339 | ROBO1 | intronic |  |  |  |  | Sox |  |  |  | 7 |
| 3 | 79733987 | rs4856298 | ROBO1 | intronic |  |  |  |  | STAT |  |  |  | 7 |
| 3 | 79735900 | rs7426689 | ROBO1 | intronic |  |  |  |  | 15 altered motifs |  |  |  | 6 |
| 3 | 79735935 | rs7430639 | ROBO1 | intronic |  |  |  |  | 4 altered motifs |  |  |  | 6 |
| 3 | 79736068 | rs7431063 | ROBO1 | intronic |  |  |  |  | Foxa |  |  |  | 7 |
| 3 | 79740034 | rs3924599 | ROBO1 | intronic |  |  |  |  | 10 altered motifs |  |  |  | 6 |
| 3 | 79740258 | rs9309831 | ROBO1 | intronic |  |  |  |  | Nanog,Pou2f2 |  |  |  | 7 |
| 3 | 79740338 | rs9309832 | ROBO1 | intronic |  |  |  |  | 8 altered motifs |  |  |  | 6 |
| 3 | 79740495 | rs7426439 | ROBO1 | intronic |  |  |  |  | 6 altered motifs |  |  |  | 7 |
| 3 | 79741257 | rs1995402 | ROBO1 | intronic |  |  |  |  | 5 altered motifs |  | 3 hits |  | 6 |
| 3 | 79741283 | rs1995401 | ROBO1 | intronic |  |  |  |  | Osr,Pou3f2 |  |  |  | 6 |
| 3 | 79742264 | rs7649774 | ROBO1 | intronic |  |  |  |  | THAP1 |  |  |  | 7 |

^1^Motifs changed= effect of SNPs on regulatory motifs; ^2^GWAS hits from the National Human Genome Research Institute (NHGRI) catalog; ^3^GRASP=Genome-wide Repository of Associations between SNPs and Phenotypes, an omic tool, eQTL= expression quantitative trait locus; ^4^Selected eQTL=curated within HaploReg from GTEx, GEUVADIS and other studies; ^5^RDB= RegulomeDB [1] score: 1f: Likely to affect binding and linked to expression of a gene target; 2b: Likely to affect binding; 3a: Less likely to affect binding; 4-6: minimal binding evidence.

1. Boyle AP, Hong EL, Hariharan M, Cheng Y, Schaub MA, Kasowski M, et al. Annotation of functional variation in personal genomes using RegulomeDB. Genome Res. 2012;22(9):1790-7. doi: 10.1101/gr.137323.112. PubMed PMID: 22955989; PubMed Central PMCID: PMCPMC3431494.
